# Supplementary figures and images for: LMAD-YOLO: A vehicle image detection algorithm for drone aerial photography based on multi-scale feature fusion
Source: PLoS One. 2025 Jul 15;20(7):e0328248. doi: 10.1371/journal.pone.0328248 (PMC12262879; doi:10.1371/journal.pone.0328248)

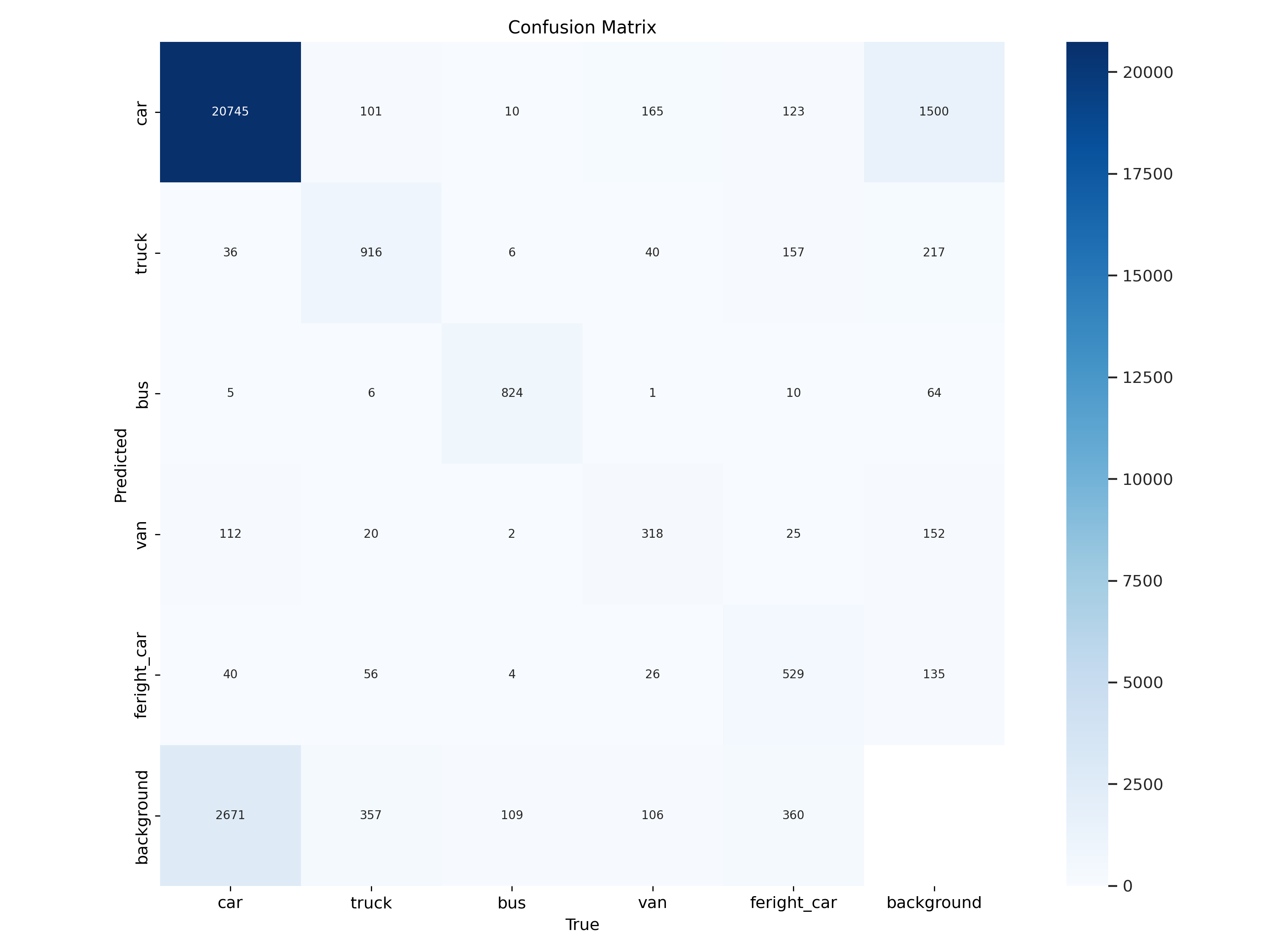

Supplement: S1 File — (ZIP) [file pone.0328248.s001.zip › S1 Model training result data/Drone Vehicle/Train/LMAD-YOLO11/confusion_matrix.png]

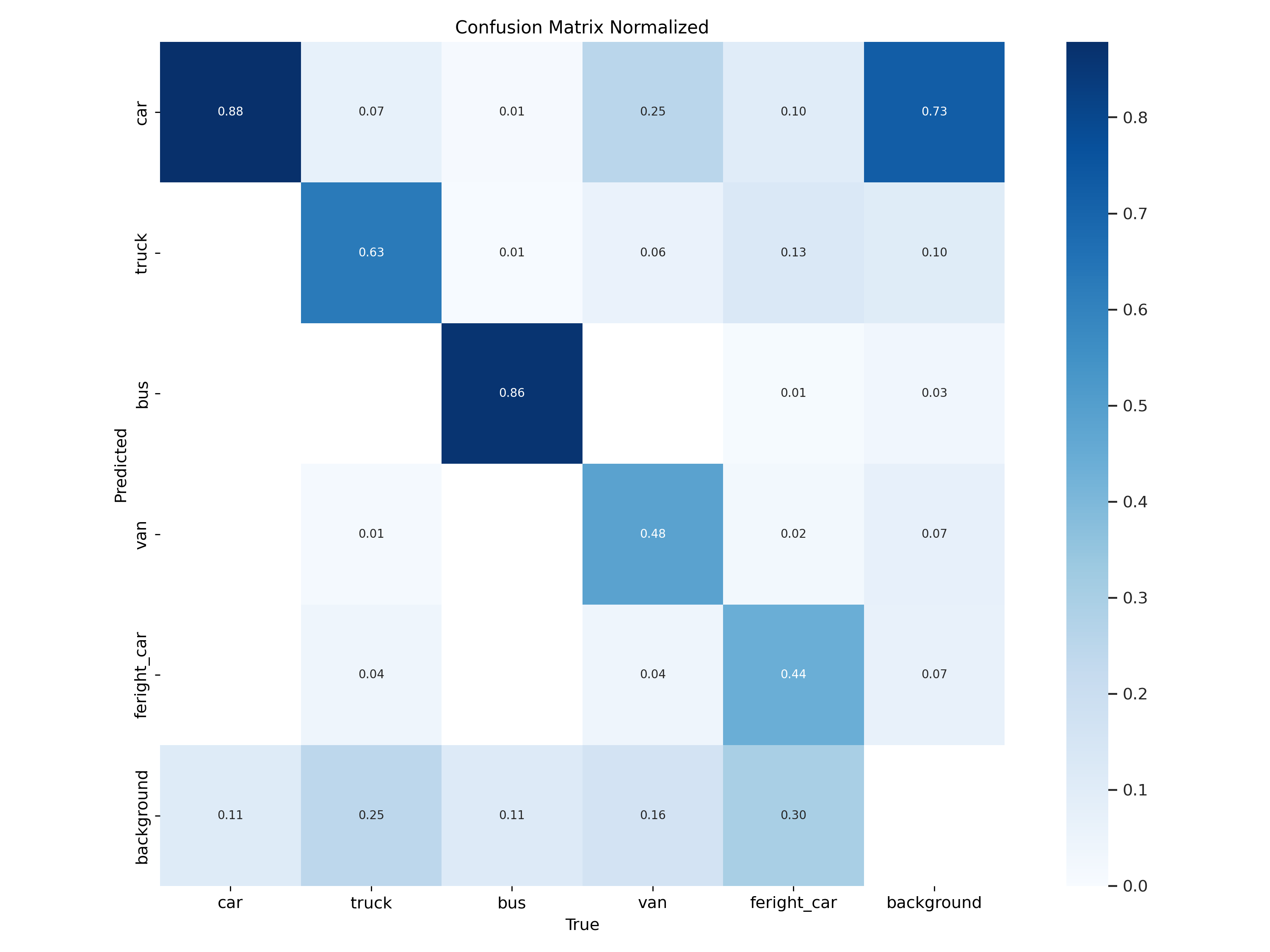

Supplement: S1 File — (ZIP) [file pone.0328248.s001.zip › S1 Model training result data/Drone Vehicle/Train/LMAD-YOLO11/confusion_matrix_normalized.png]

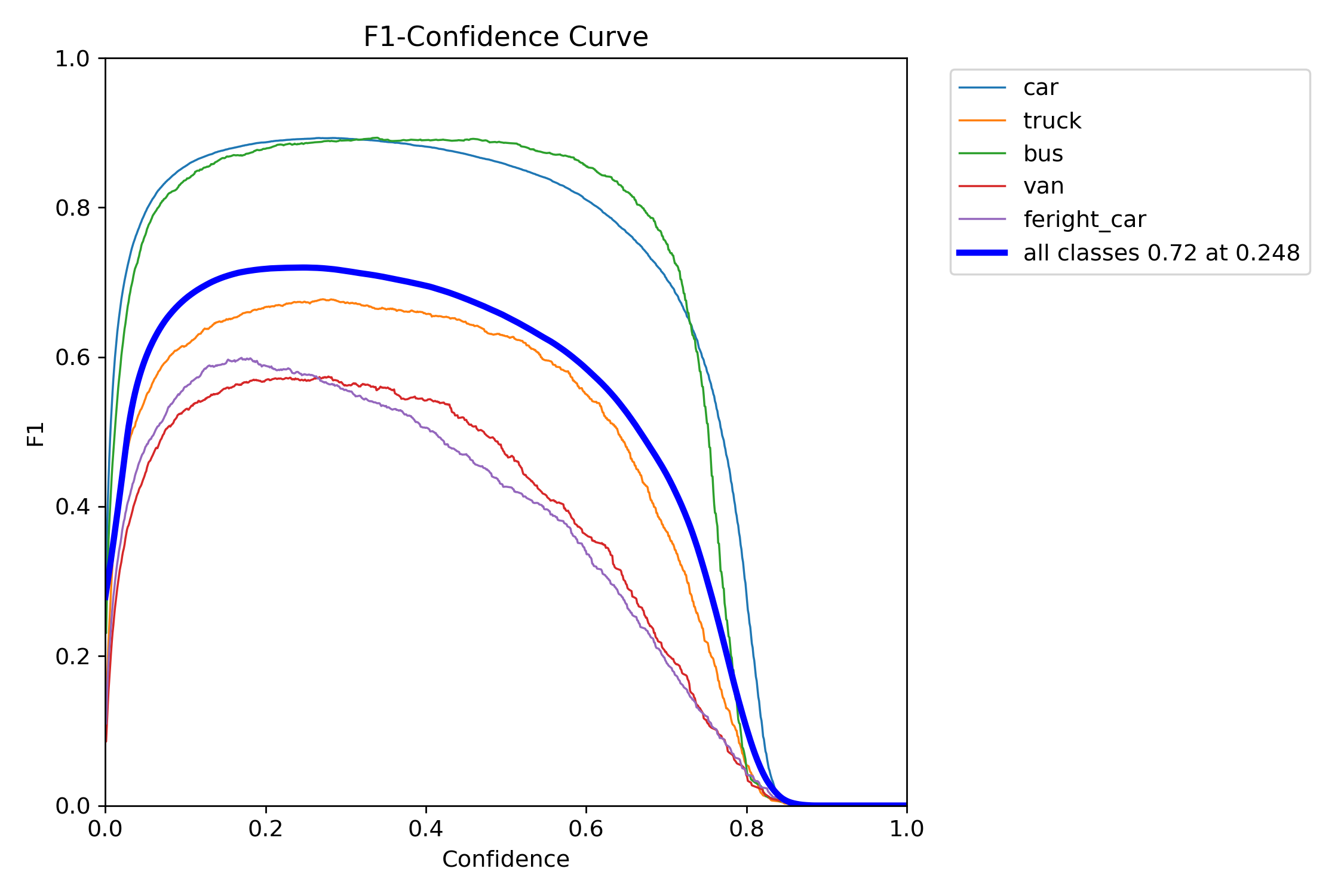

Supplement: S1 File — (ZIP) [file pone.0328248.s001.zip › S1 Model training result data/Drone Vehicle/Train/LMAD-YOLO11/F1_curve.png]

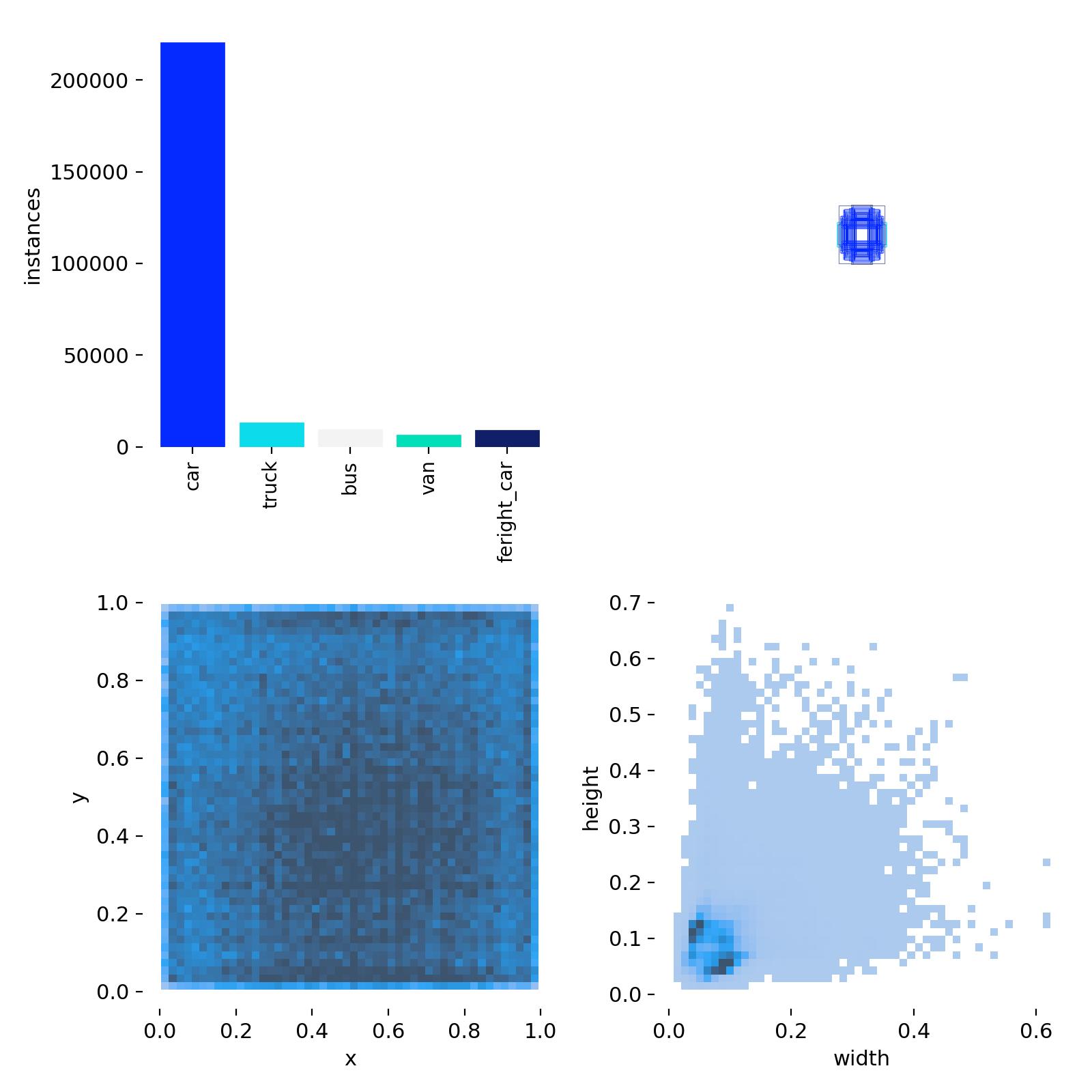

Supplement: S1 File — (ZIP) [file pone.0328248.s001.zip › S1 Model training result data/Drone Vehicle/Train/LMAD-YOLO11/labels.jpg]

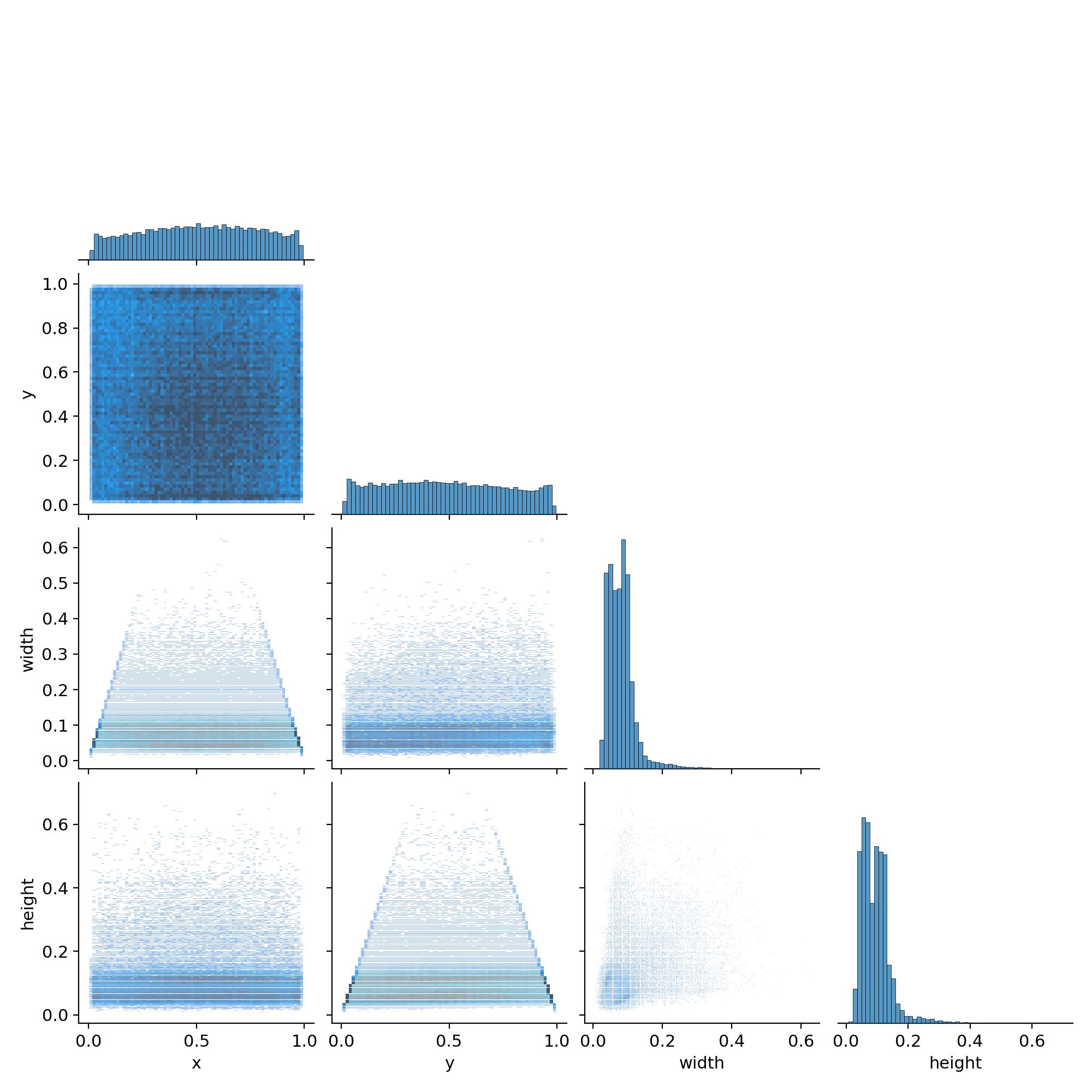

Supplement: S1 File — (ZIP) [file pone.0328248.s001.zip › S1 Model training result data/Drone Vehicle/Train/LMAD-YOLO11/labels_correlogram.jpg]

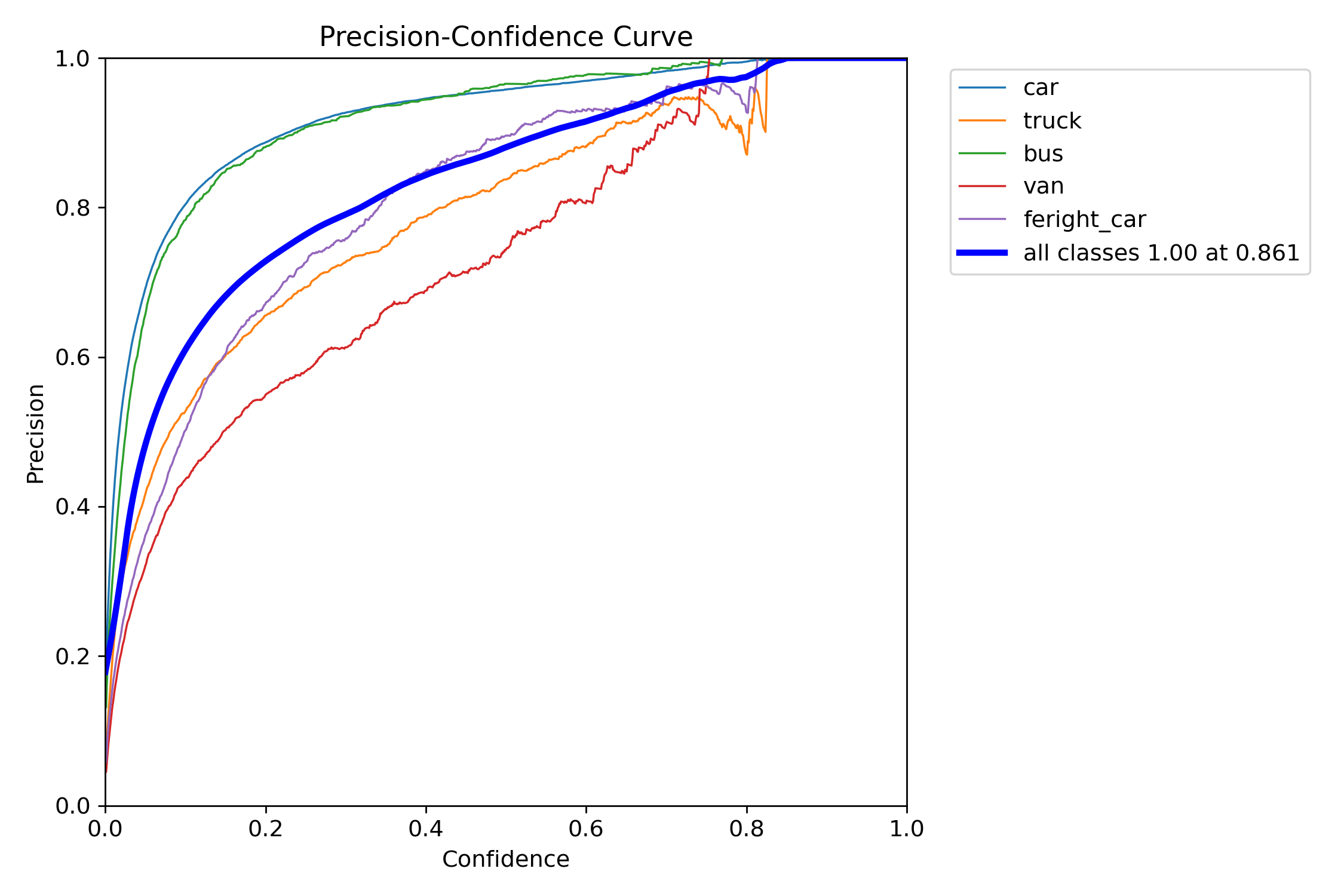

Supplement: S1 File — (ZIP) [file pone.0328248.s001.zip › S1 Model training result data/Drone Vehicle/Train/LMAD-YOLO11/P_curve.png]

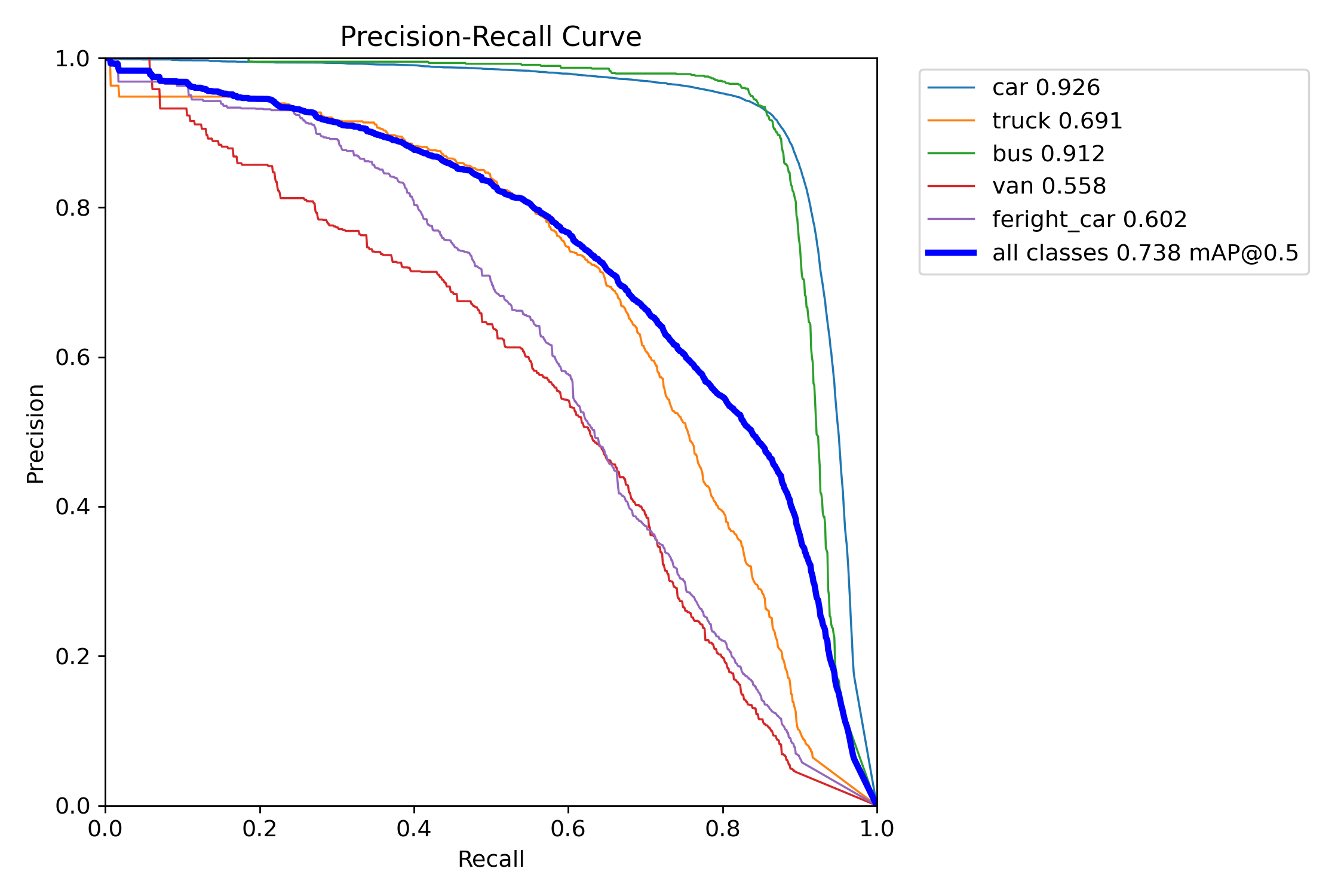

Supplement: S1 File — (ZIP) [file pone.0328248.s001.zip › S1 Model training result data/Drone Vehicle/Train/LMAD-YOLO11/PR_curve.png]

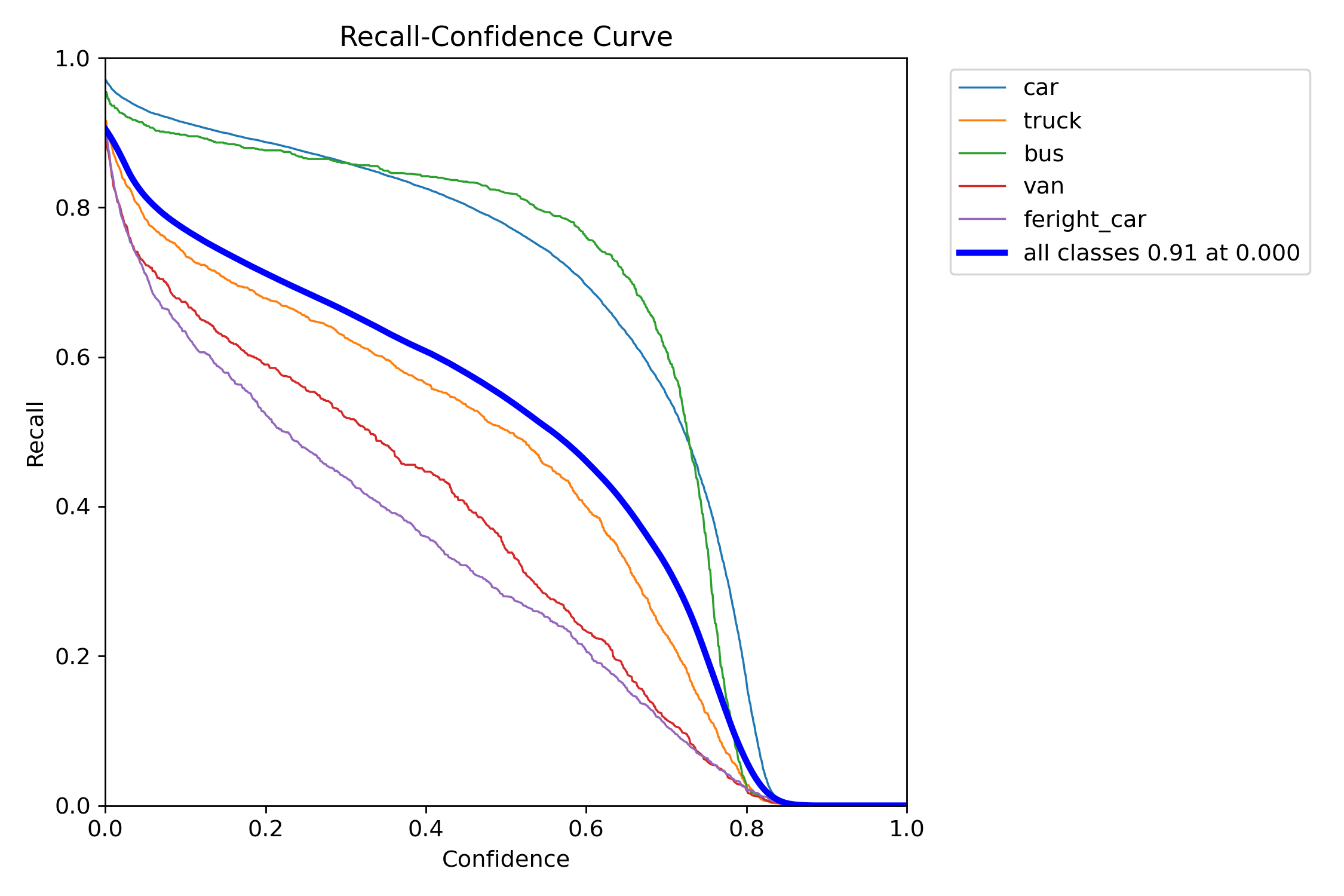

Supplement: S1 File — (ZIP) [file pone.0328248.s001.zip › S1 Model training result data/Drone Vehicle/Train/LMAD-YOLO11/R_curve.png]

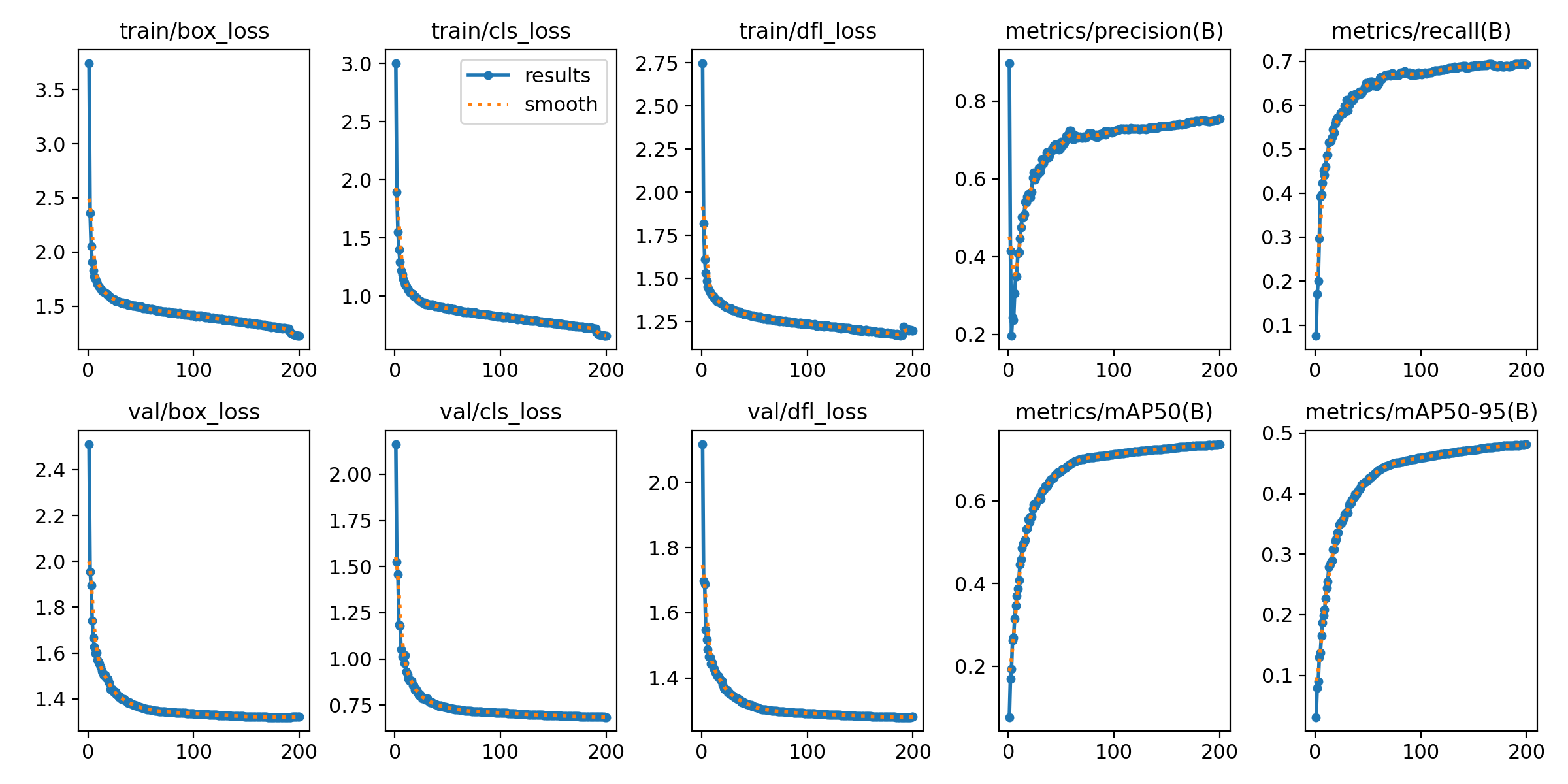

Supplement: S1 File — (ZIP) [file pone.0328248.s001.zip › S1 Model training result data/Drone Vehicle/Train/LMAD-YOLO11/results.png]

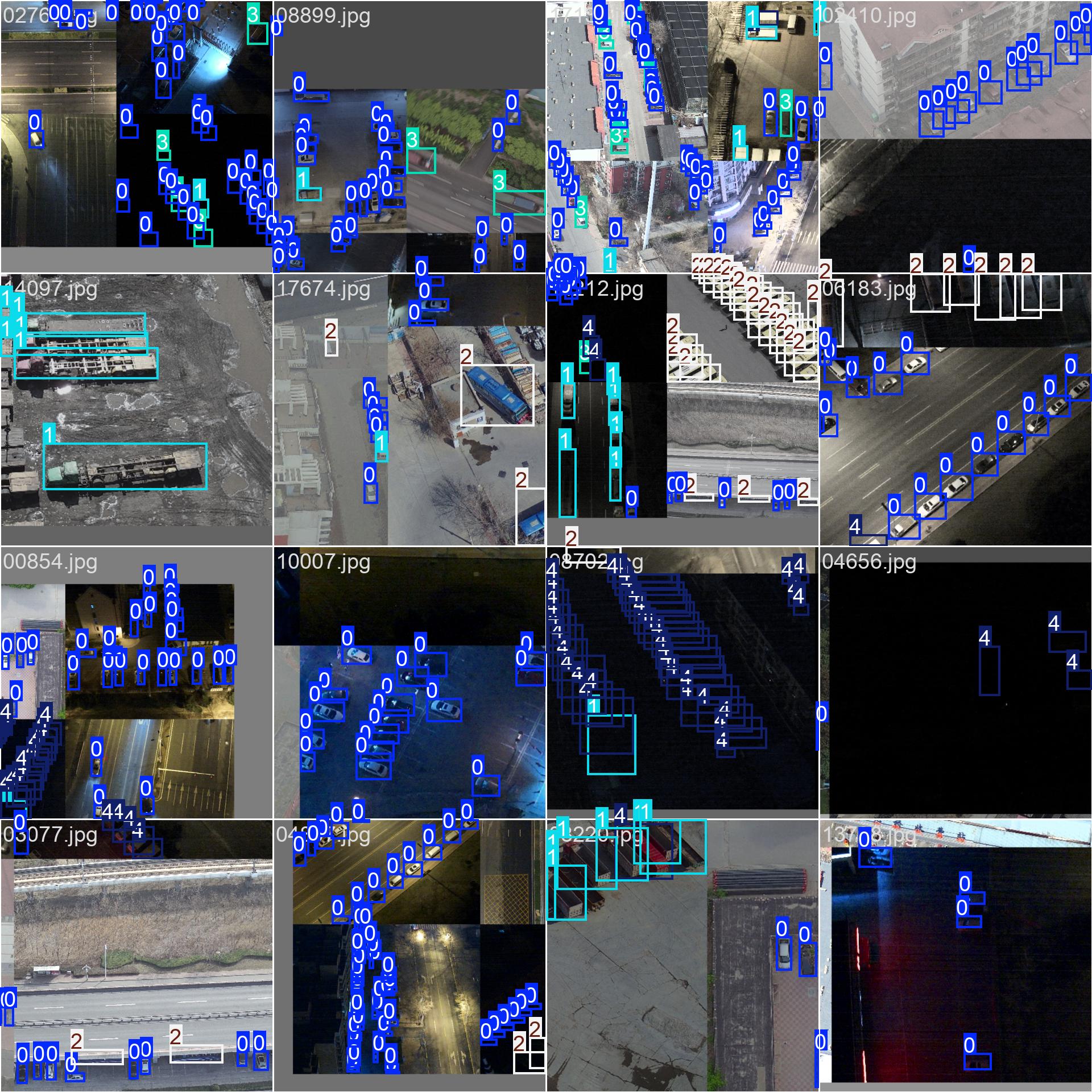

Supplement: S1 File — (ZIP) [file pone.0328248.s001.zip › S1 Model training result data/Drone Vehicle/Train/LMAD-YOLO11/train_batch0.jpg]

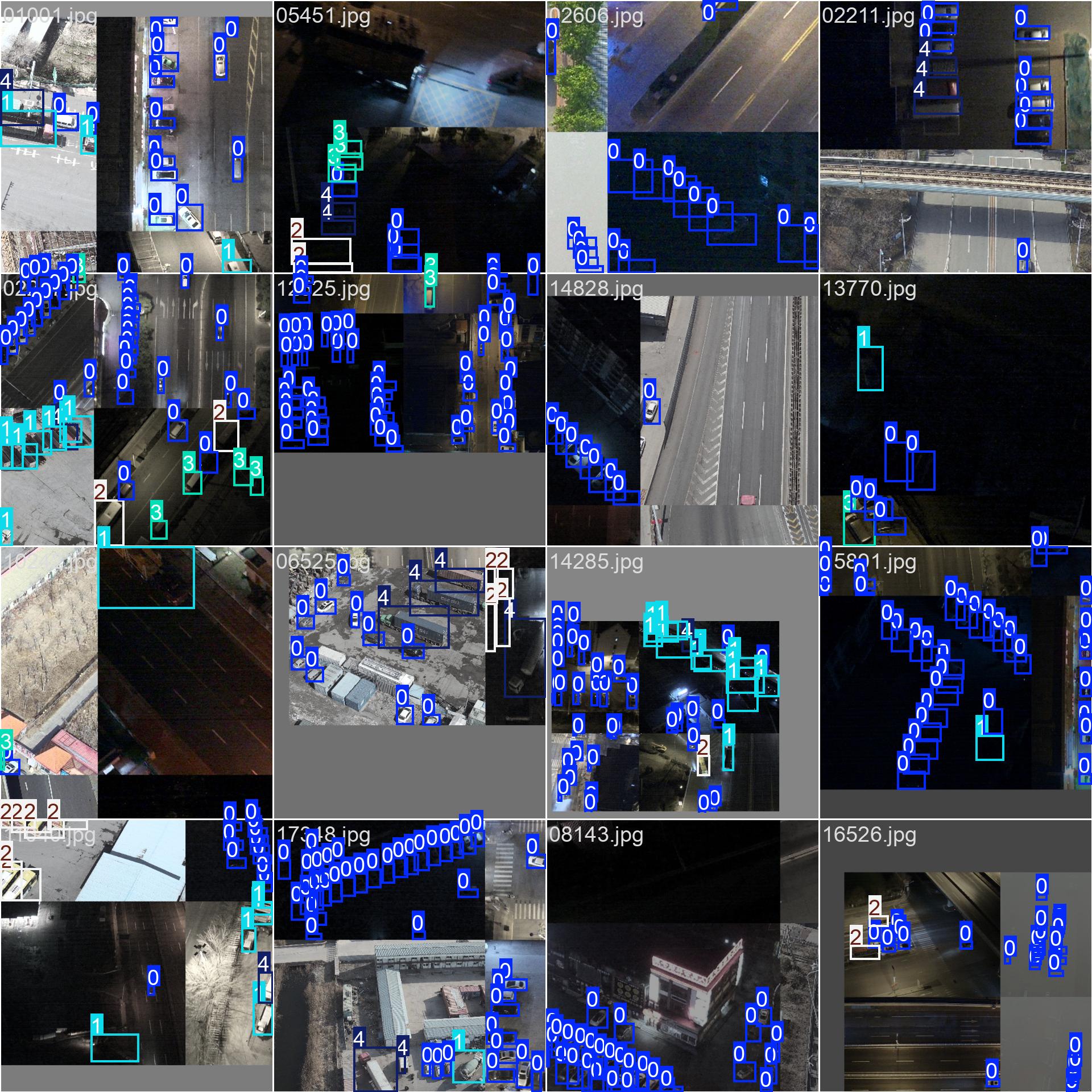

Supplement: S1 File — (ZIP) [file pone.0328248.s001.zip › S1 Model training result data/Drone Vehicle/Train/LMAD-YOLO11/train_batch1.jpg]

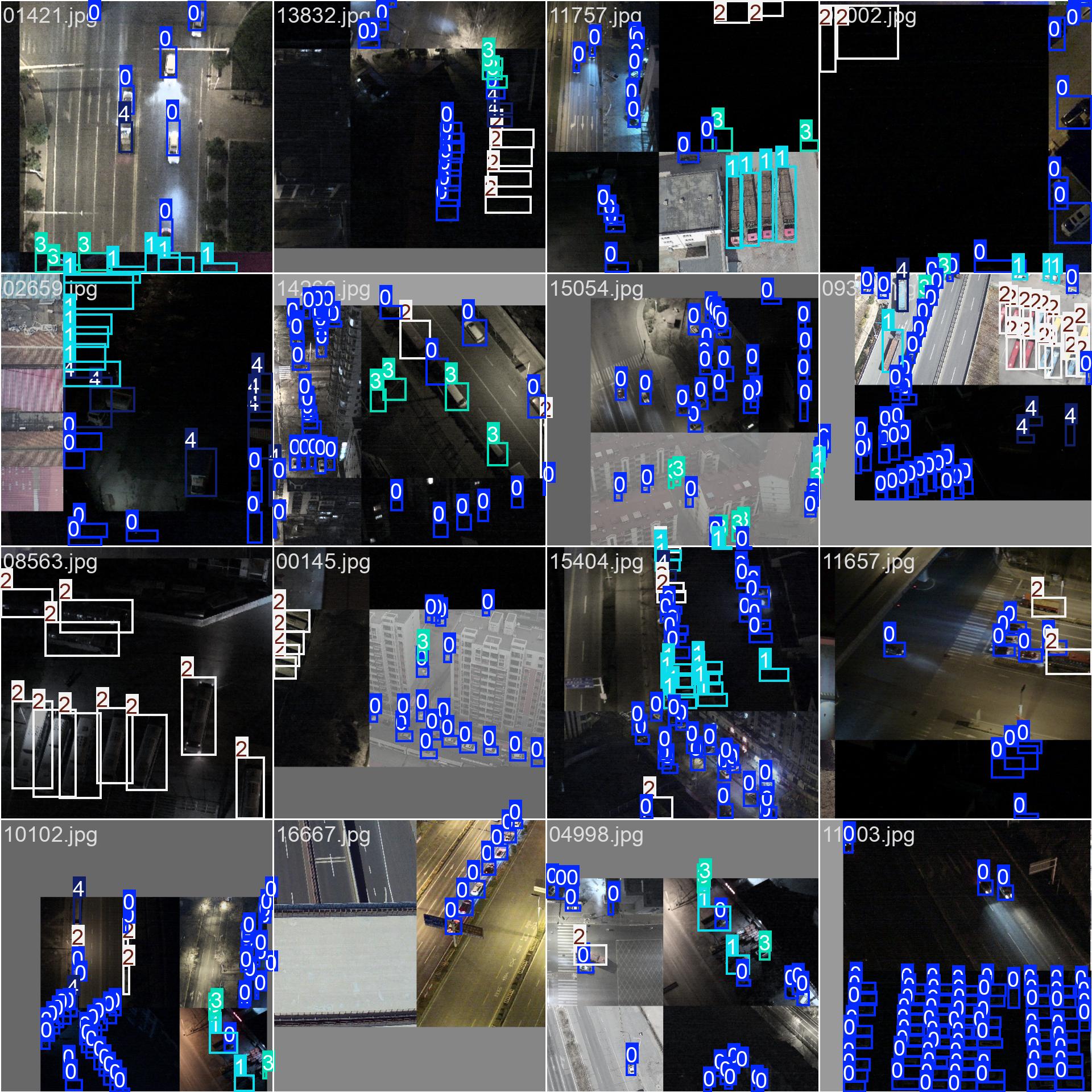

Supplement: S1 File — (ZIP) [file pone.0328248.s001.zip › S1 Model training result data/Drone Vehicle/Train/LMAD-YOLO11/train_batch2.jpg]

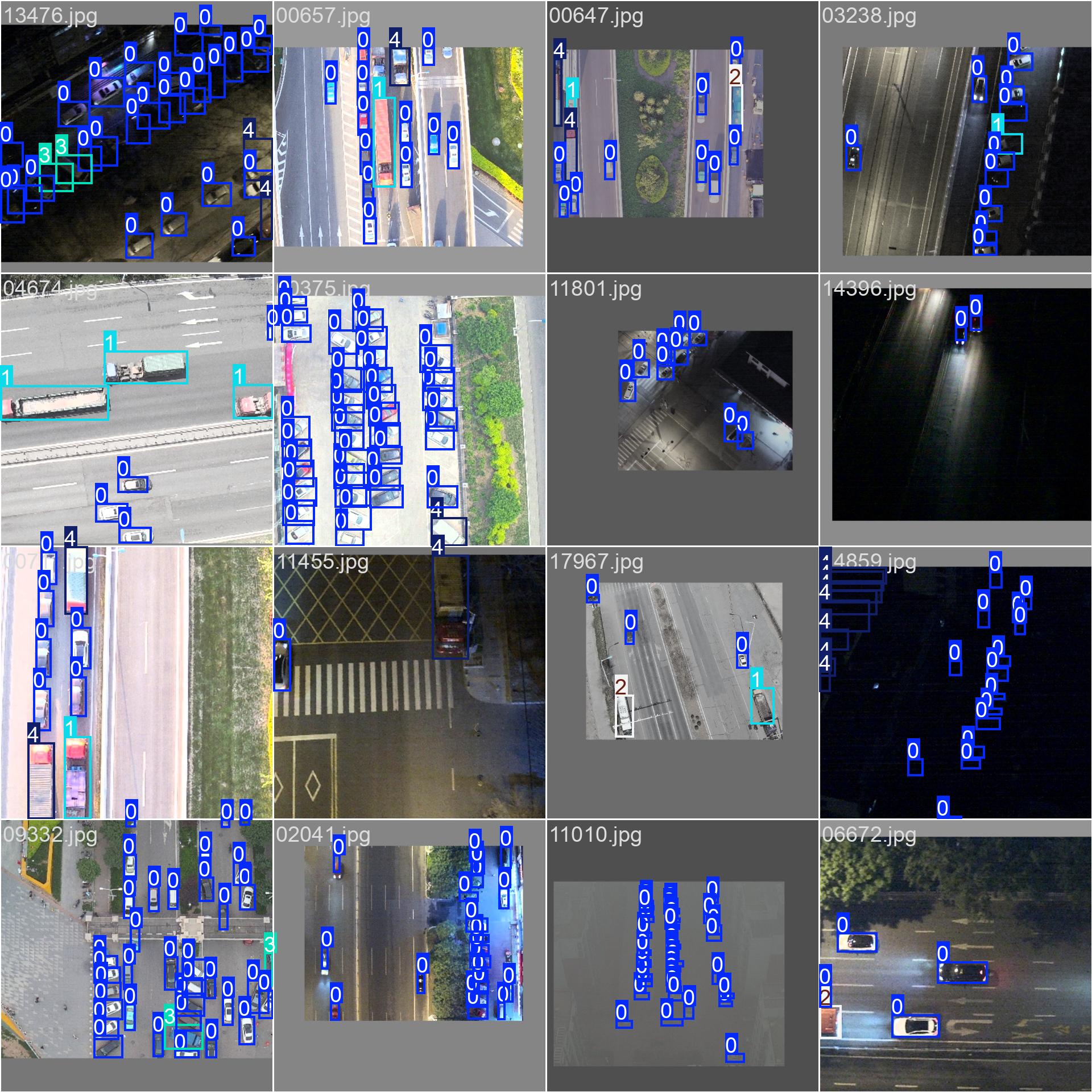

Supplement: S1 File — (ZIP) [file pone.0328248.s001.zip › S1 Model training result data/Drone Vehicle/Train/LMAD-YOLO11/train_batch86640.jpg]

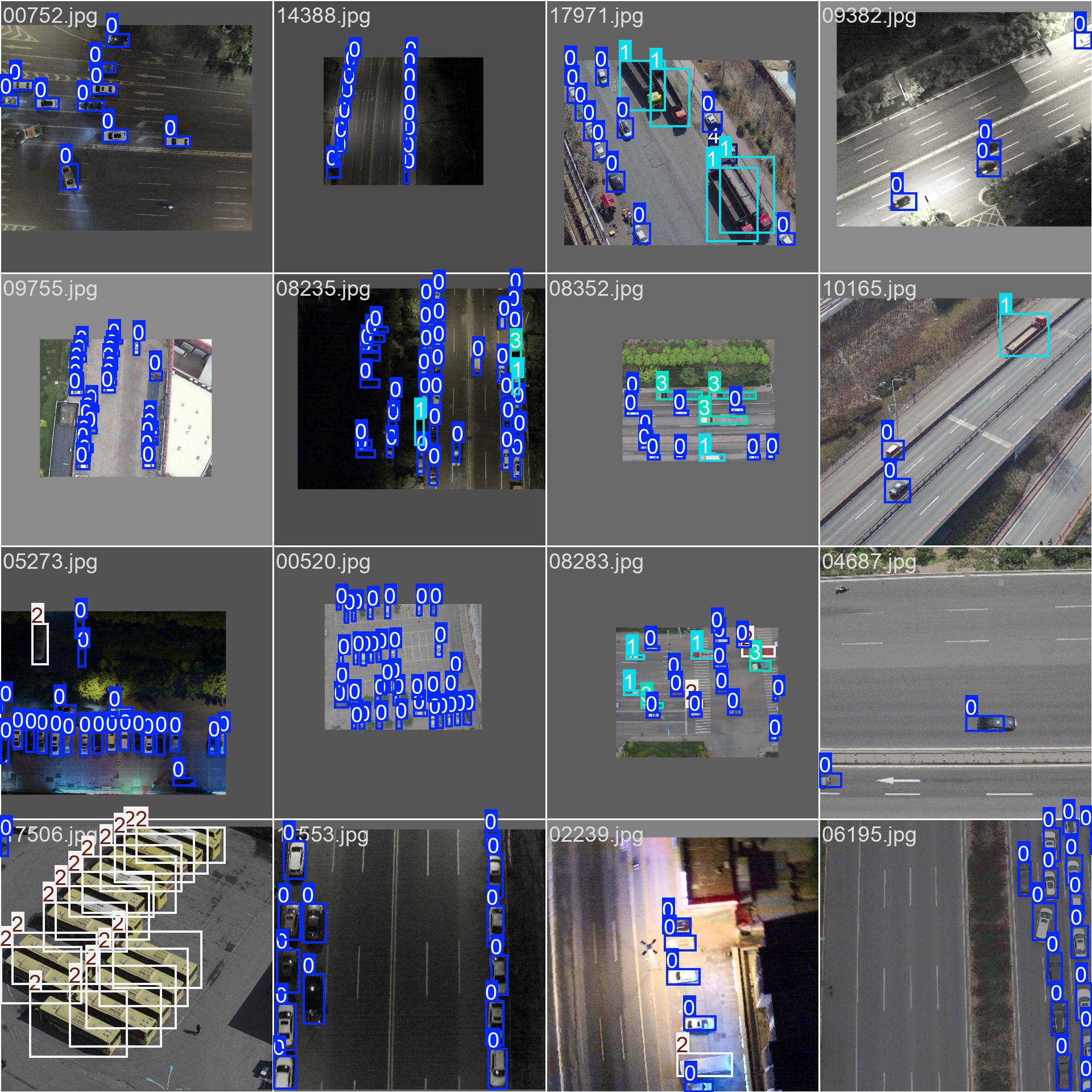

Supplement: S1 File — (ZIP) [file pone.0328248.s001.zip › S1 Model training result data/Drone Vehicle/Train/LMAD-YOLO11/train_batch86641.jpg]

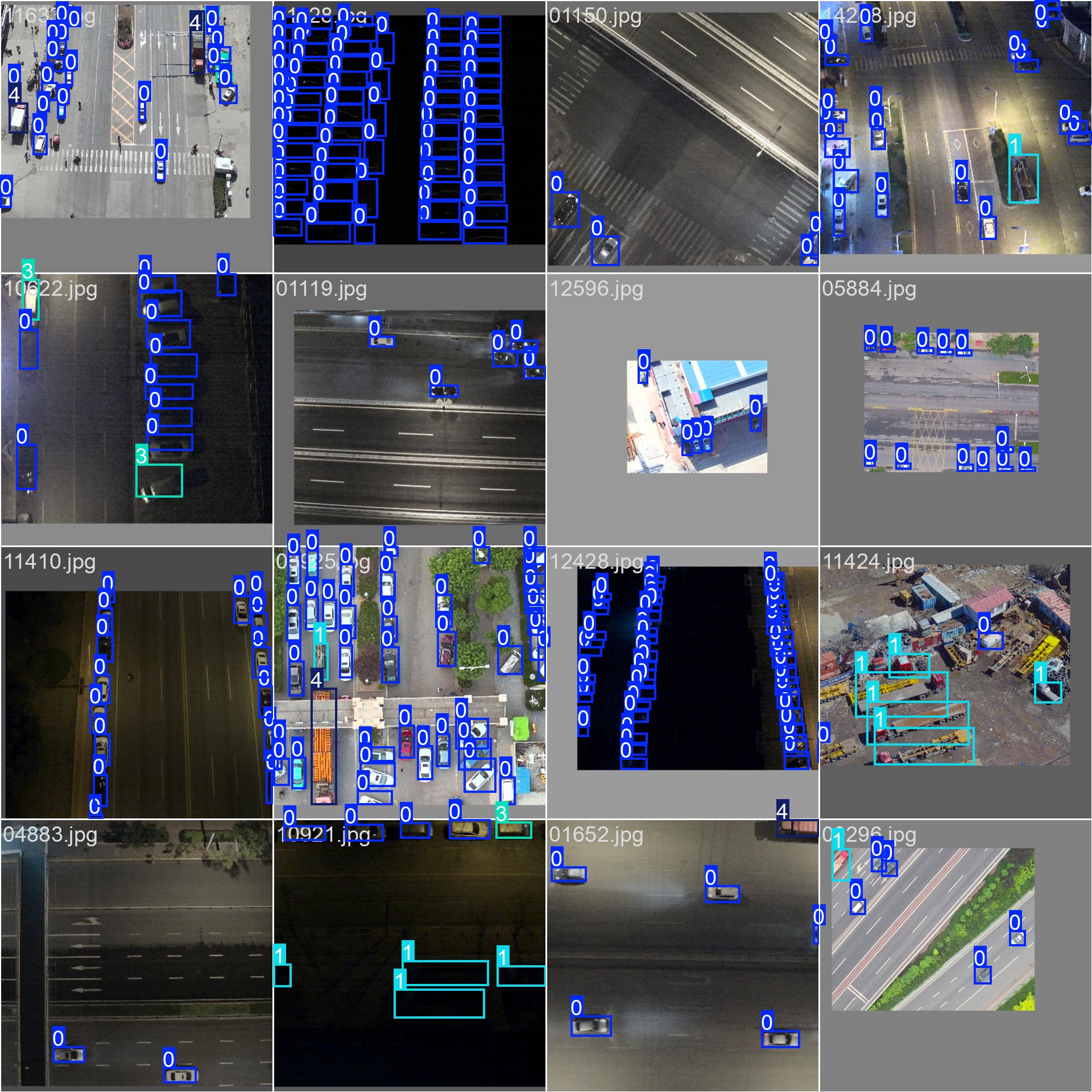

Supplement: S1 File — (ZIP) [file pone.0328248.s001.zip › S1 Model training result data/Drone Vehicle/Train/LMAD-YOLO11/train_batch86642.jpg]

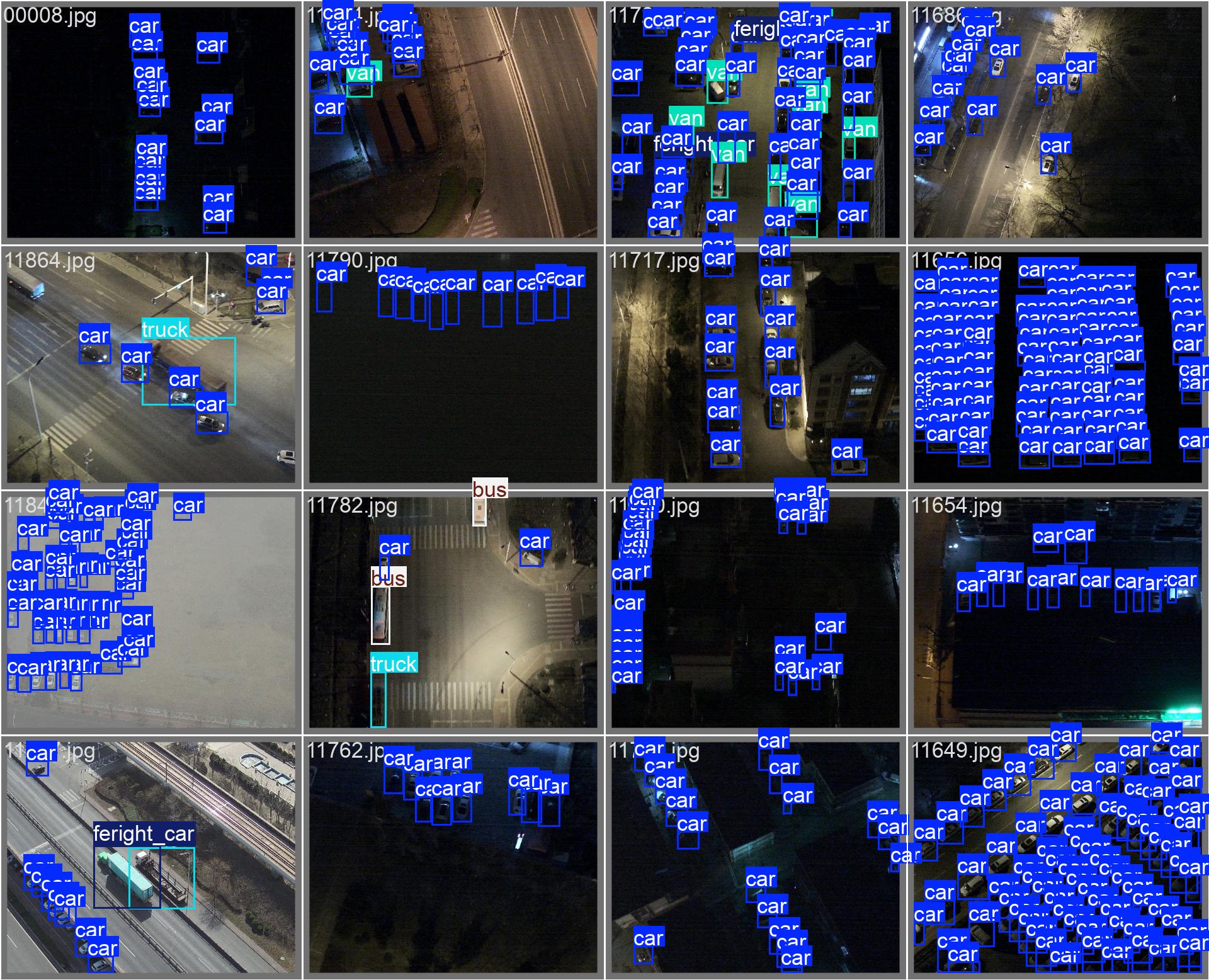

Supplement: S1 File — (ZIP) [file pone.0328248.s001.zip › S1 Model training result data/Drone Vehicle/Train/LMAD-YOLO11/val_batch0_labels.jpg]

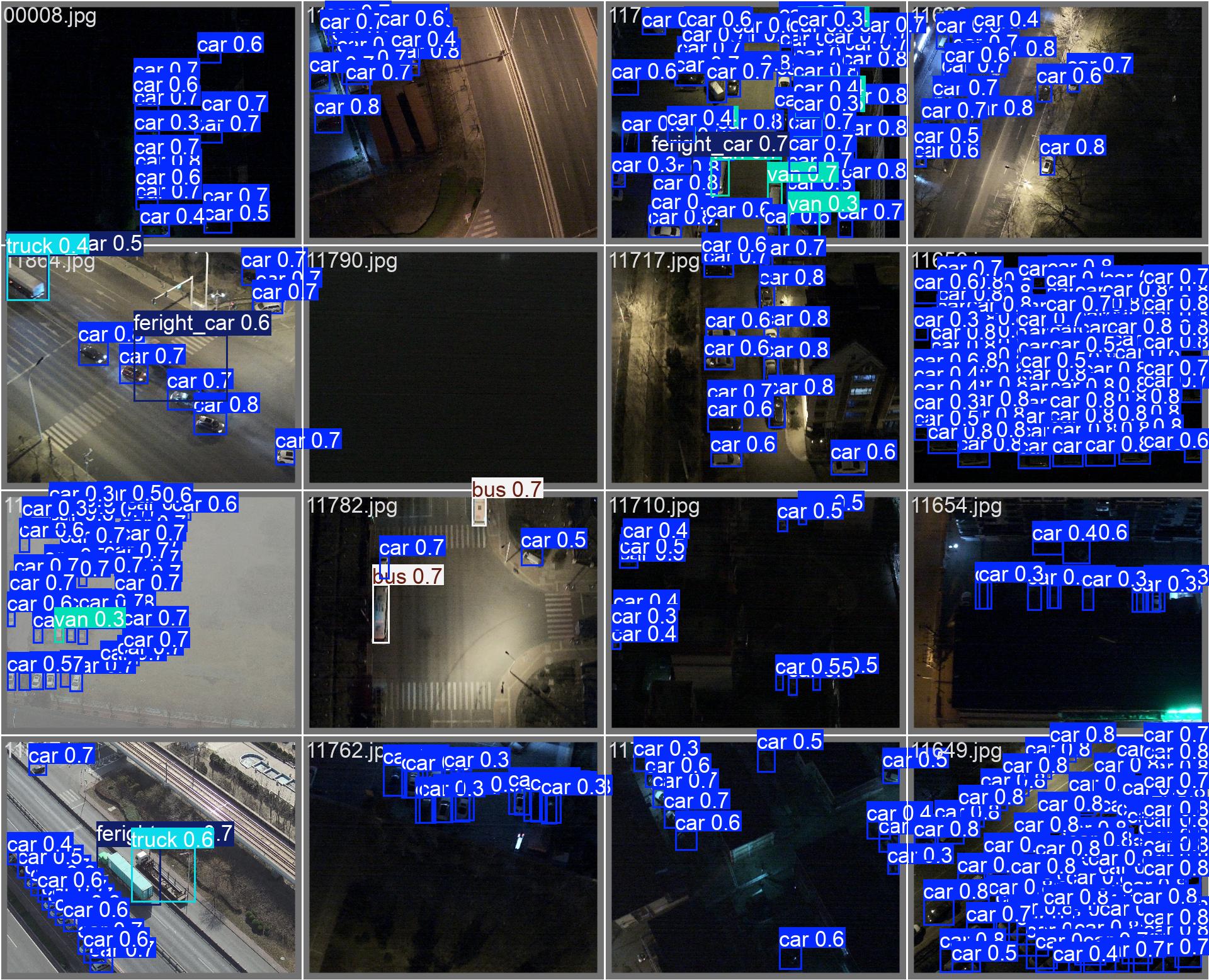

Supplement: S1 File — (ZIP) [file pone.0328248.s001.zip › S1 Model training result data/Drone Vehicle/Train/LMAD-YOLO11/val_batch0_pred.jpg]

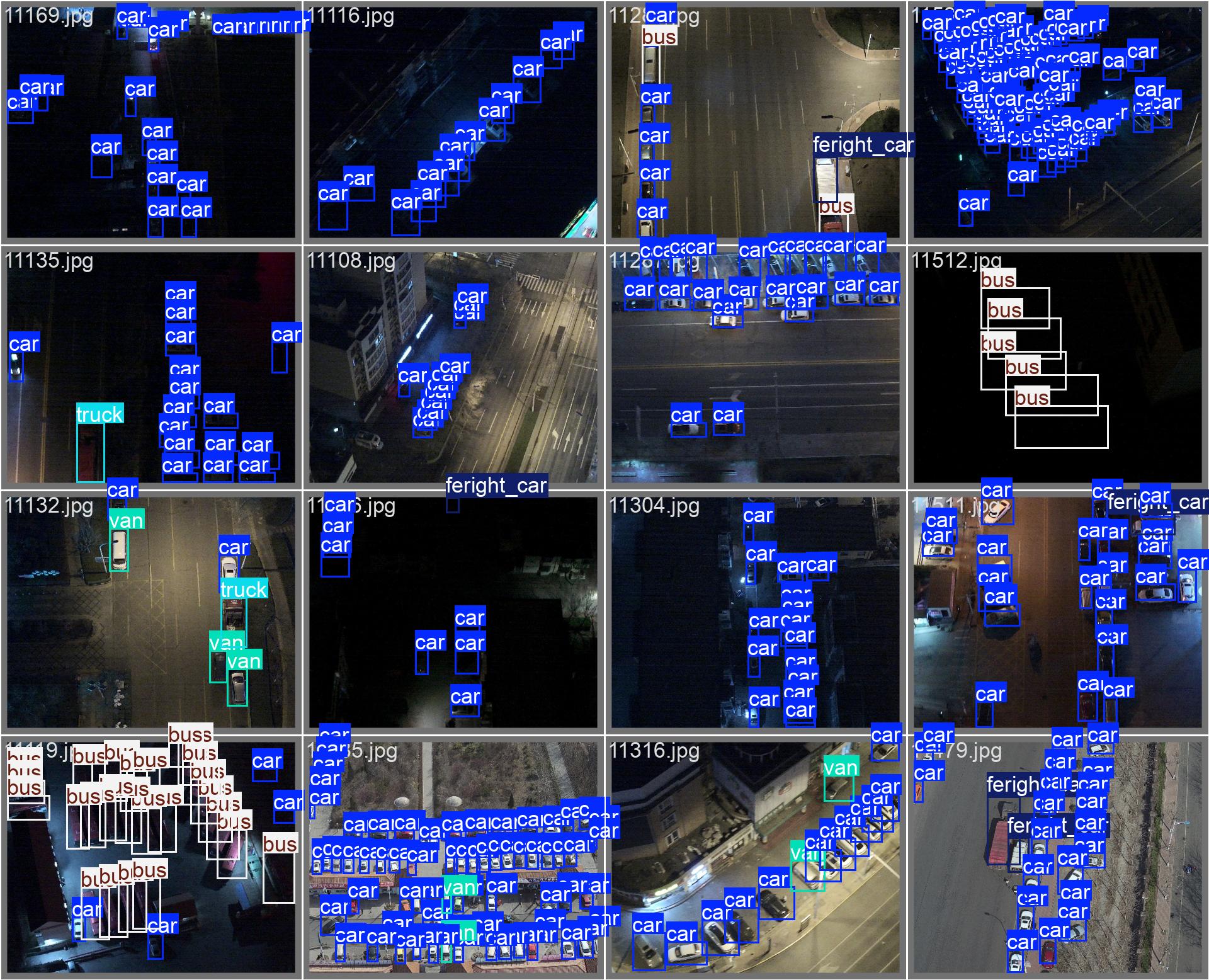

Supplement: S1 File — (ZIP) [file pone.0328248.s001.zip › S1 Model training result data/Drone Vehicle/Train/LMAD-YOLO11/val_batch1_labels.jpg]

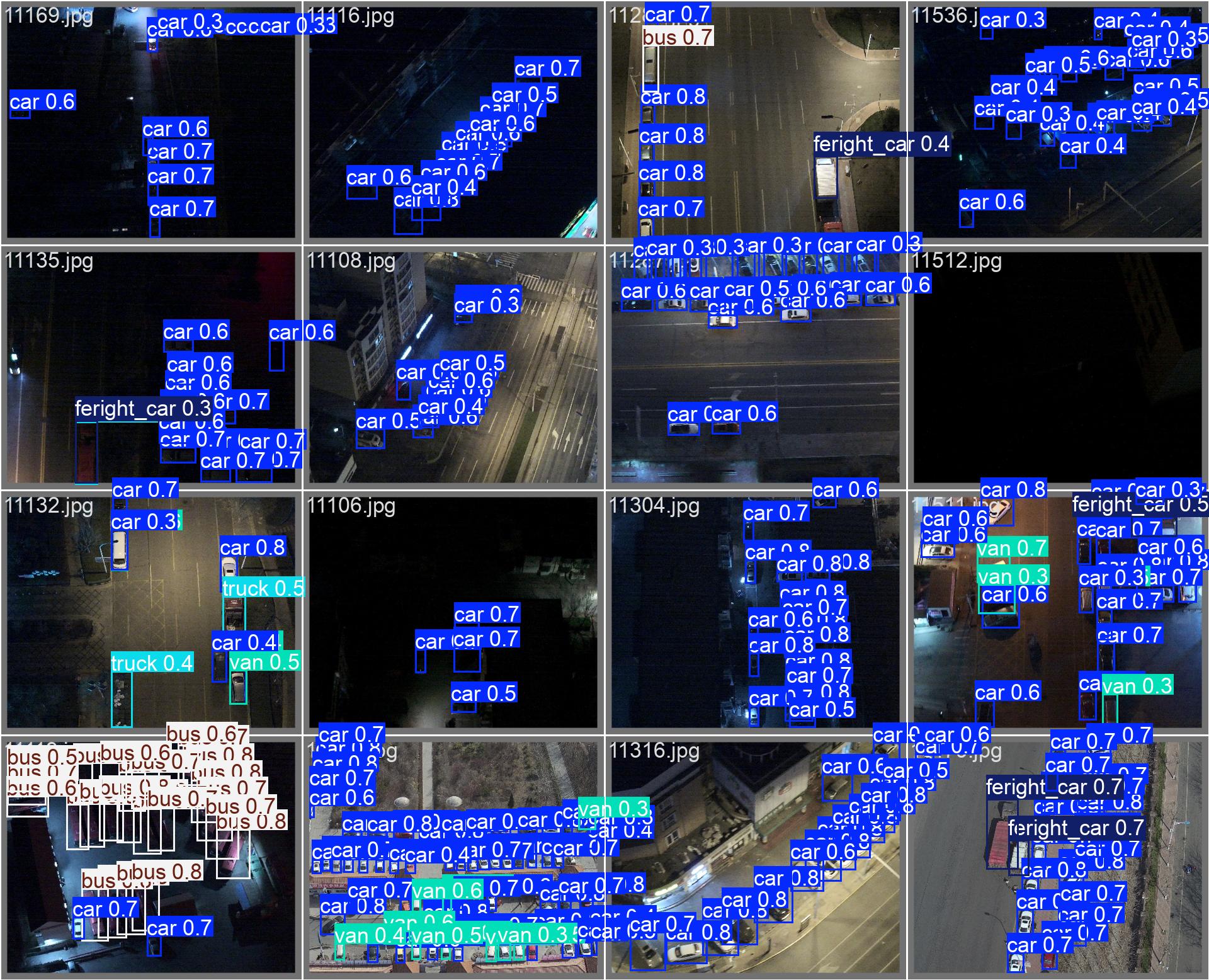

Supplement: S1 File — (ZIP) [file pone.0328248.s001.zip › S1 Model training result data/Drone Vehicle/Train/LMAD-YOLO11/val_batch1_pred.jpg]

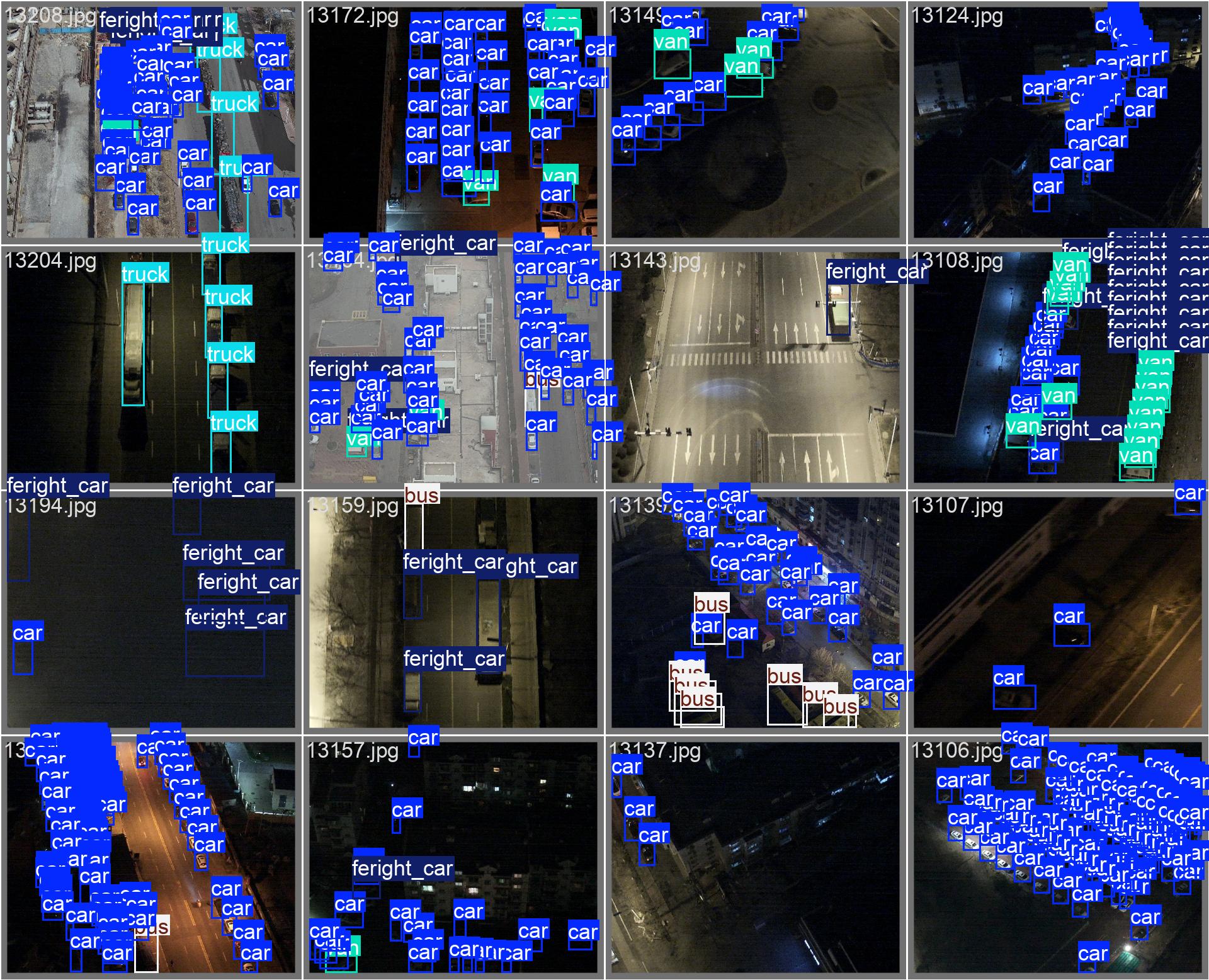

Supplement: S1 File — (ZIP) [file pone.0328248.s001.zip › S1 Model training result data/Drone Vehicle/Train/LMAD-YOLO11/val_batch2_labels.jpg]

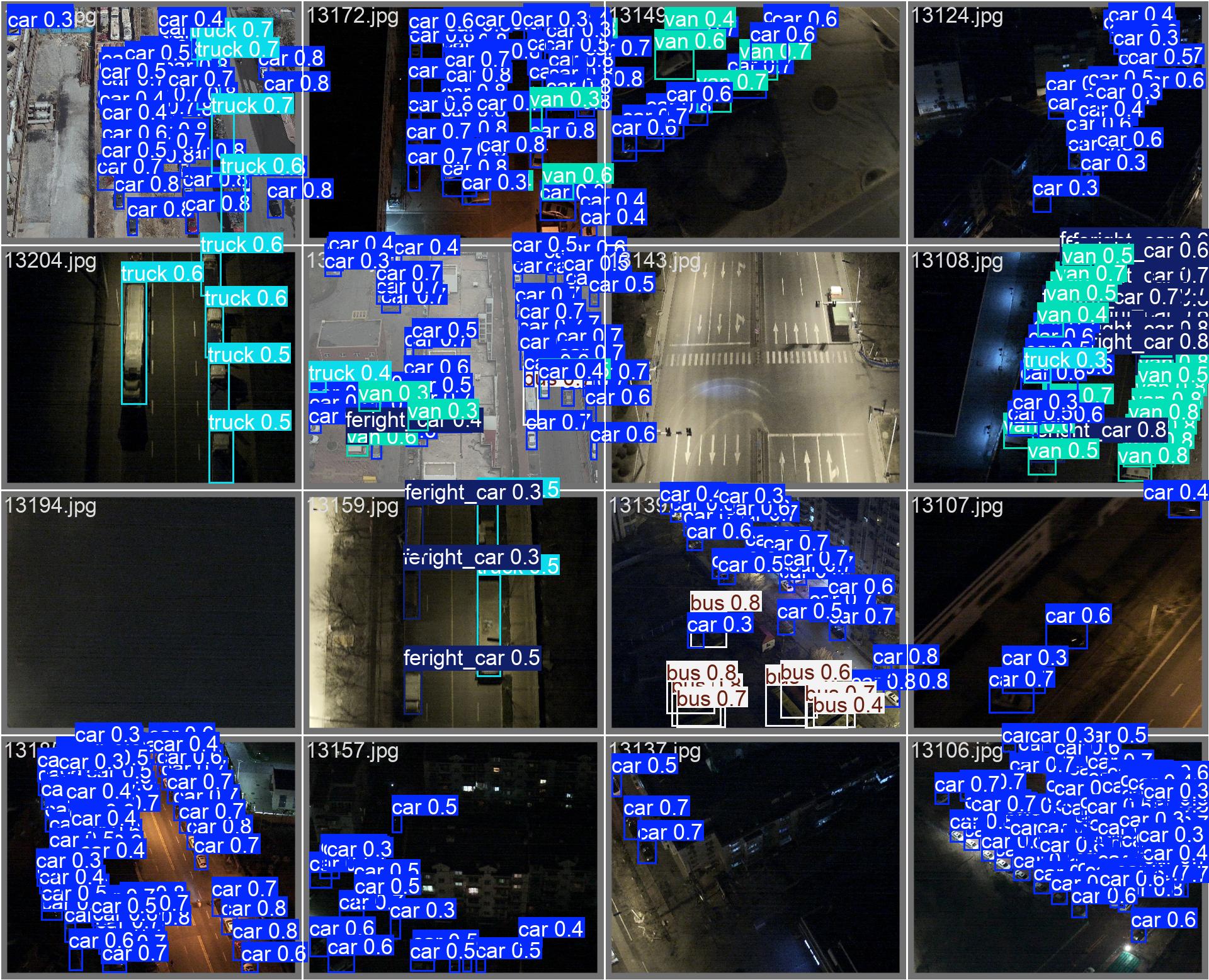

Supplement: S1 File — (ZIP) [file pone.0328248.s001.zip › S1 Model training result data/Drone Vehicle/Train/LMAD-YOLO11/val_batch2_pred.jpg]

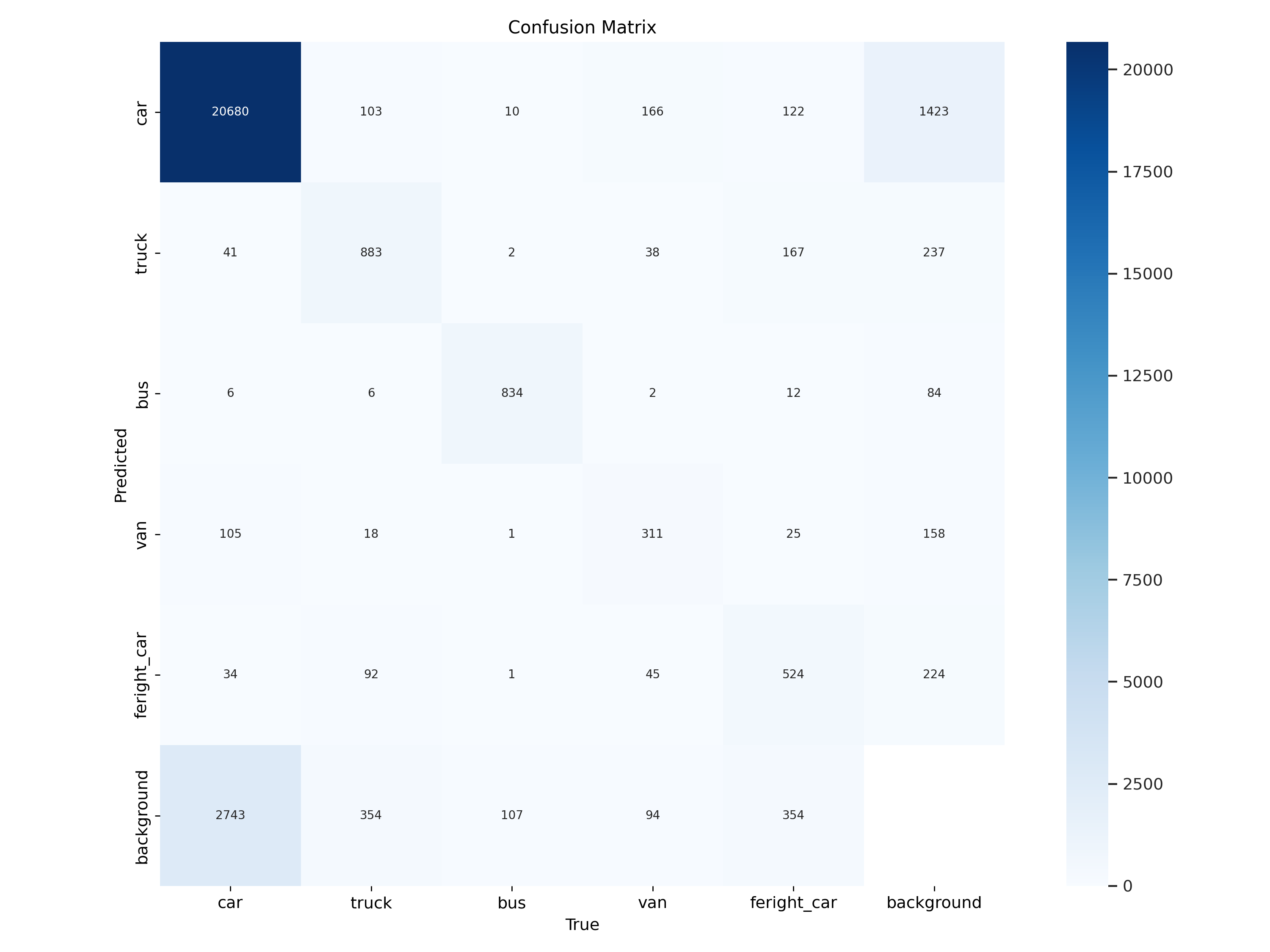

Supplement: S1 File — (ZIP) [file pone.0328248.s001.zip › S1 Model training result data/Drone Vehicle/Train/YOLO11/confusion_matrix.png]

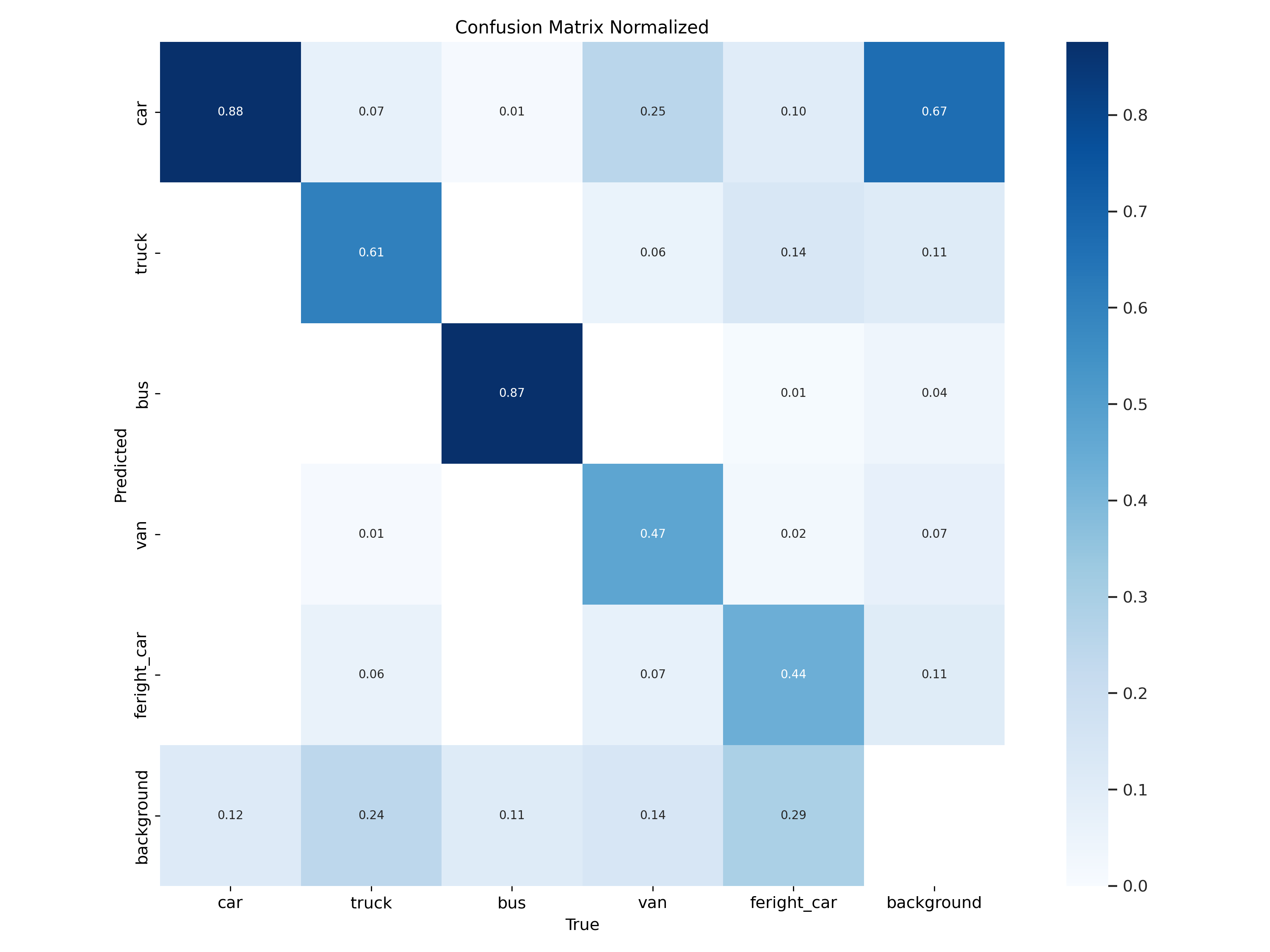

Supplement: S1 File — (ZIP) [file pone.0328248.s001.zip › S1 Model training result data/Drone Vehicle/Train/YOLO11/confusion_matrix_normalized.png]

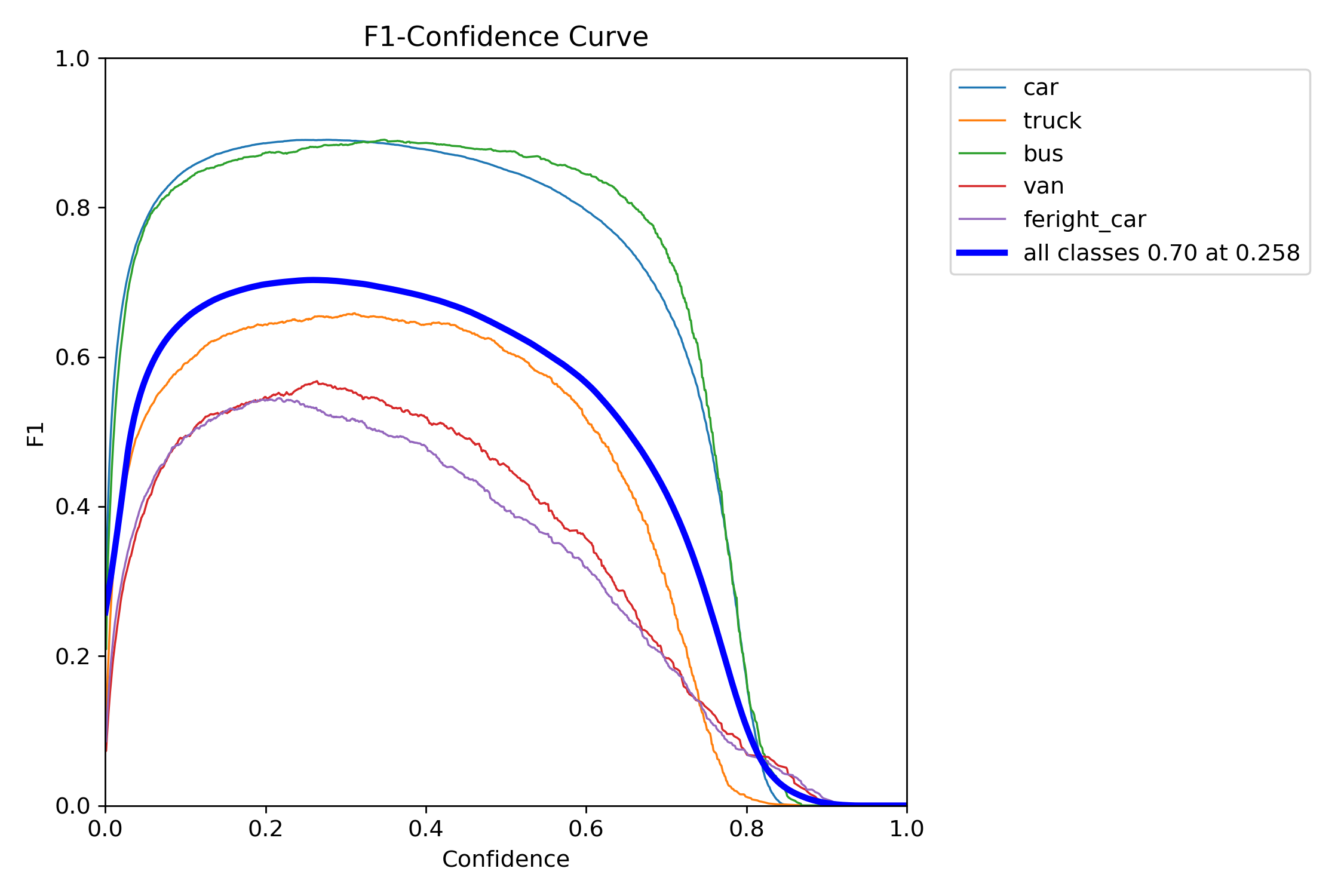

Supplement: S1 File — (ZIP) [file pone.0328248.s001.zip › S1 Model training result data/Drone Vehicle/Train/YOLO11/F1_curve.png]

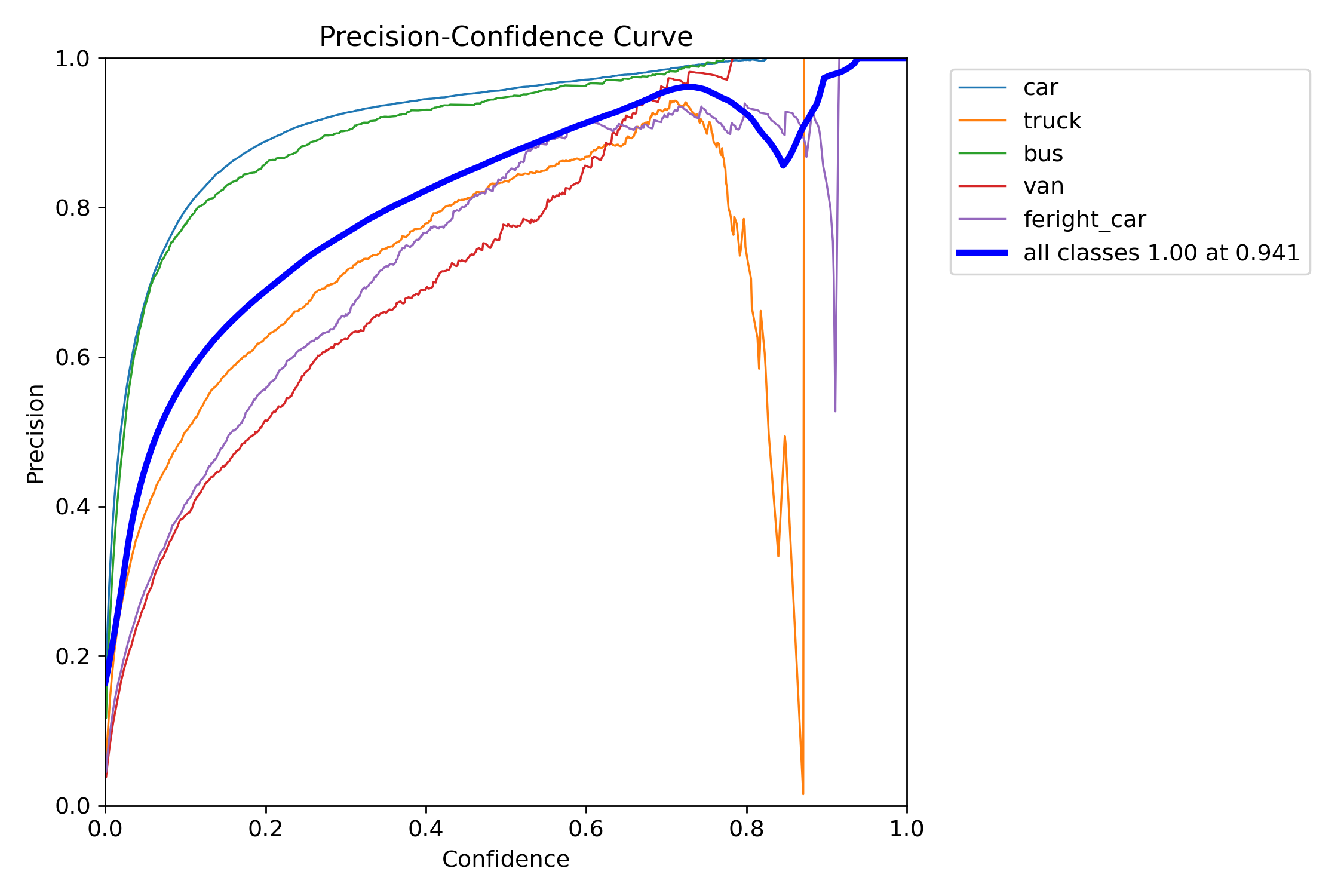

Supplement: S1 File — (ZIP) [file pone.0328248.s001.zip › S1 Model training result data/Drone Vehicle/Train/YOLO11/P_curve.png]

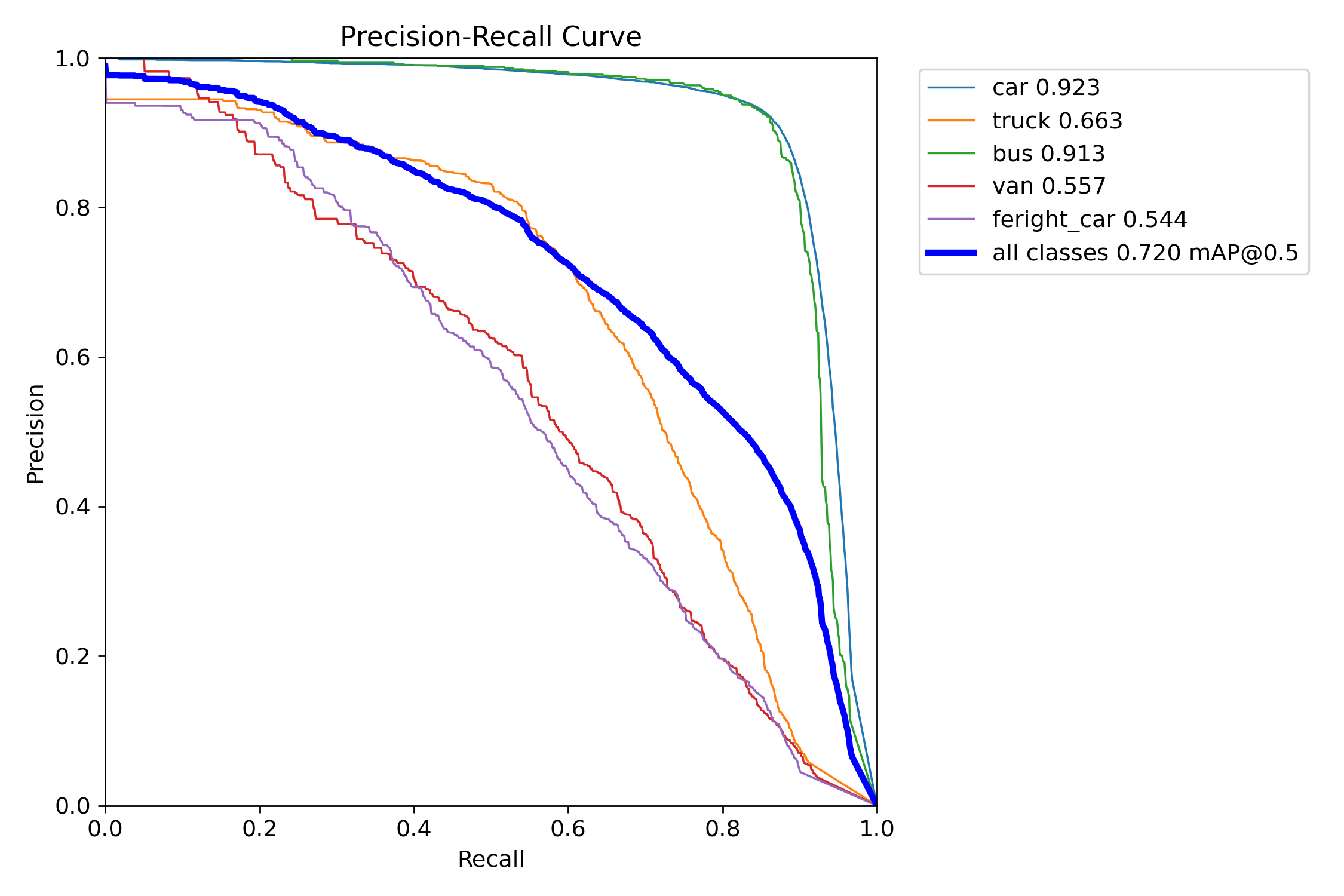

Supplement: S1 File — (ZIP) [file pone.0328248.s001.zip › S1 Model training result data/Drone Vehicle/Train/YOLO11/PR_curve.png]

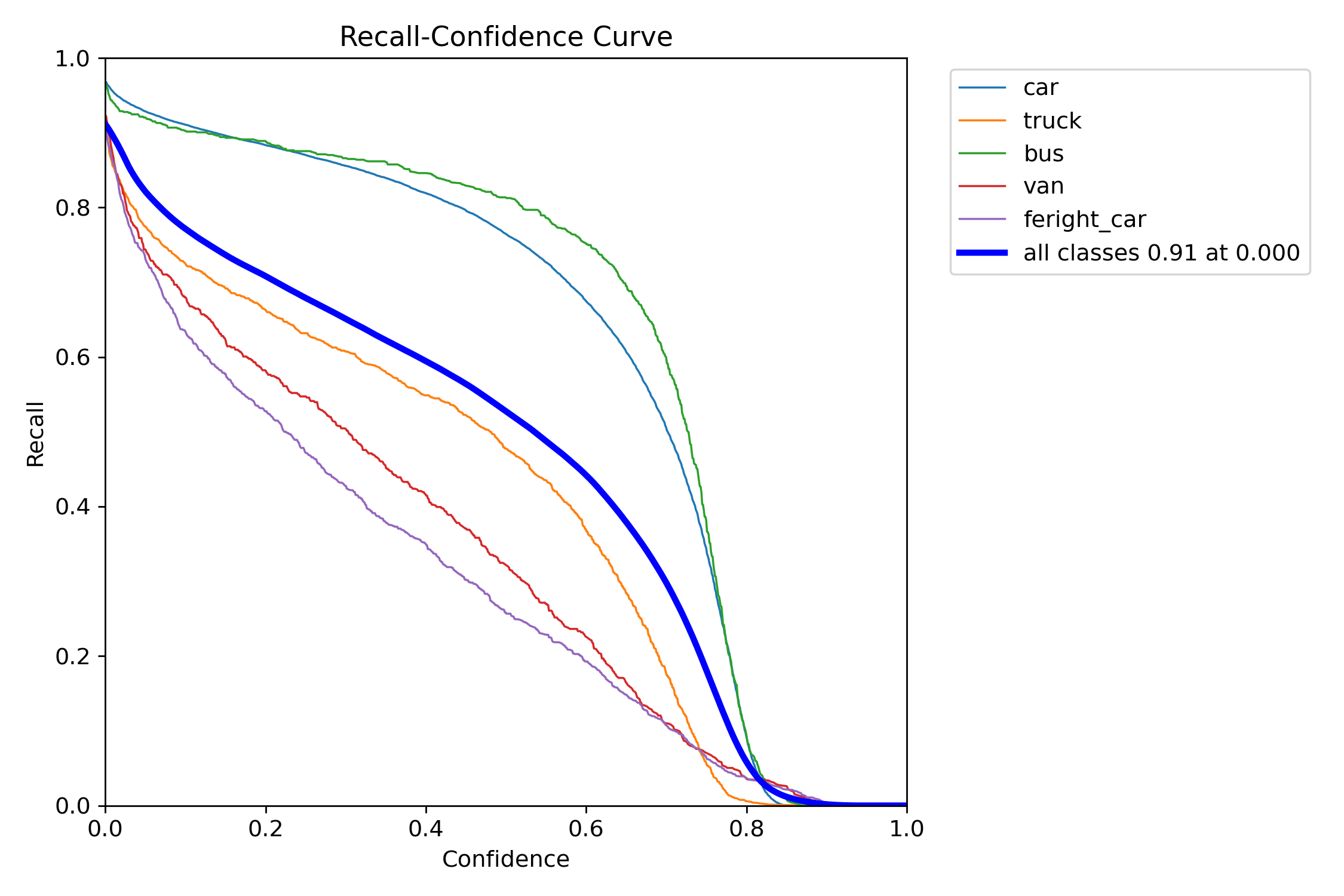

Supplement: S1 File — (ZIP) [file pone.0328248.s001.zip › S1 Model training result data/Drone Vehicle/Train/YOLO11/R_curve.png]

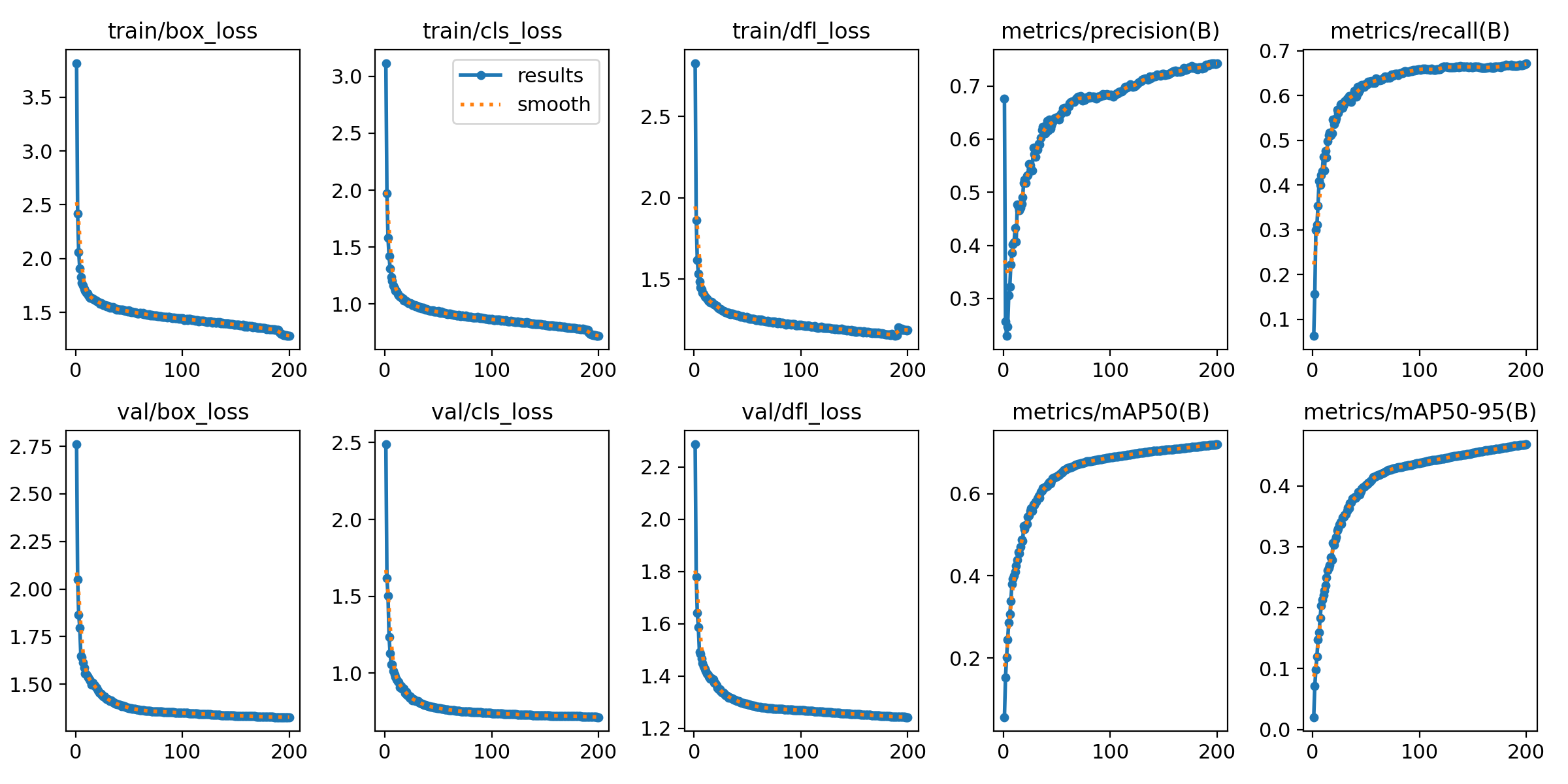

Supplement: S1 File — (ZIP) [file pone.0328248.s001.zip › S1 Model training result data/Drone Vehicle/Train/YOLO11/results.png]

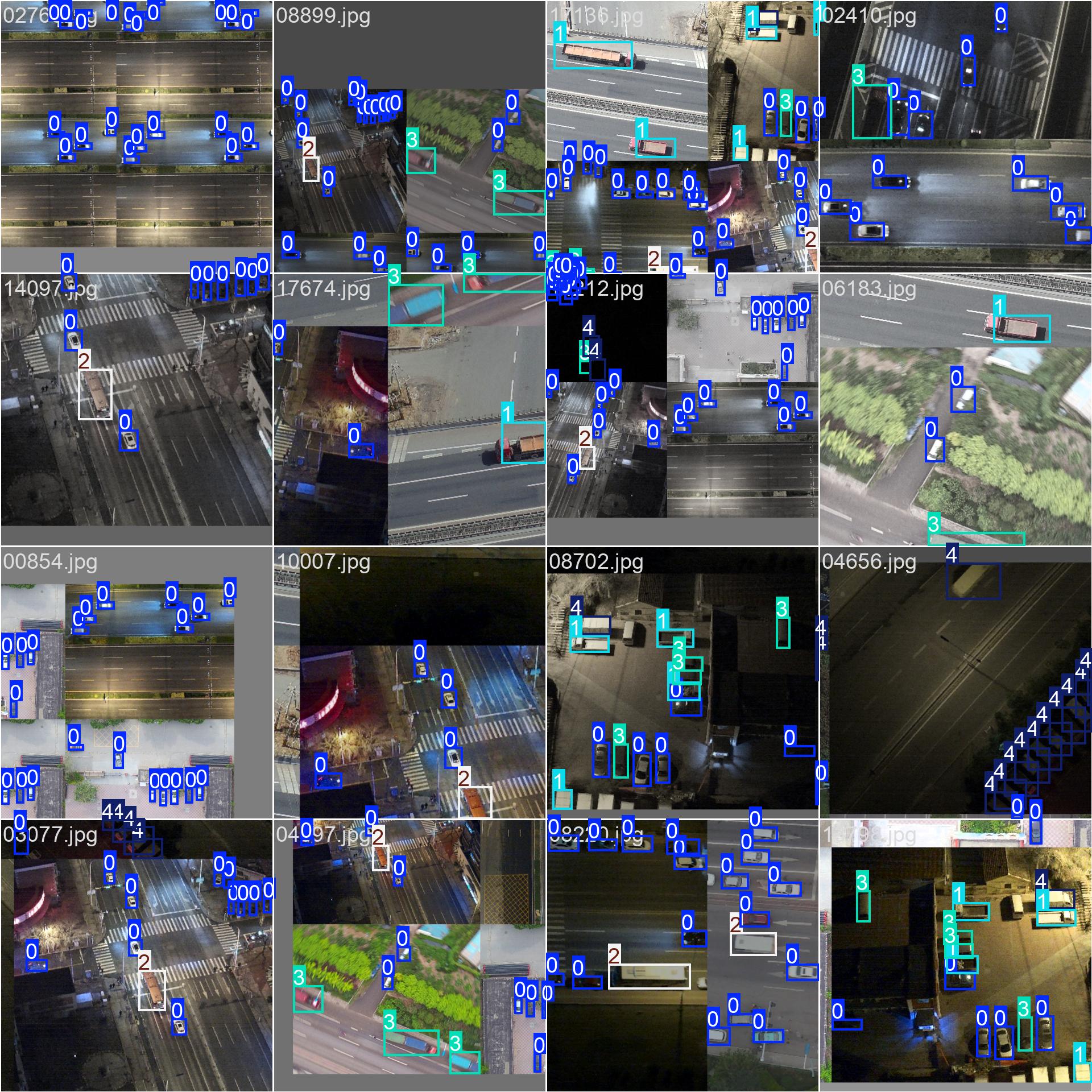

Supplement: S1 File — (ZIP) [file pone.0328248.s001.zip › S1 Model training result data/Drone Vehicle/Train/YOLO11/train_batch0.jpg]

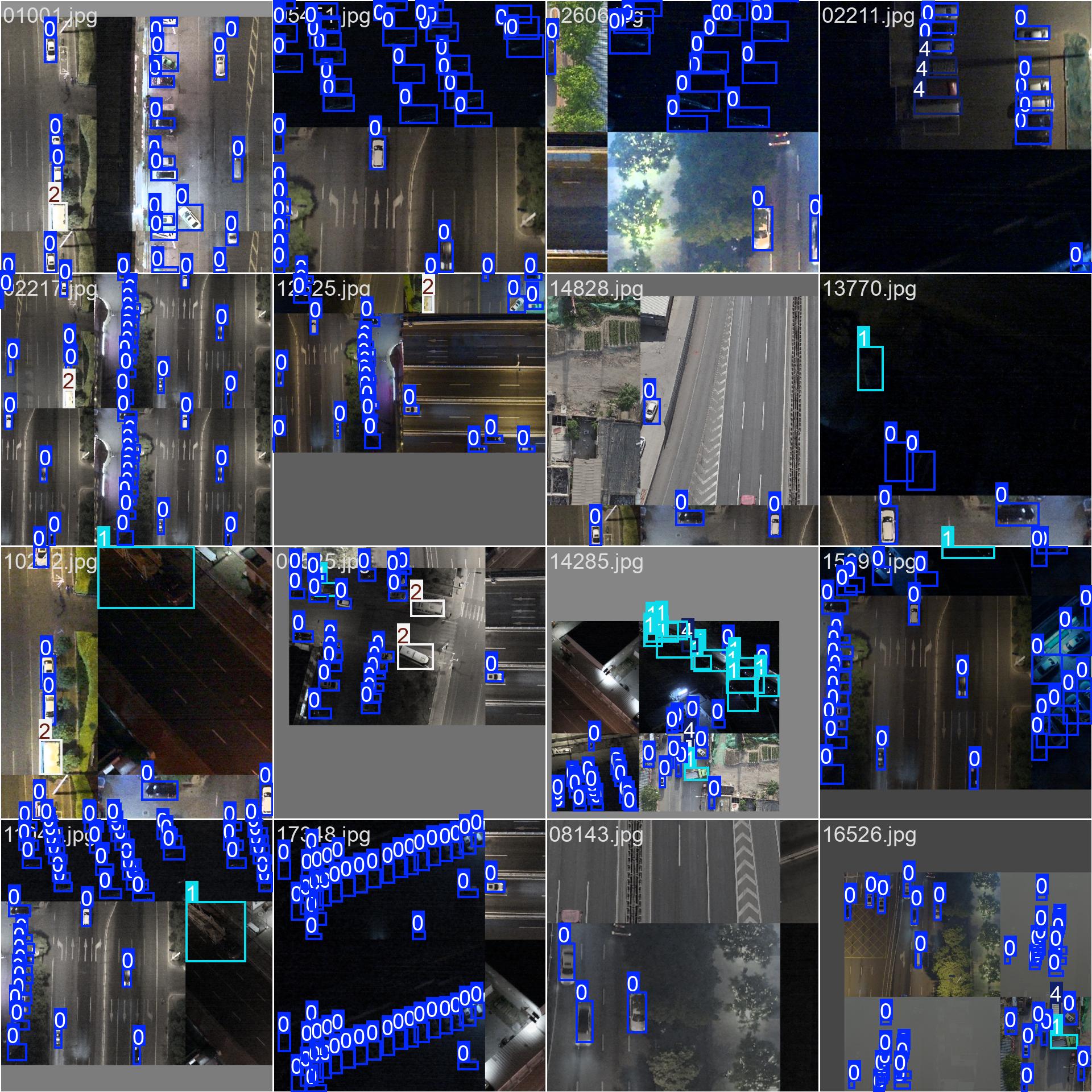

Supplement: S1 File — (ZIP) [file pone.0328248.s001.zip › S1 Model training result data/Drone Vehicle/Train/YOLO11/train_batch1.jpg]

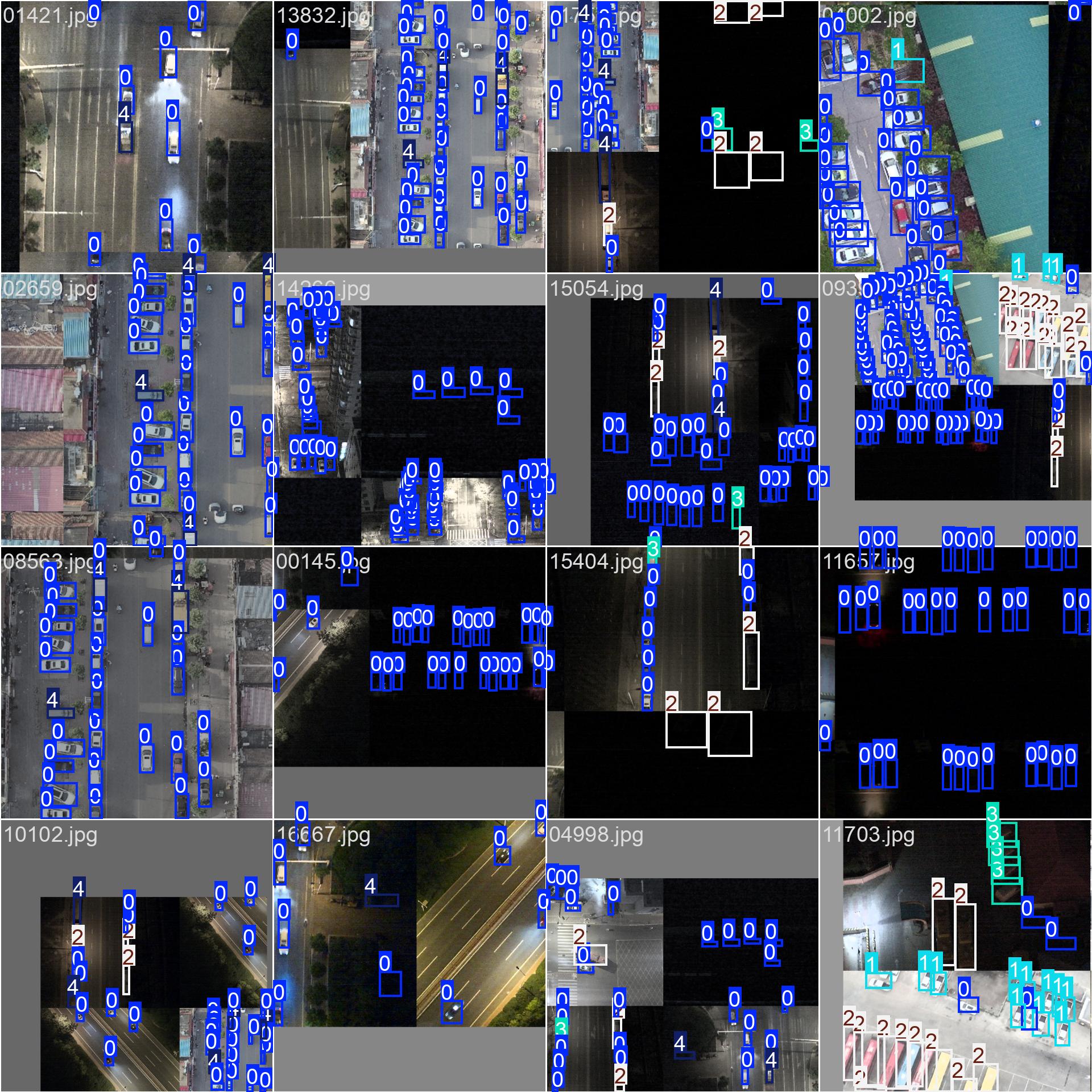

Supplement: S1 File — (ZIP) [file pone.0328248.s001.zip › S1 Model training result data/Drone Vehicle/Train/YOLO11/train_batch2.jpg]

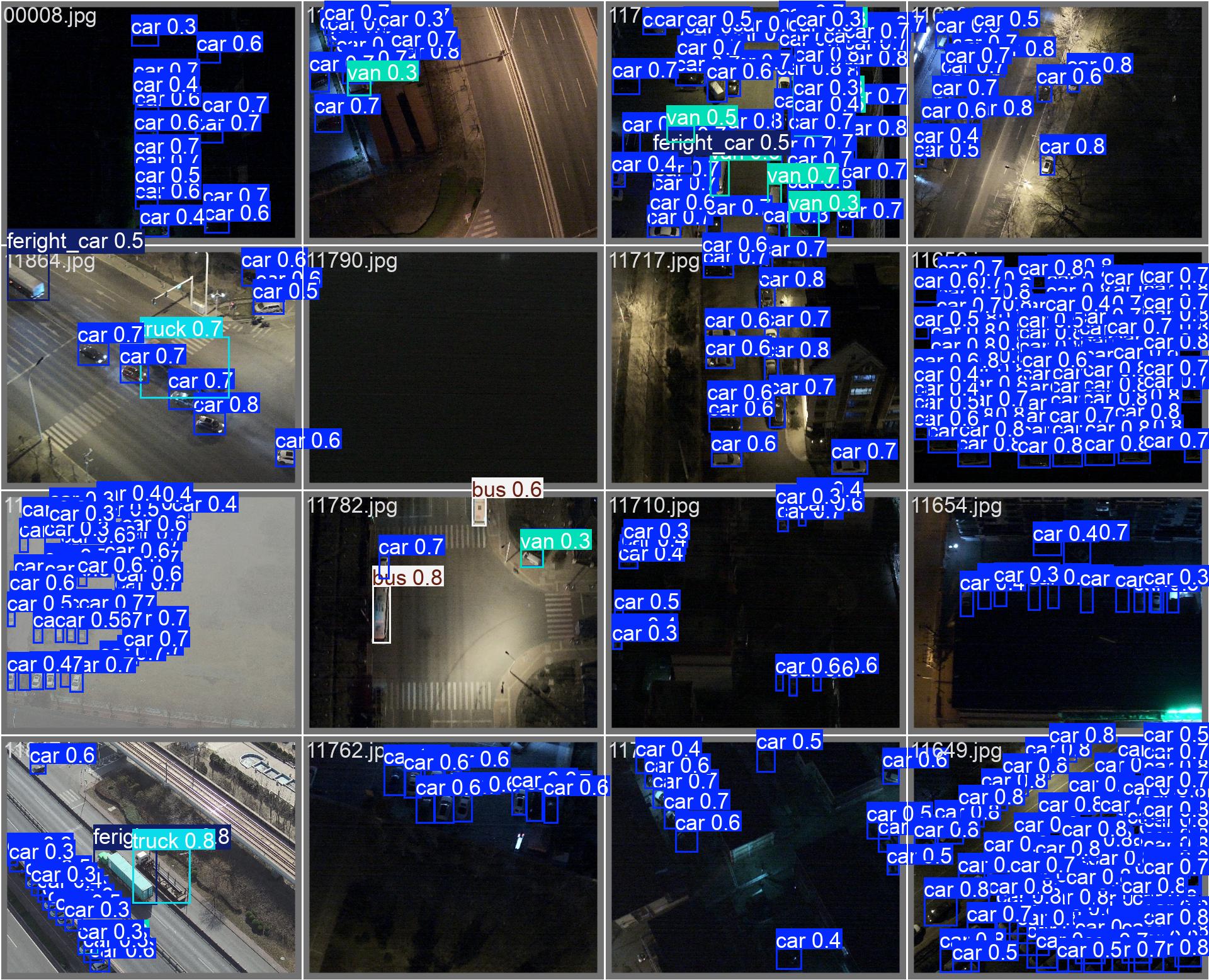

Supplement: S1 File — (ZIP) [file pone.0328248.s001.zip › S1 Model training result data/Drone Vehicle/Train/YOLO11/val_batch0_pred.jpg]

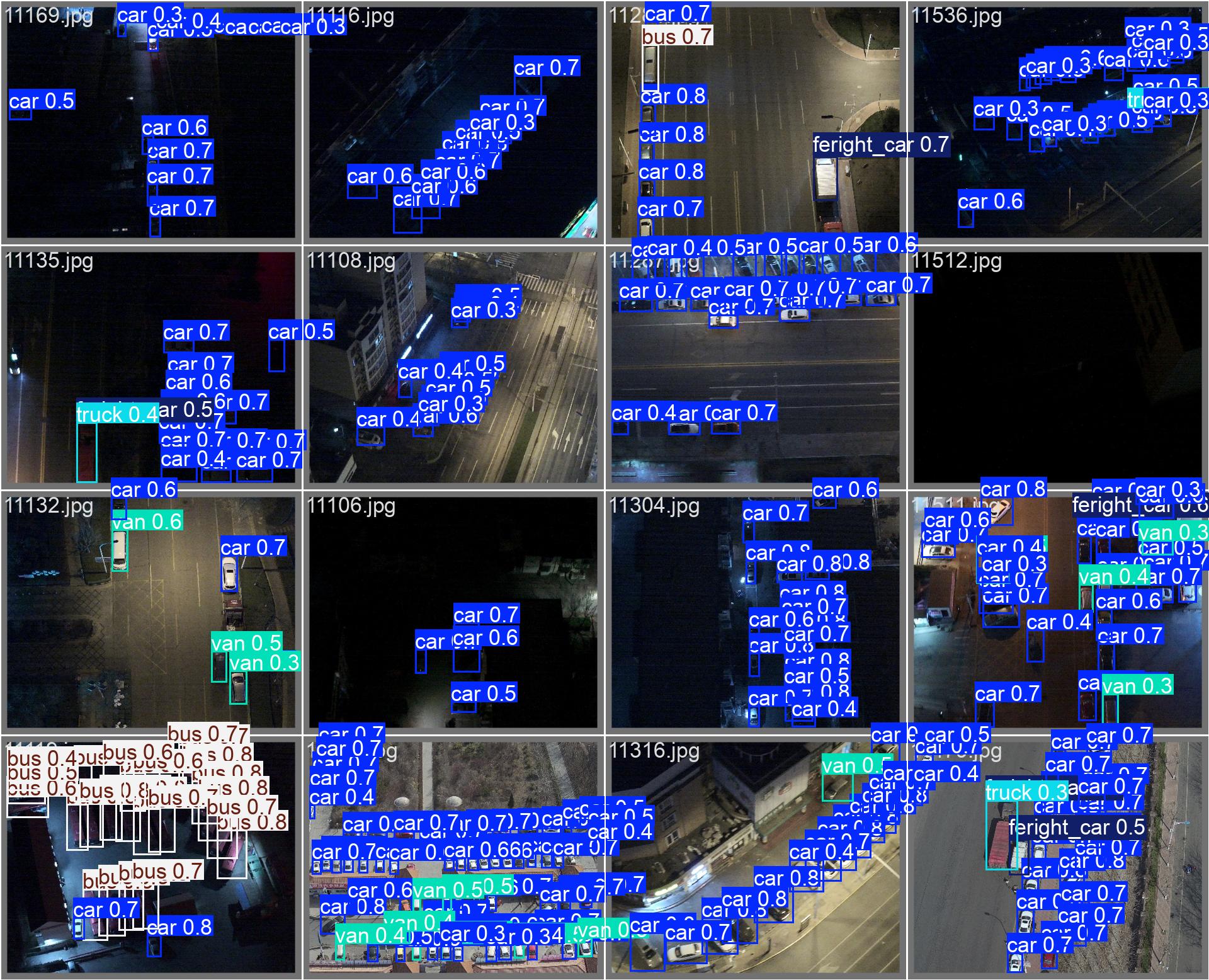

Supplement: S1 File — (ZIP) [file pone.0328248.s001.zip › S1 Model training result data/Drone Vehicle/Train/YOLO11/val_batch1_pred.jpg]

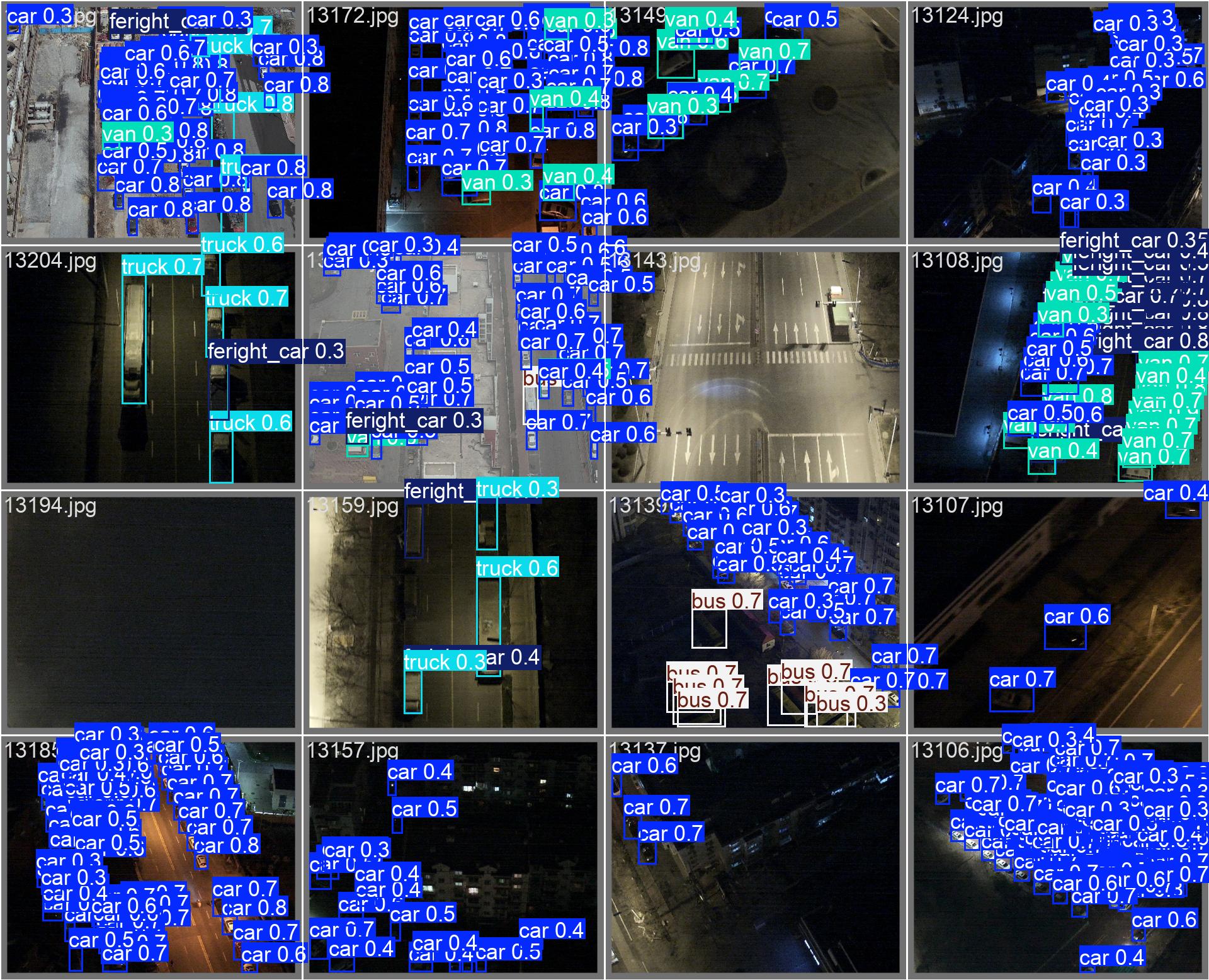

Supplement: S1 File — (ZIP) [file pone.0328248.s001.zip › S1 Model training result data/Drone Vehicle/Train/YOLO11/val_batch2_pred.jpg]

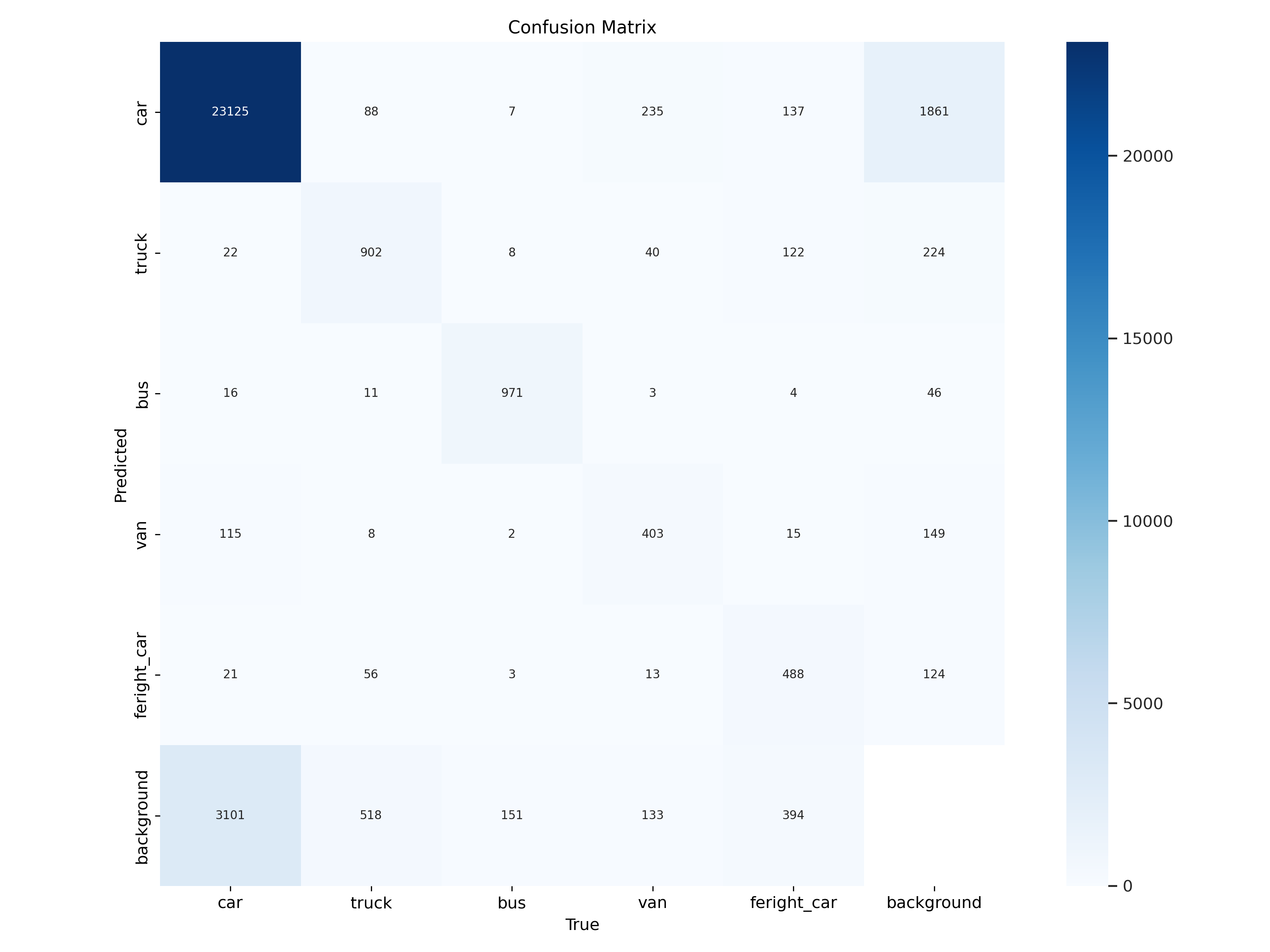

Supplement: S1 File — (ZIP) [file pone.0328248.s001.zip › S1 Model training result data/FPS/Drone Vehicle/LMAD-YOLO11/confusion_matrix.png]

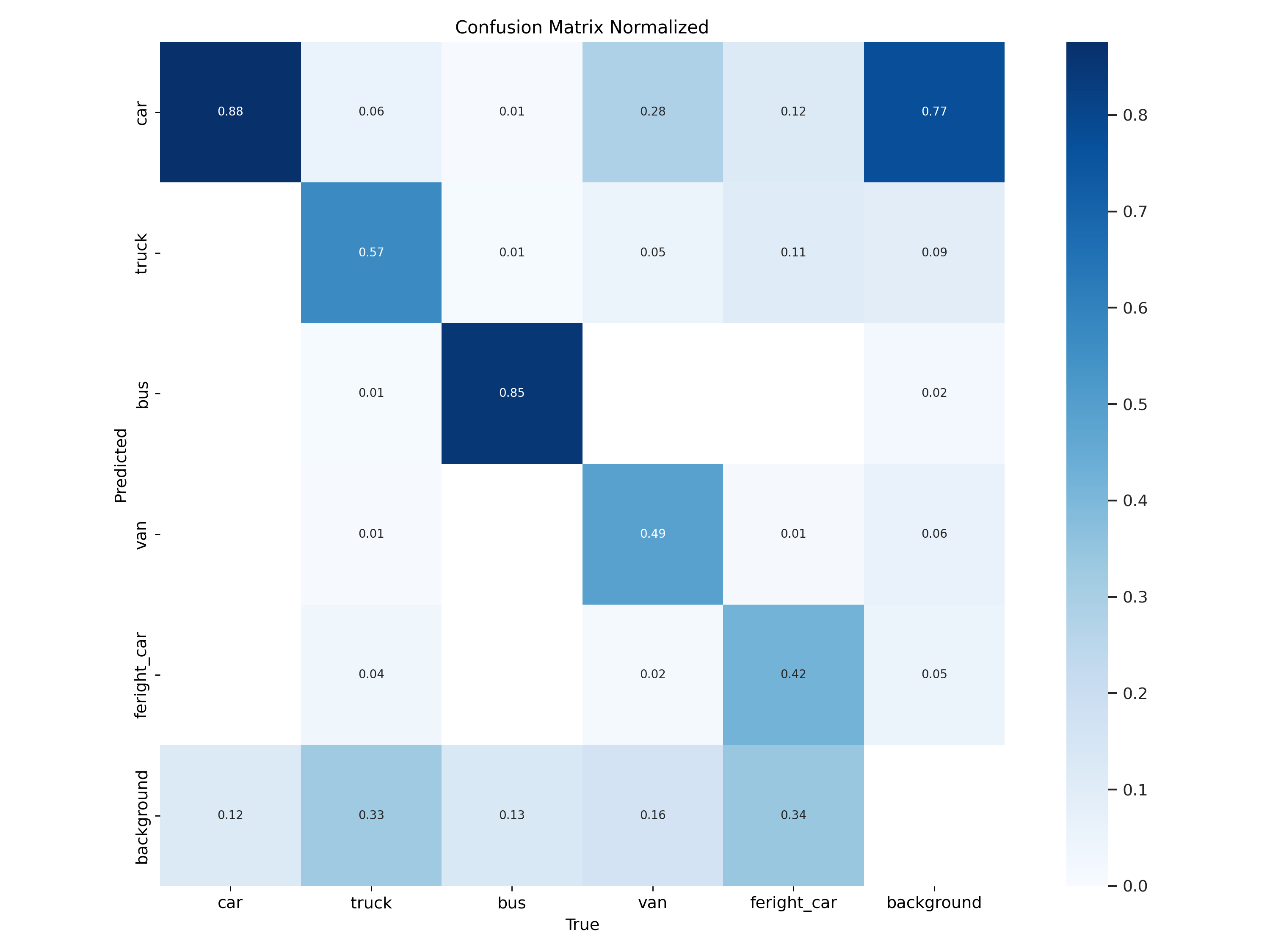

Supplement: S1 File — (ZIP) [file pone.0328248.s001.zip › S1 Model training result data/FPS/Drone Vehicle/LMAD-YOLO11/confusion_matrix_normalized.png]

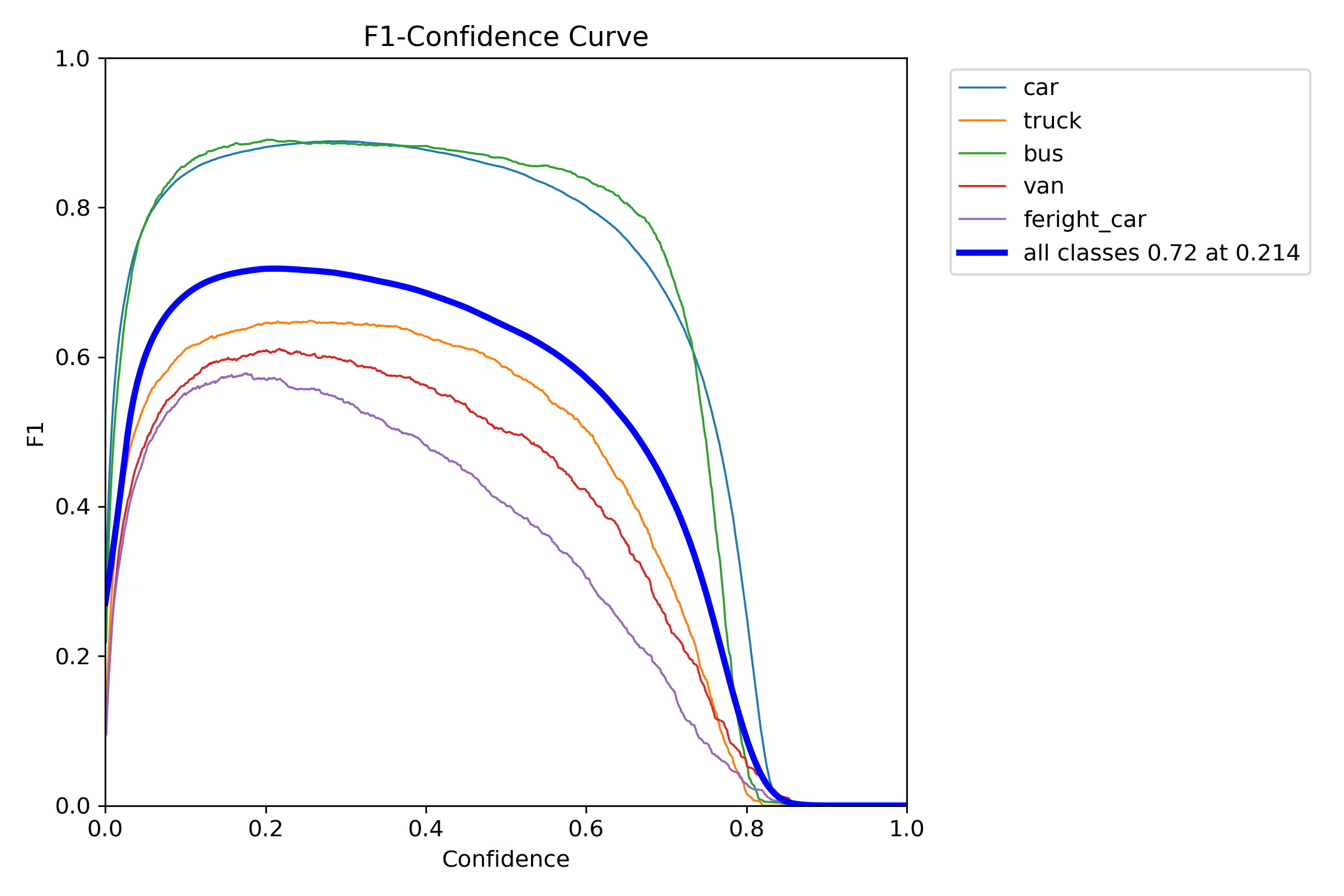

Supplement: S1 File — (ZIP) [file pone.0328248.s001.zip › S1 Model training result data/FPS/Drone Vehicle/LMAD-YOLO11/F1_curve.png]

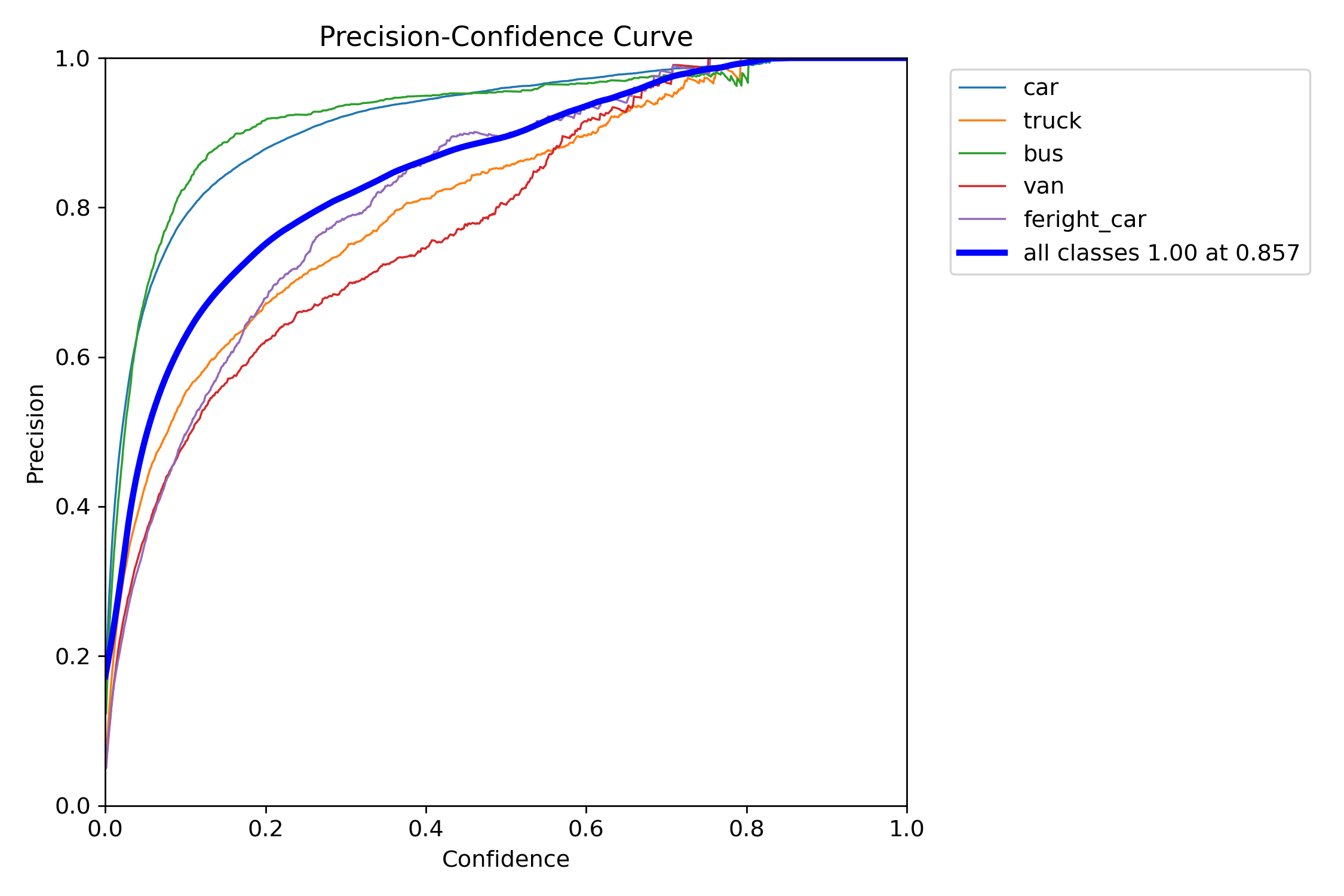

Supplement: S1 File — (ZIP) [file pone.0328248.s001.zip › S1 Model training result data/FPS/Drone Vehicle/LMAD-YOLO11/P_curve.png]

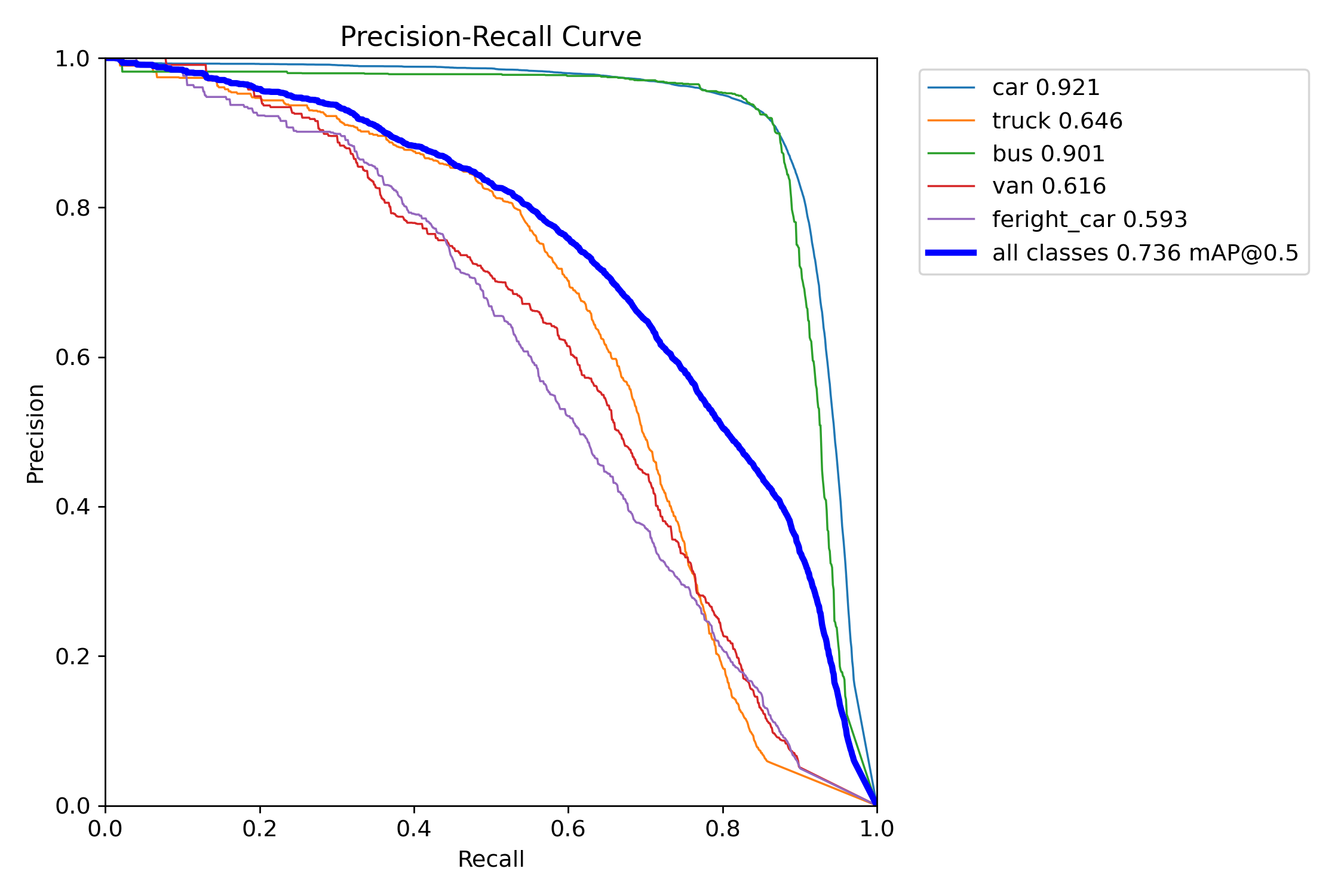

Supplement: S1 File — (ZIP) [file pone.0328248.s001.zip › S1 Model training result data/FPS/Drone Vehicle/LMAD-YOLO11/PR_curve.png]

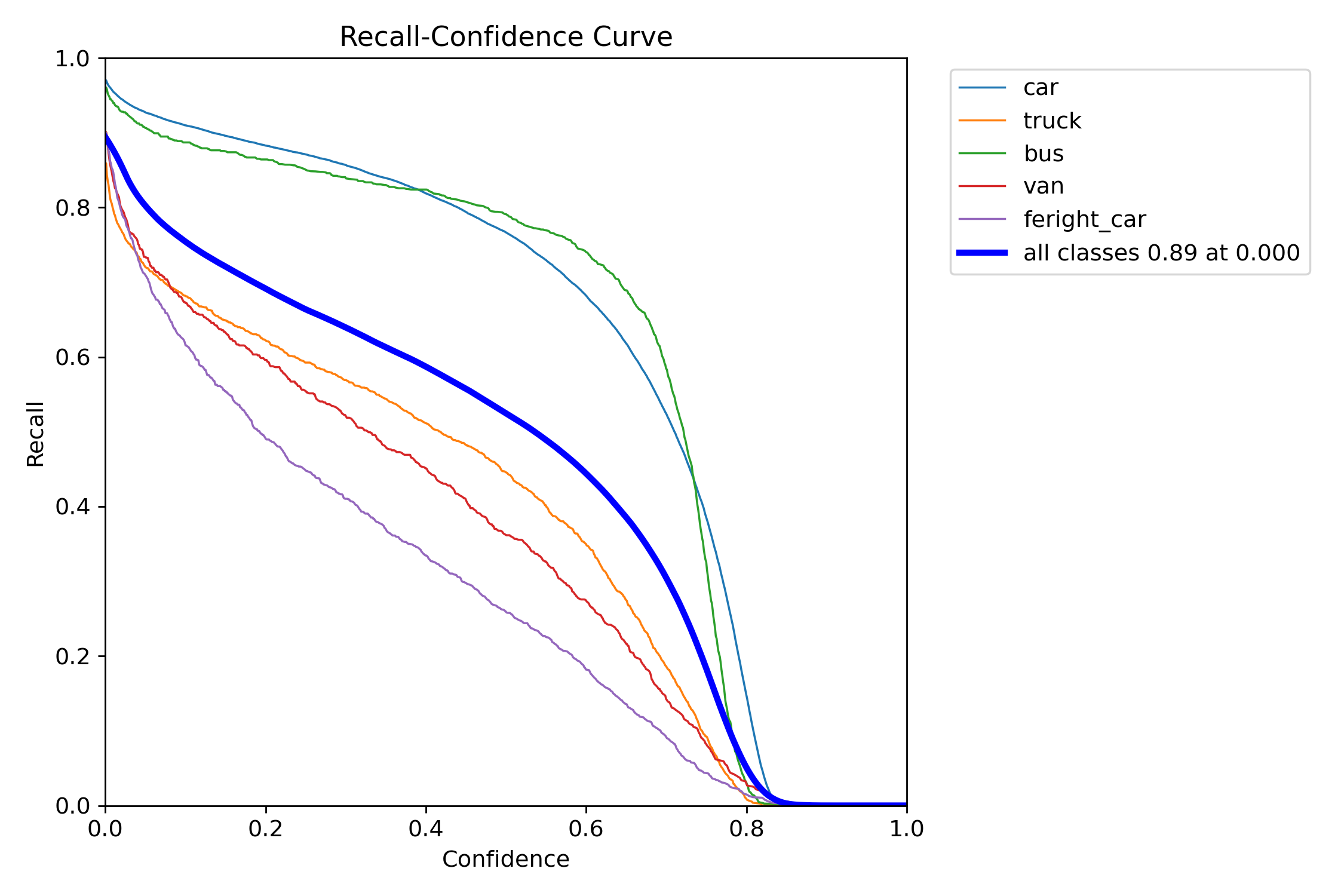

Supplement: S1 File — (ZIP) [file pone.0328248.s001.zip › S1 Model training result data/FPS/Drone Vehicle/LMAD-YOLO11/R_curve.png]

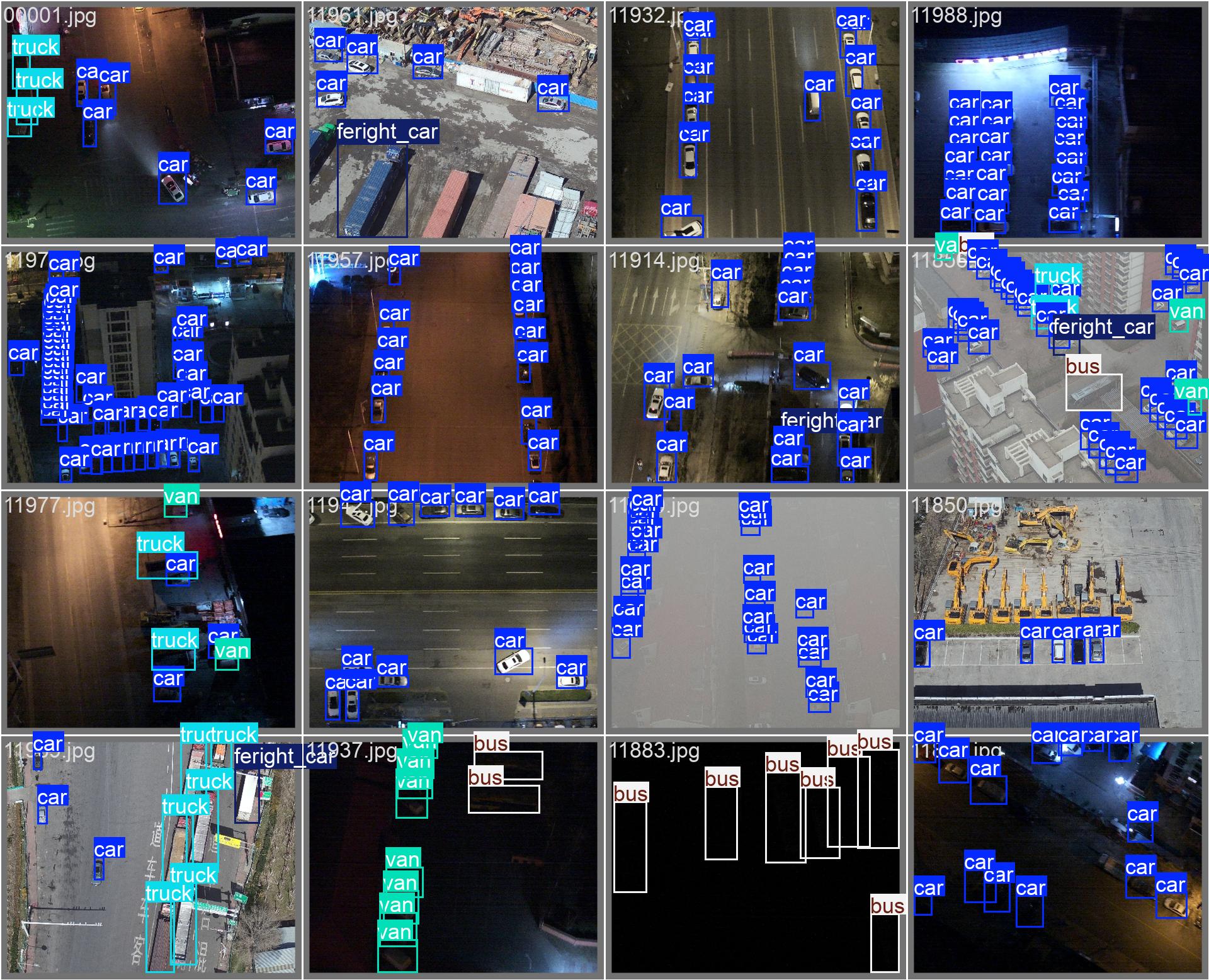

Supplement: S1 File — (ZIP) [file pone.0328248.s001.zip › S1 Model training result data/FPS/Drone Vehicle/LMAD-YOLO11/val_batch0_labels.jpg]

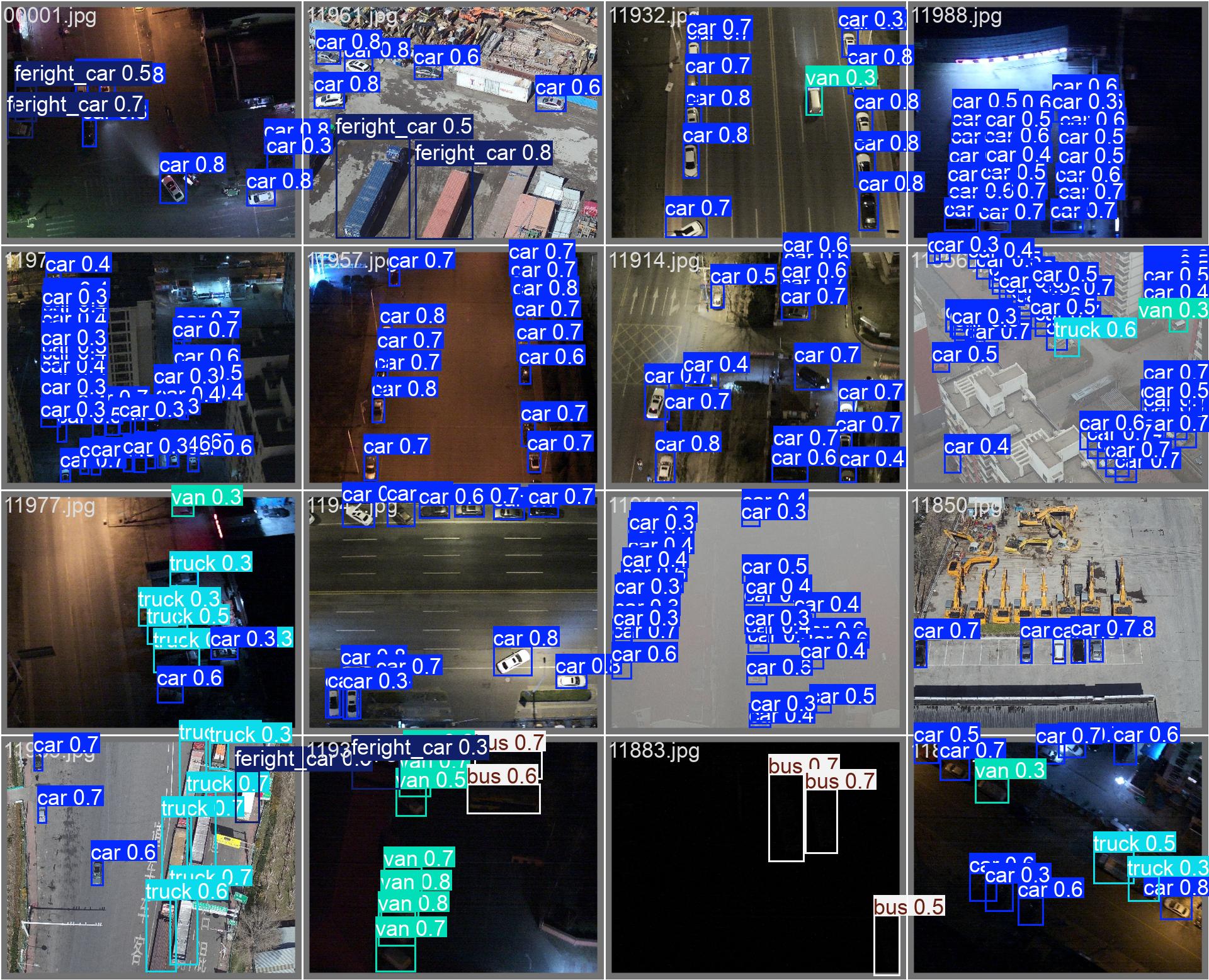

Supplement: S1 File — (ZIP) [file pone.0328248.s001.zip › S1 Model training result data/FPS/Drone Vehicle/LMAD-YOLO11/val_batch0_pred.jpg]

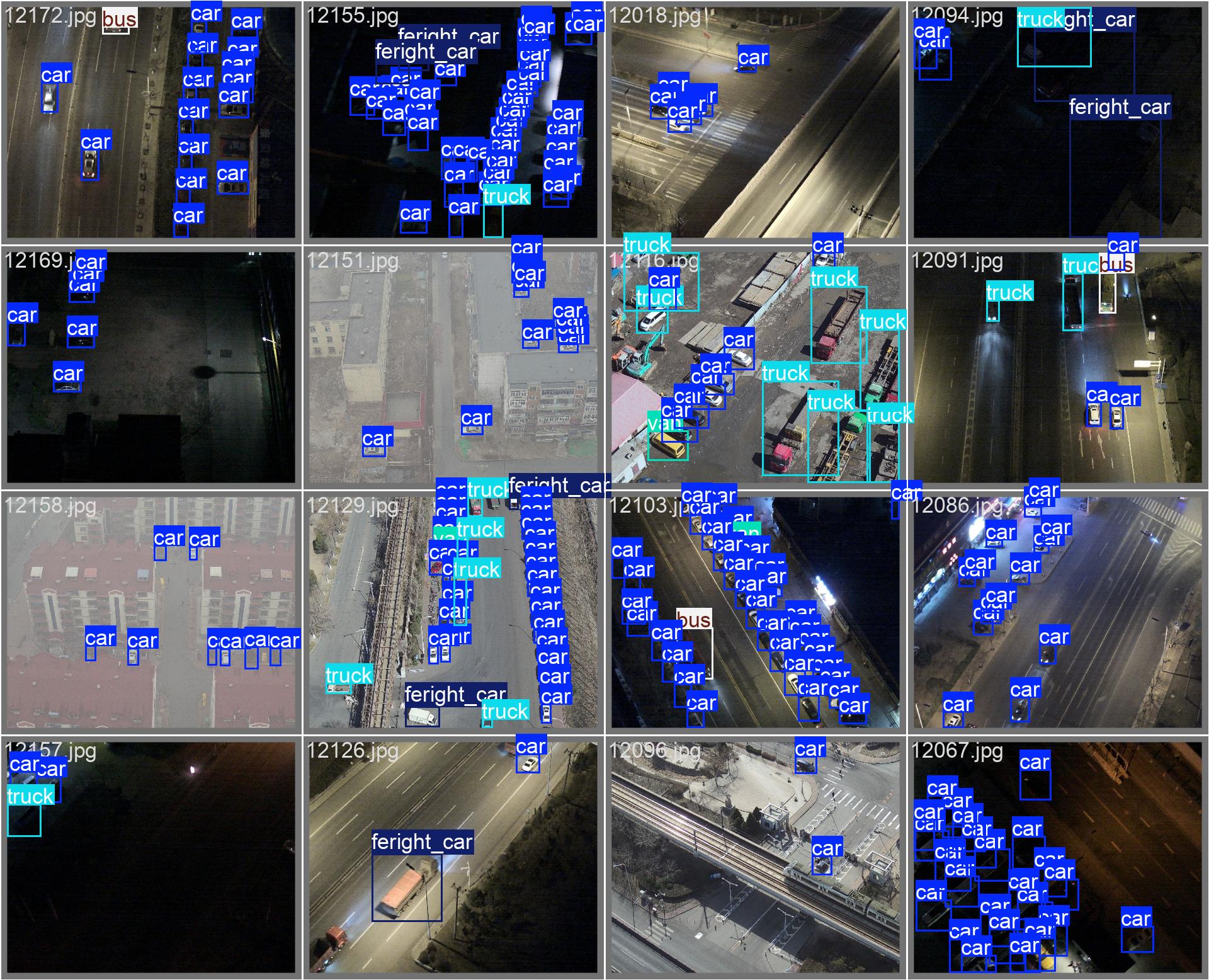

Supplement: S1 File — (ZIP) [file pone.0328248.s001.zip › S1 Model training result data/FPS/Drone Vehicle/LMAD-YOLO11/val_batch1_labels.jpg]

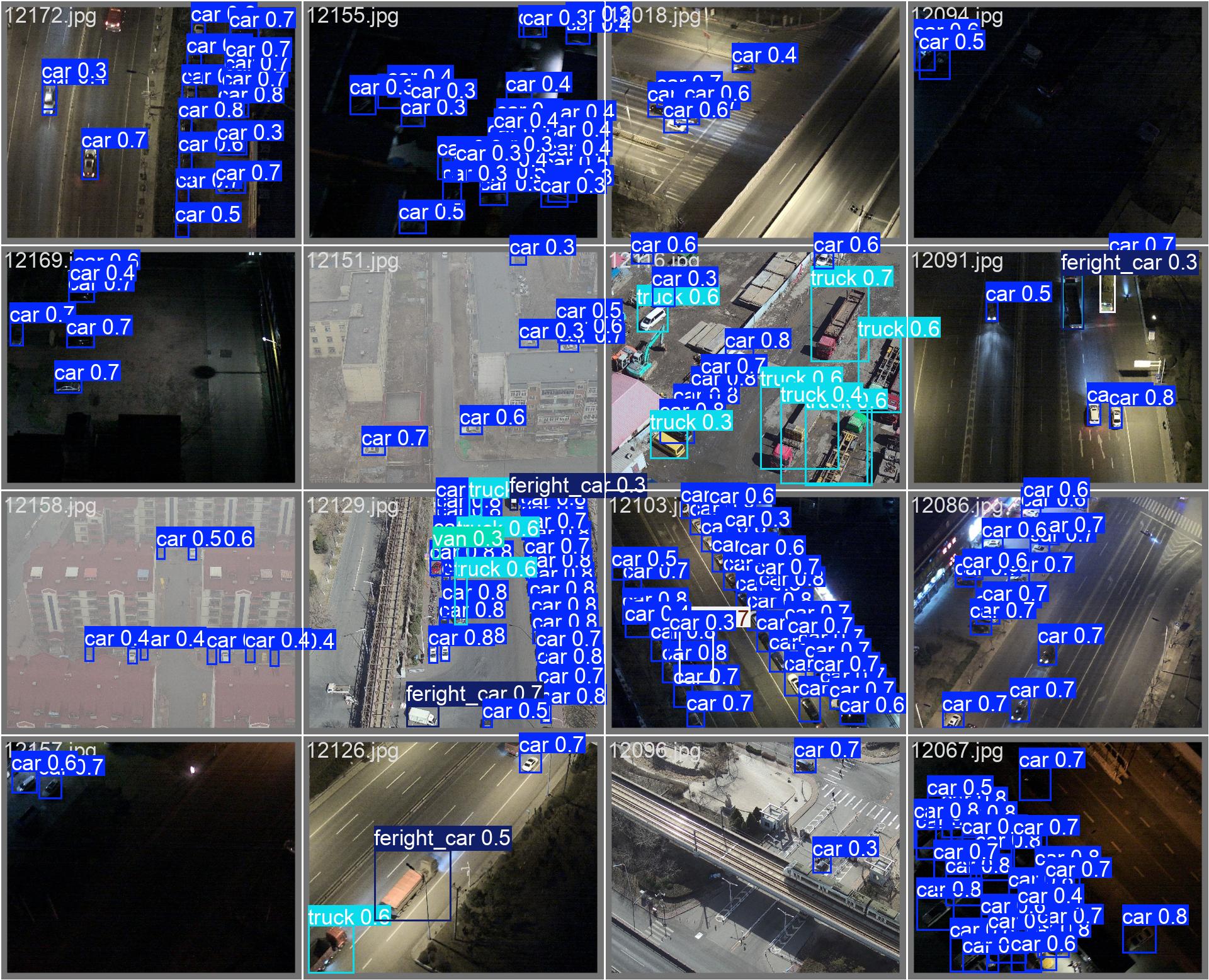

Supplement: S1 File — (ZIP) [file pone.0328248.s001.zip › S1 Model training result data/FPS/Drone Vehicle/LMAD-YOLO11/val_batch1_pred.jpg]

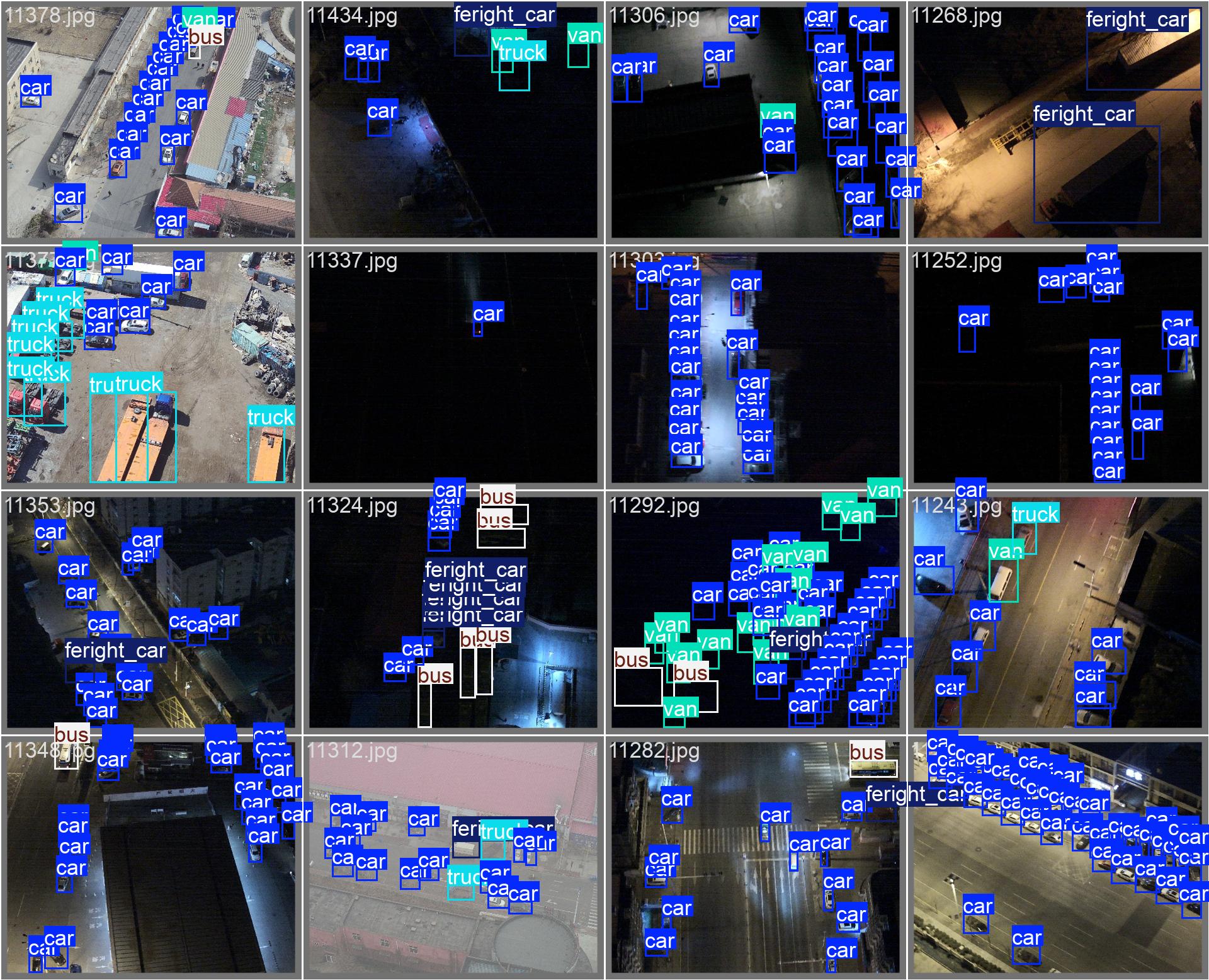

Supplement: S1 File — (ZIP) [file pone.0328248.s001.zip › S1 Model training result data/FPS/Drone Vehicle/LMAD-YOLO11/val_batch2_labels.jpg]

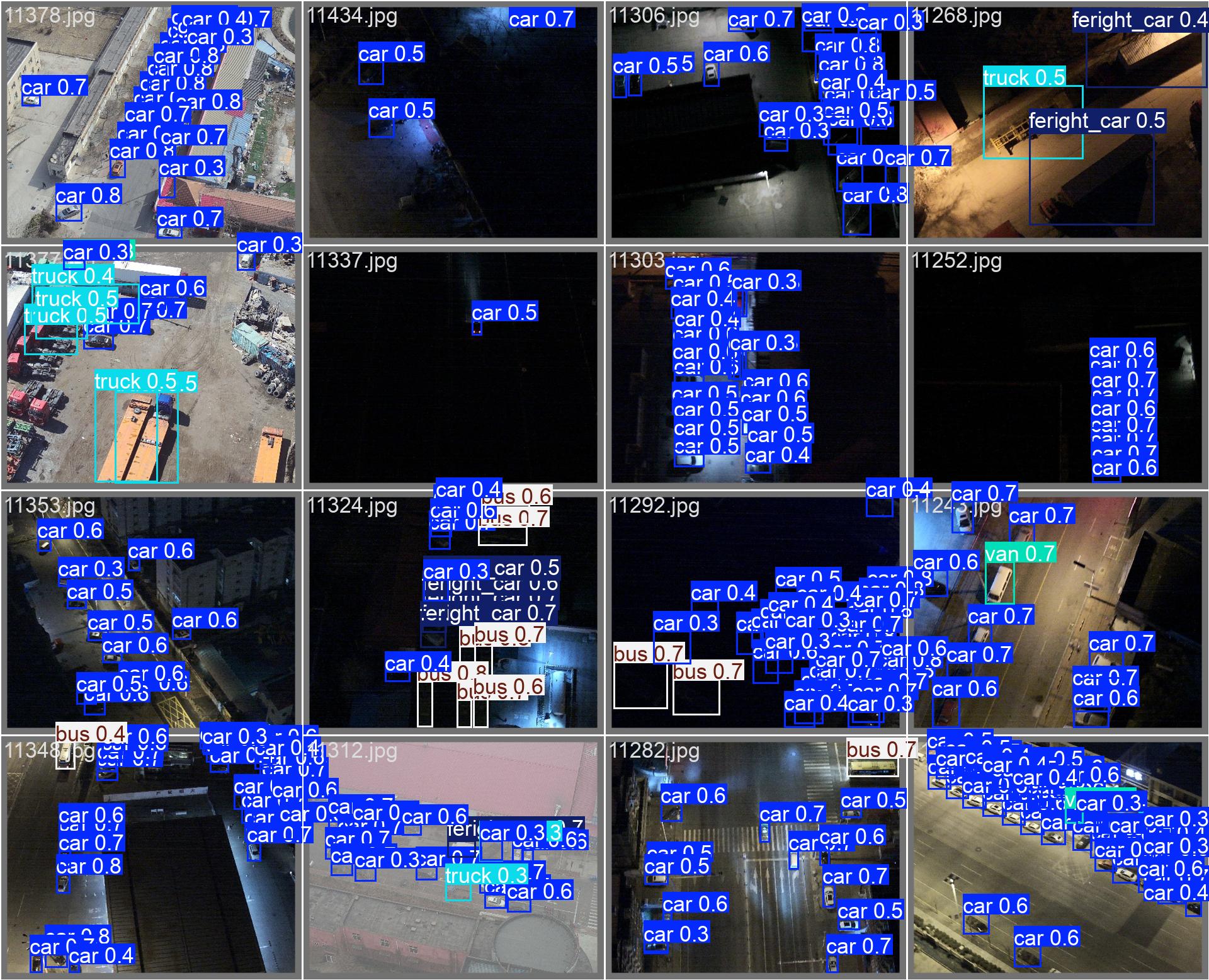

Supplement: S1 File — (ZIP) [file pone.0328248.s001.zip › S1 Model training result data/FPS/Drone Vehicle/LMAD-YOLO11/val_batch2_pred.jpg]

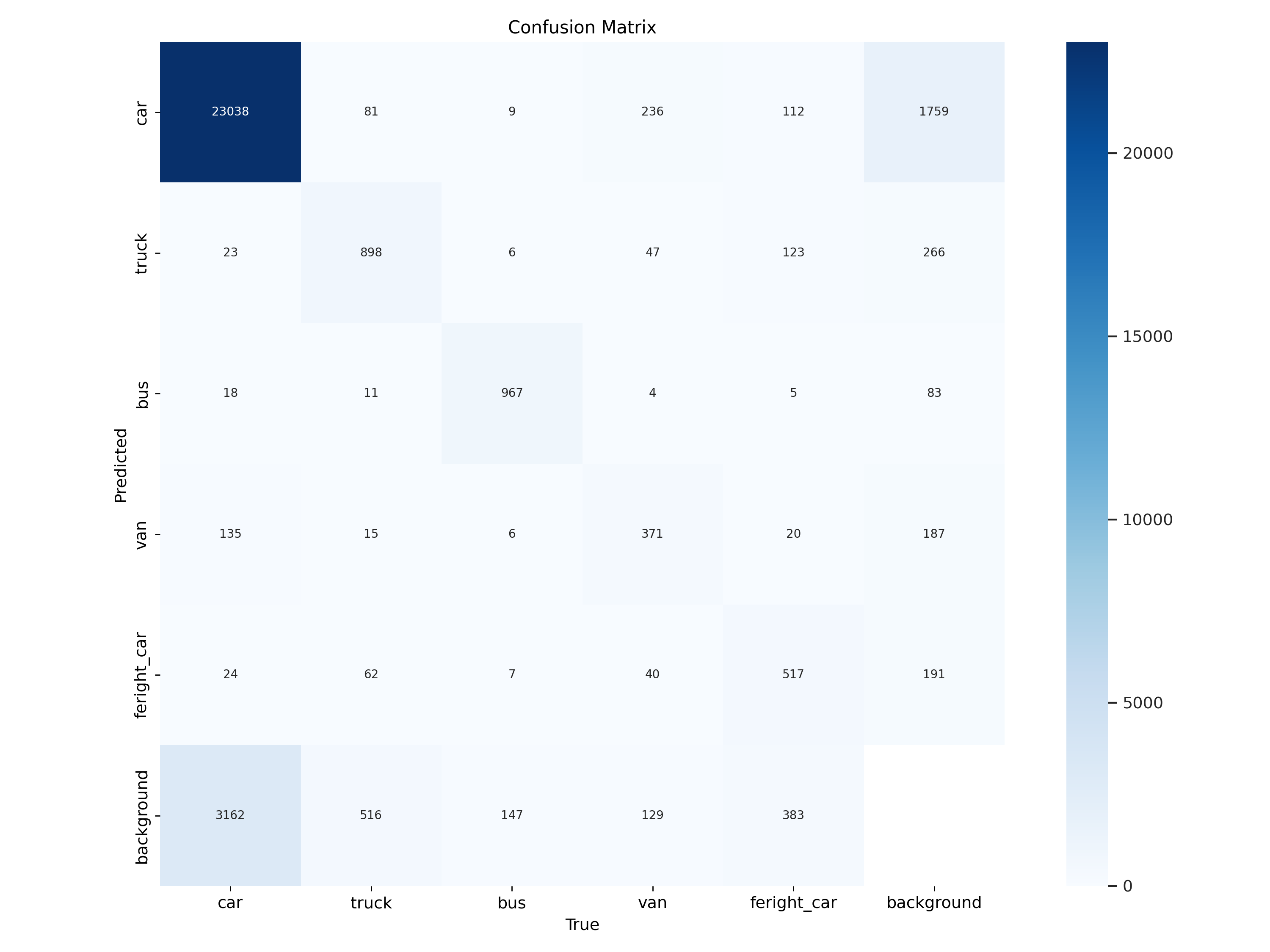

Supplement: S1 File — (ZIP) [file pone.0328248.s001.zip › S1 Model training result data/FPS/Drone Vehicle/YOLO11/confusion_matrix.png]

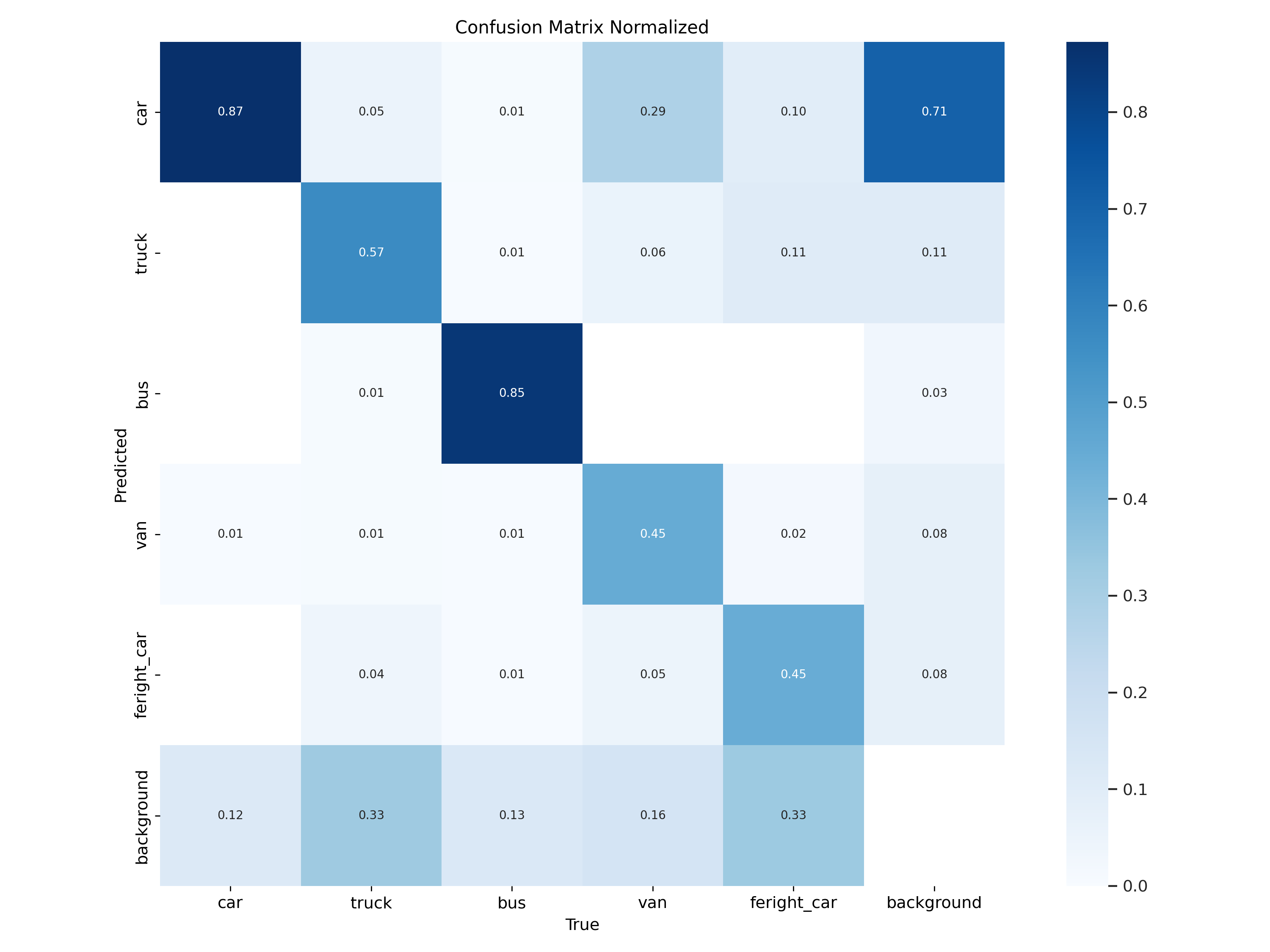

Supplement: S1 File — (ZIP) [file pone.0328248.s001.zip › S1 Model training result data/FPS/Drone Vehicle/YOLO11/confusion_matrix_normalized.png]

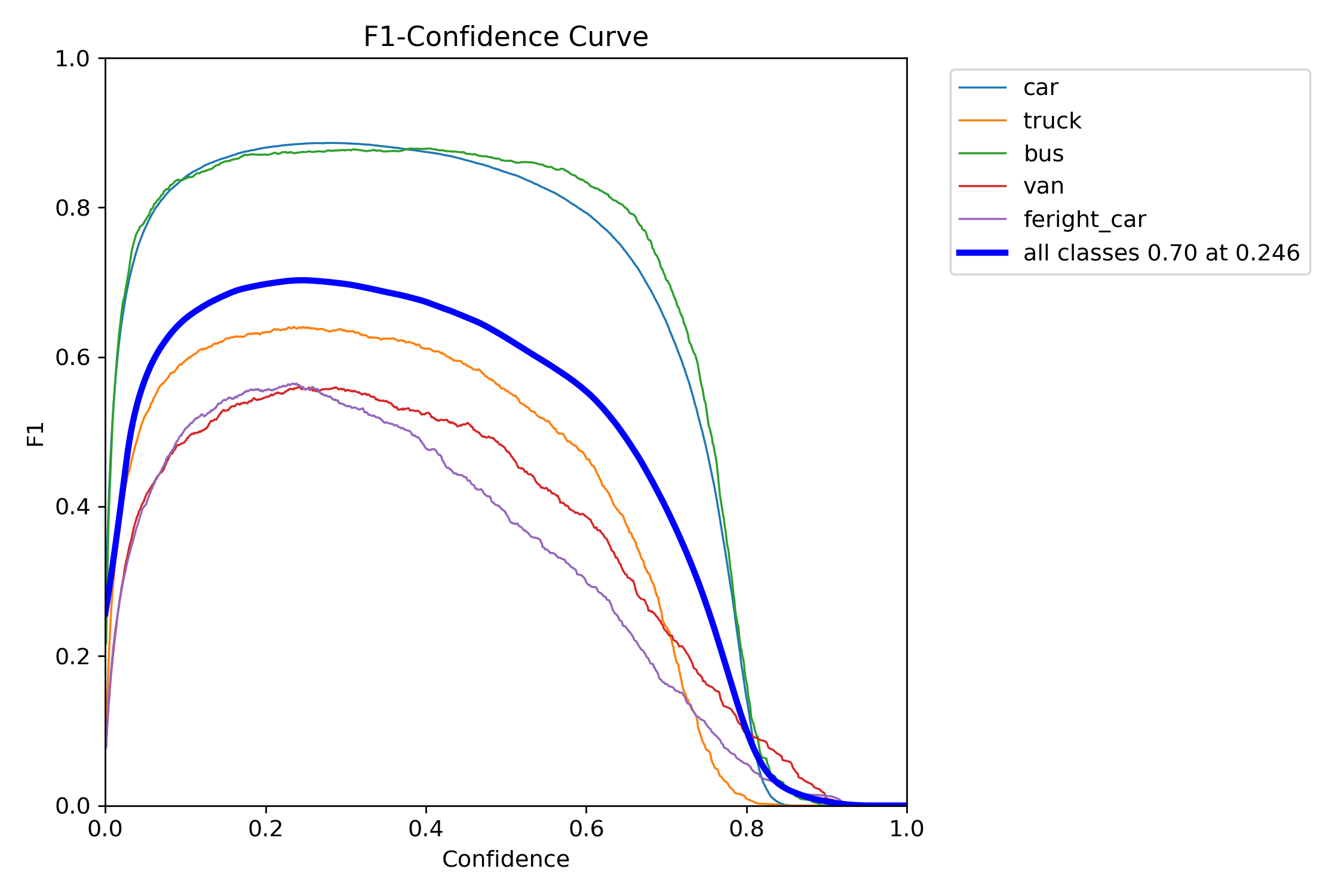

Supplement: S1 File — (ZIP) [file pone.0328248.s001.zip › S1 Model training result data/FPS/Drone Vehicle/YOLO11/F1_curve.png]

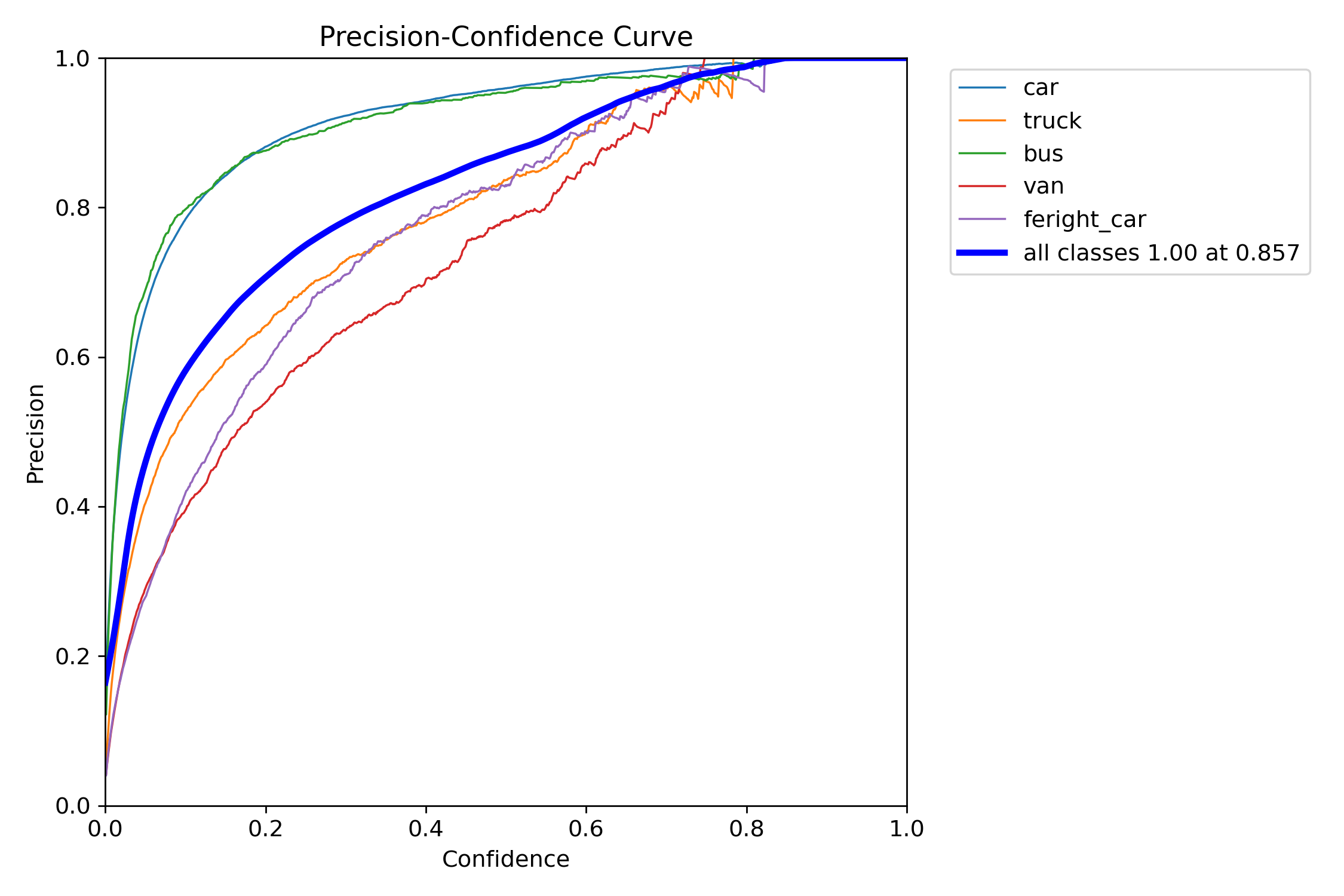

Supplement: S1 File — (ZIP) [file pone.0328248.s001.zip › S1 Model training result data/FPS/Drone Vehicle/YOLO11/P_curve.png]

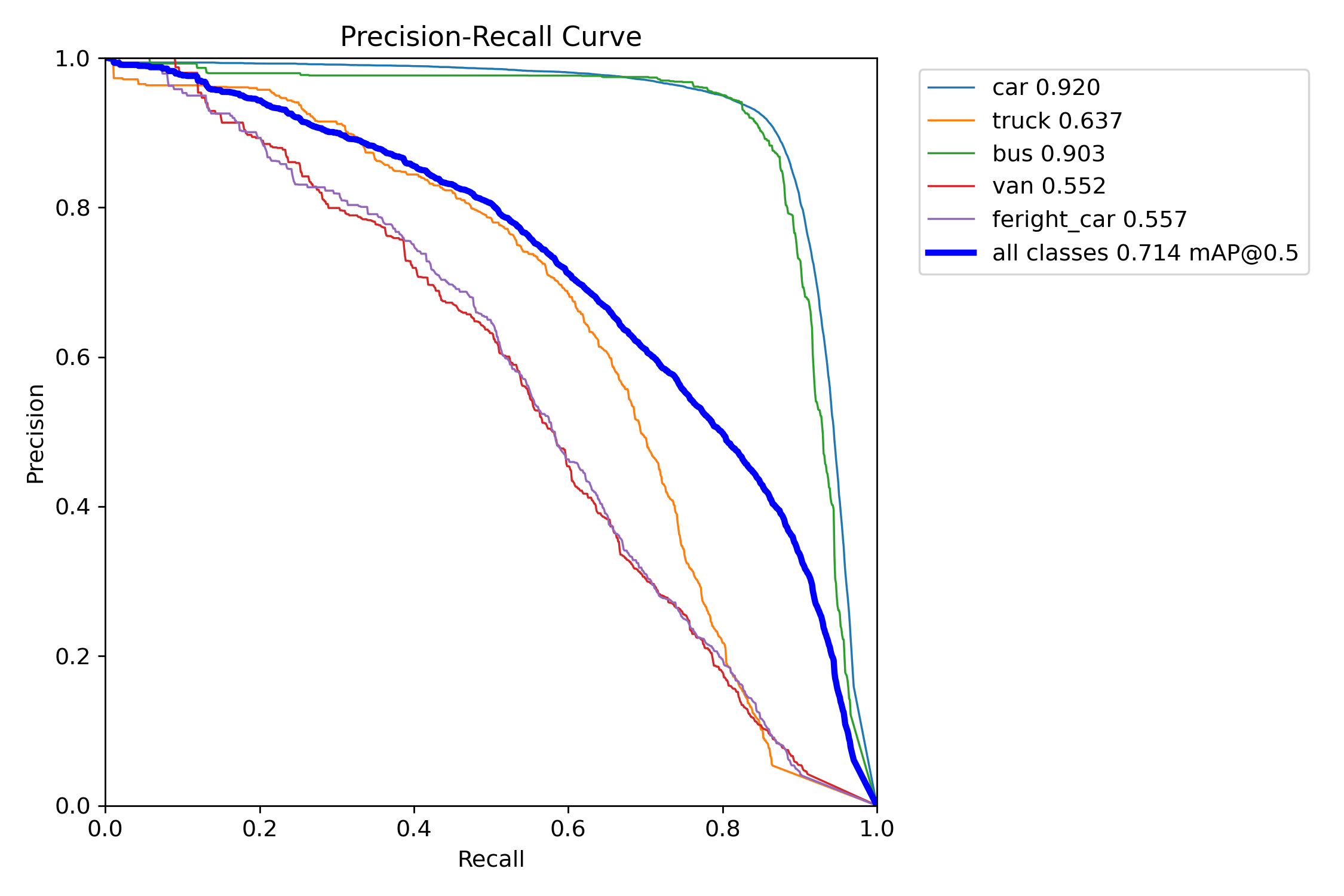

Supplement: S1 File — (ZIP) [file pone.0328248.s001.zip › S1 Model training result data/FPS/Drone Vehicle/YOLO11/PR_curve.png]

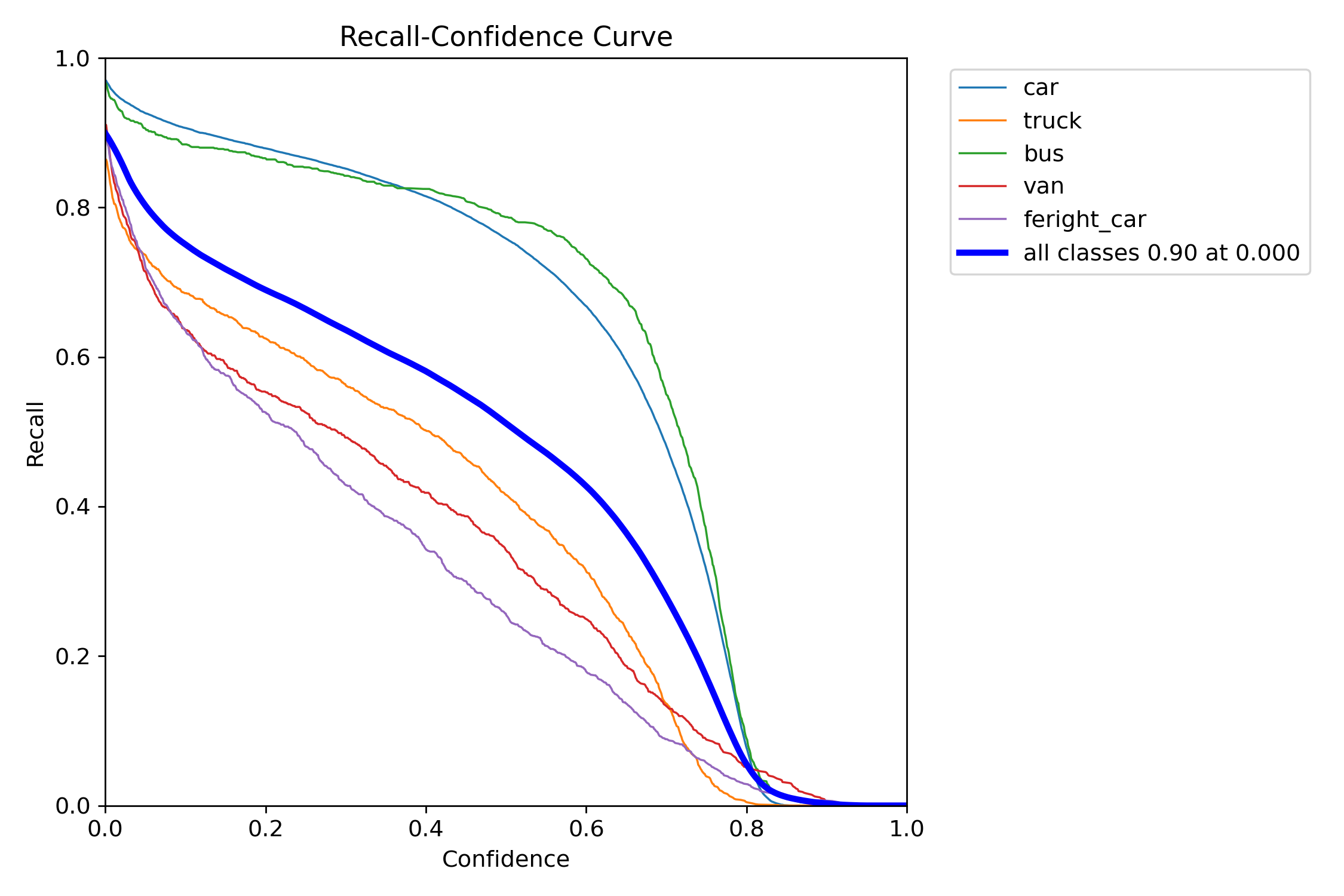

Supplement: S1 File — (ZIP) [file pone.0328248.s001.zip › S1 Model training result data/FPS/Drone Vehicle/YOLO11/R_curve.png]

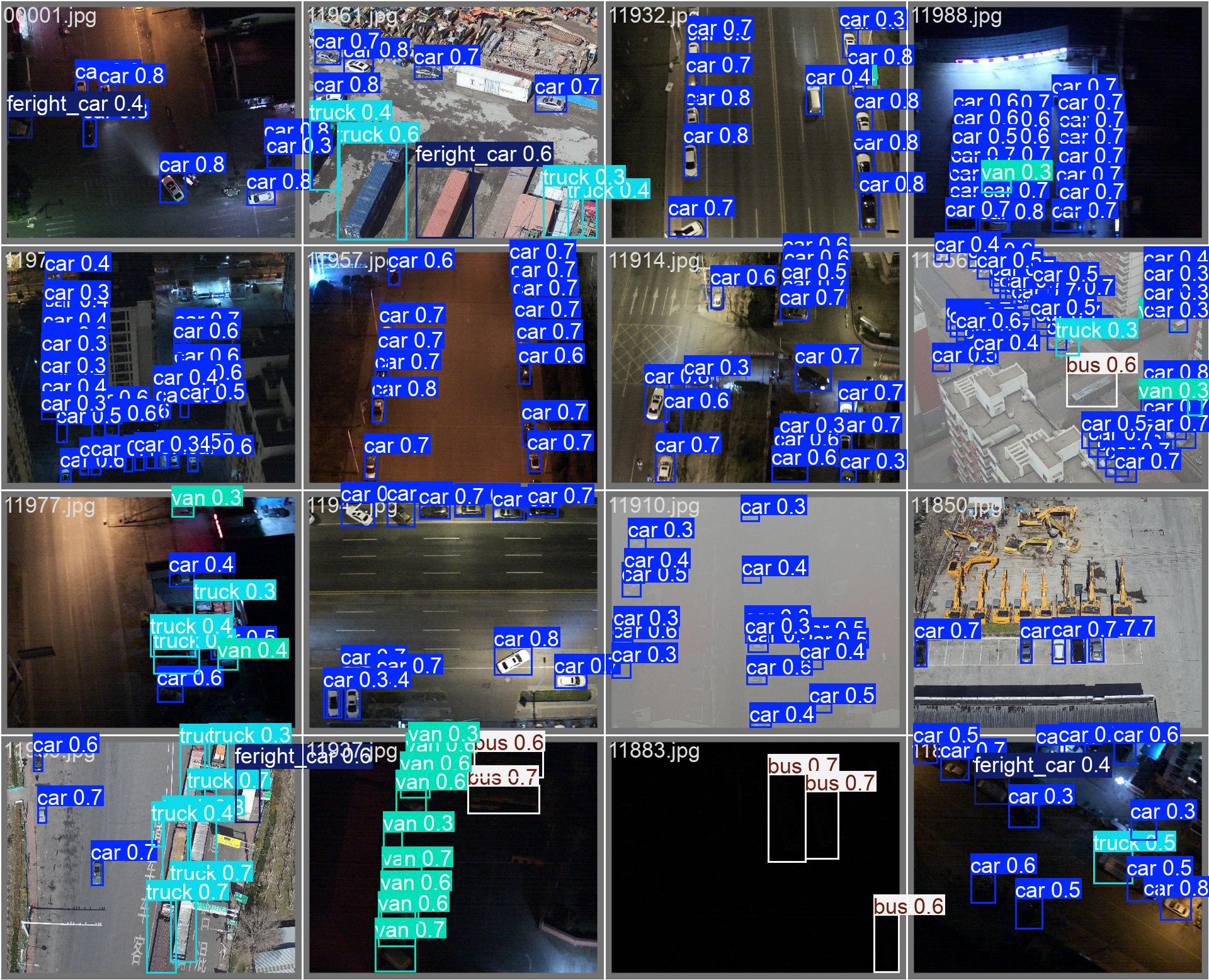

Supplement: S1 File — (ZIP) [file pone.0328248.s001.zip › S1 Model training result data/FPS/Drone Vehicle/YOLO11/val_batch0_pred.jpg]

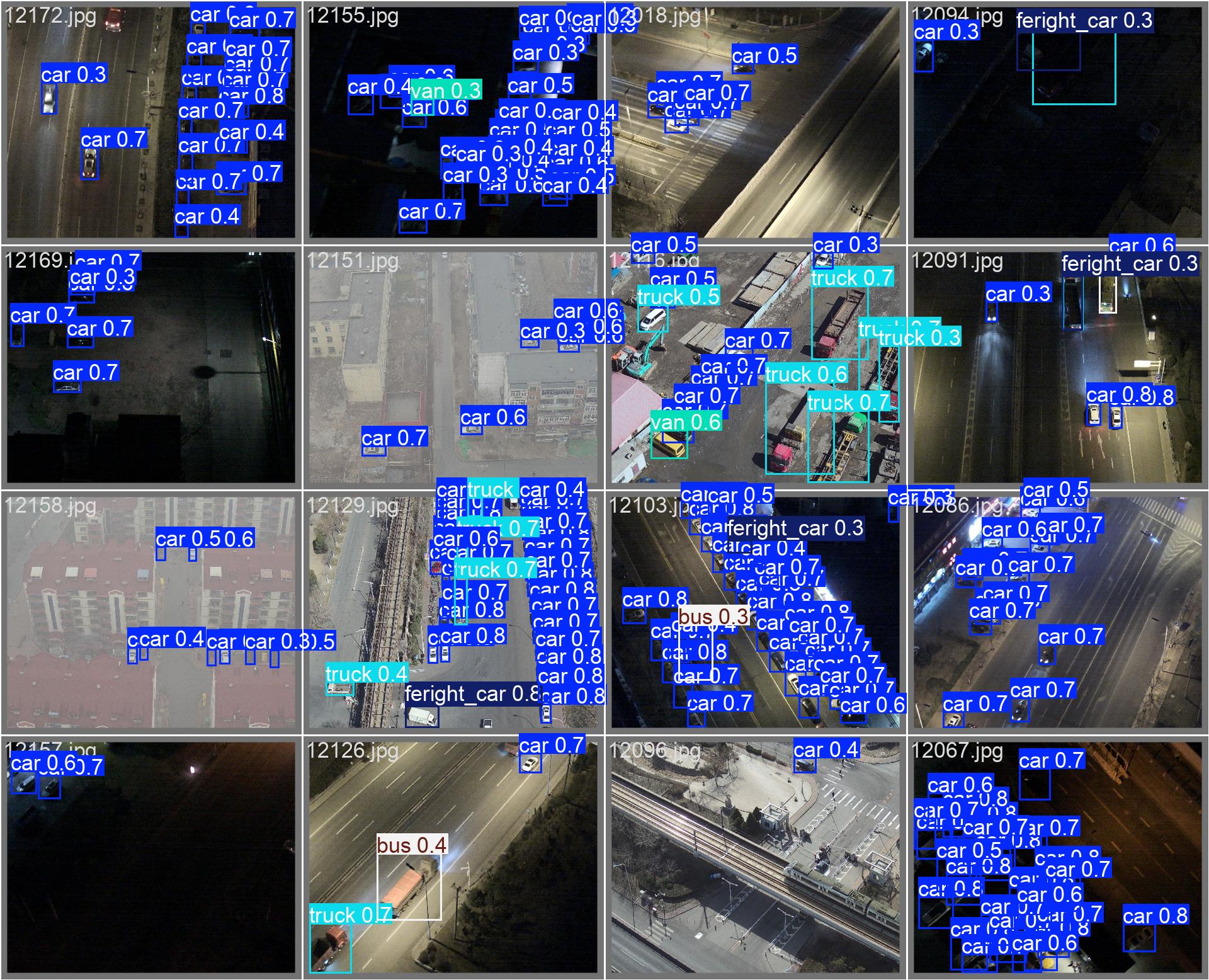

Supplement: S1 File — (ZIP) [file pone.0328248.s001.zip › S1 Model training result data/FPS/Drone Vehicle/YOLO11/val_batch1_pred.jpg]

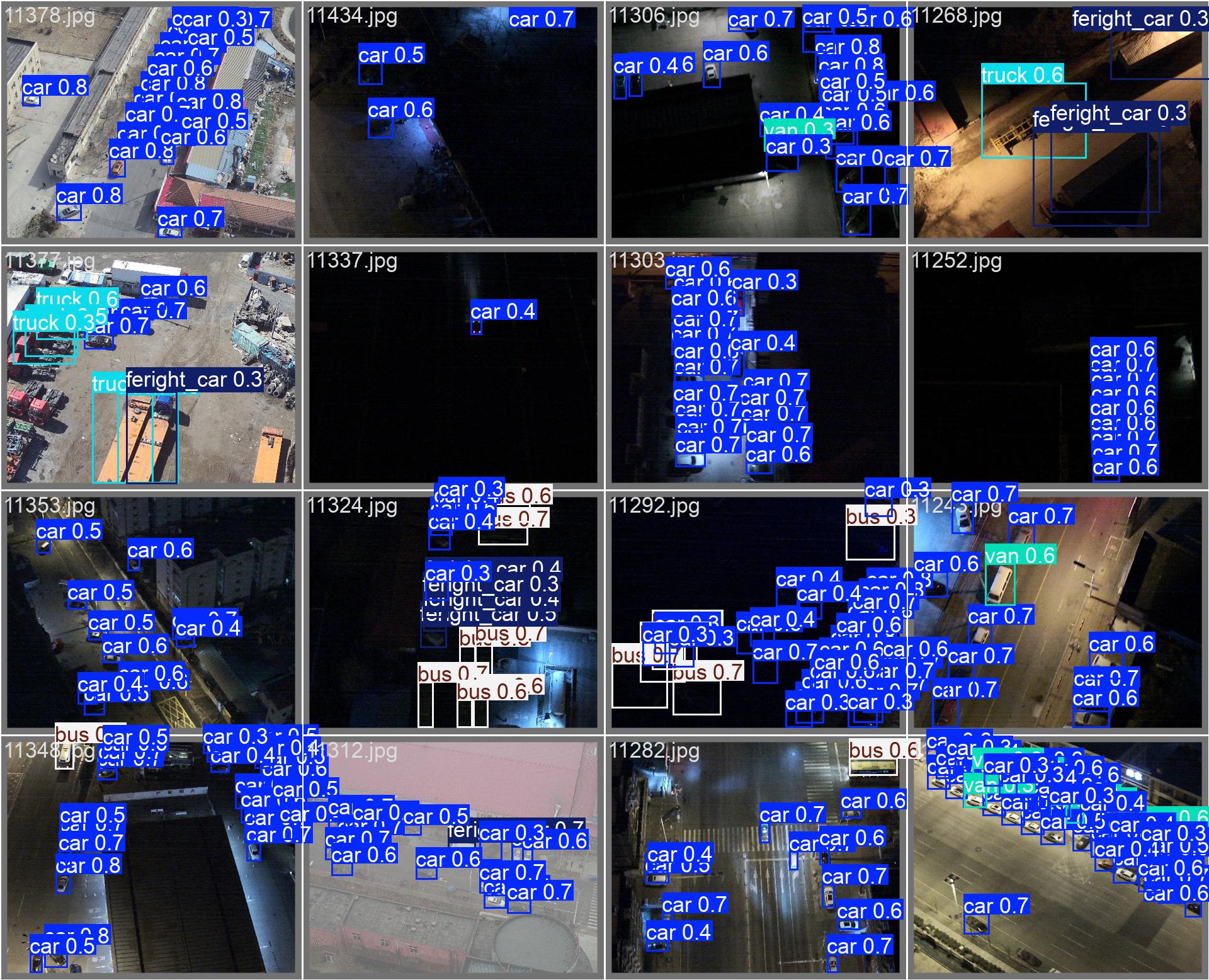

Supplement: S1 File — (ZIP) [file pone.0328248.s001.zip › S1 Model training result data/FPS/Drone Vehicle/YOLO11/val_batch2_pred.jpg]

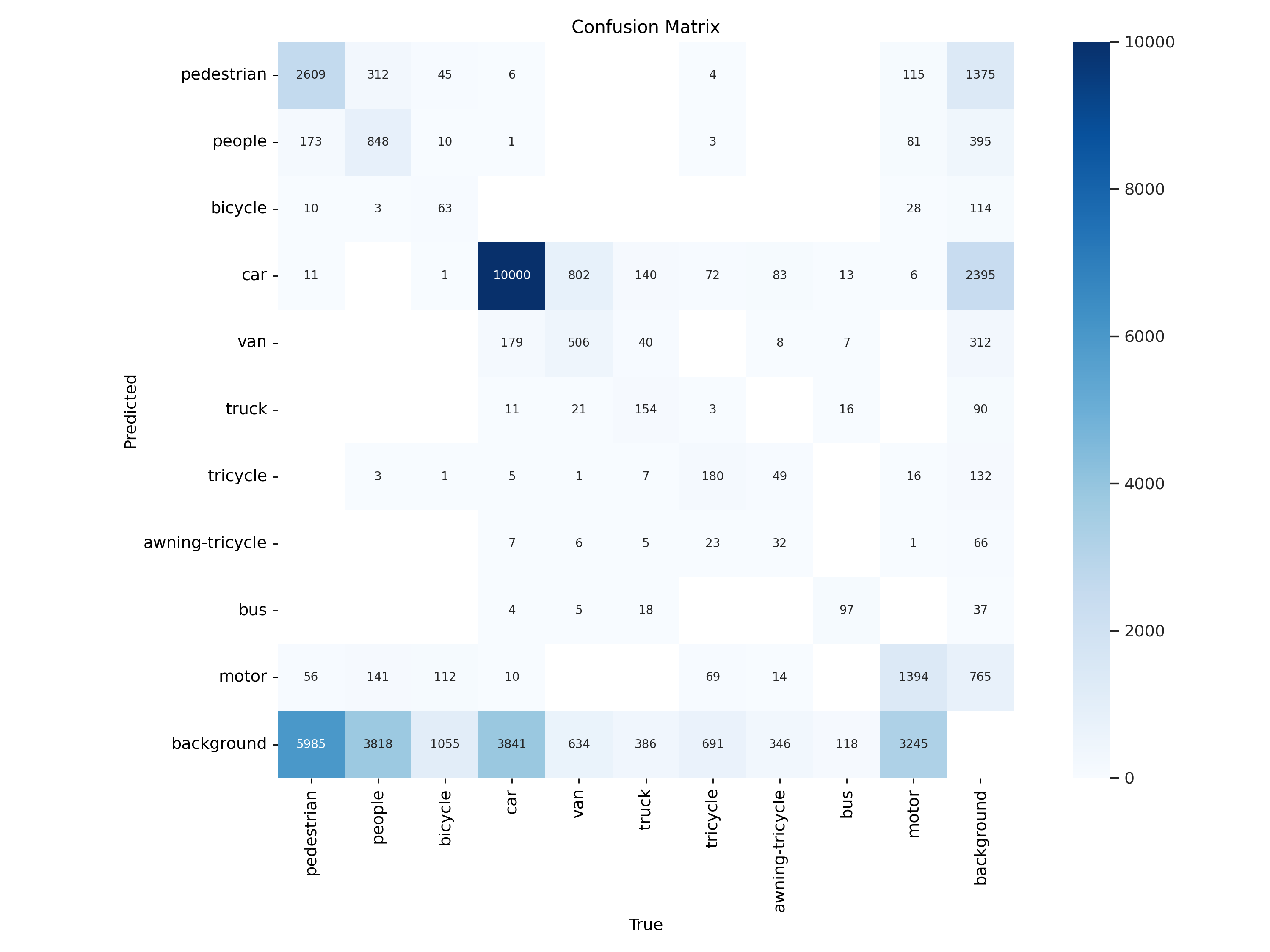

Supplement: S1 File — (ZIP) [file pone.0328248.s001.zip › S1 Model training result data/VisDrone/Train/yolo11n/confusion_matrix.png]

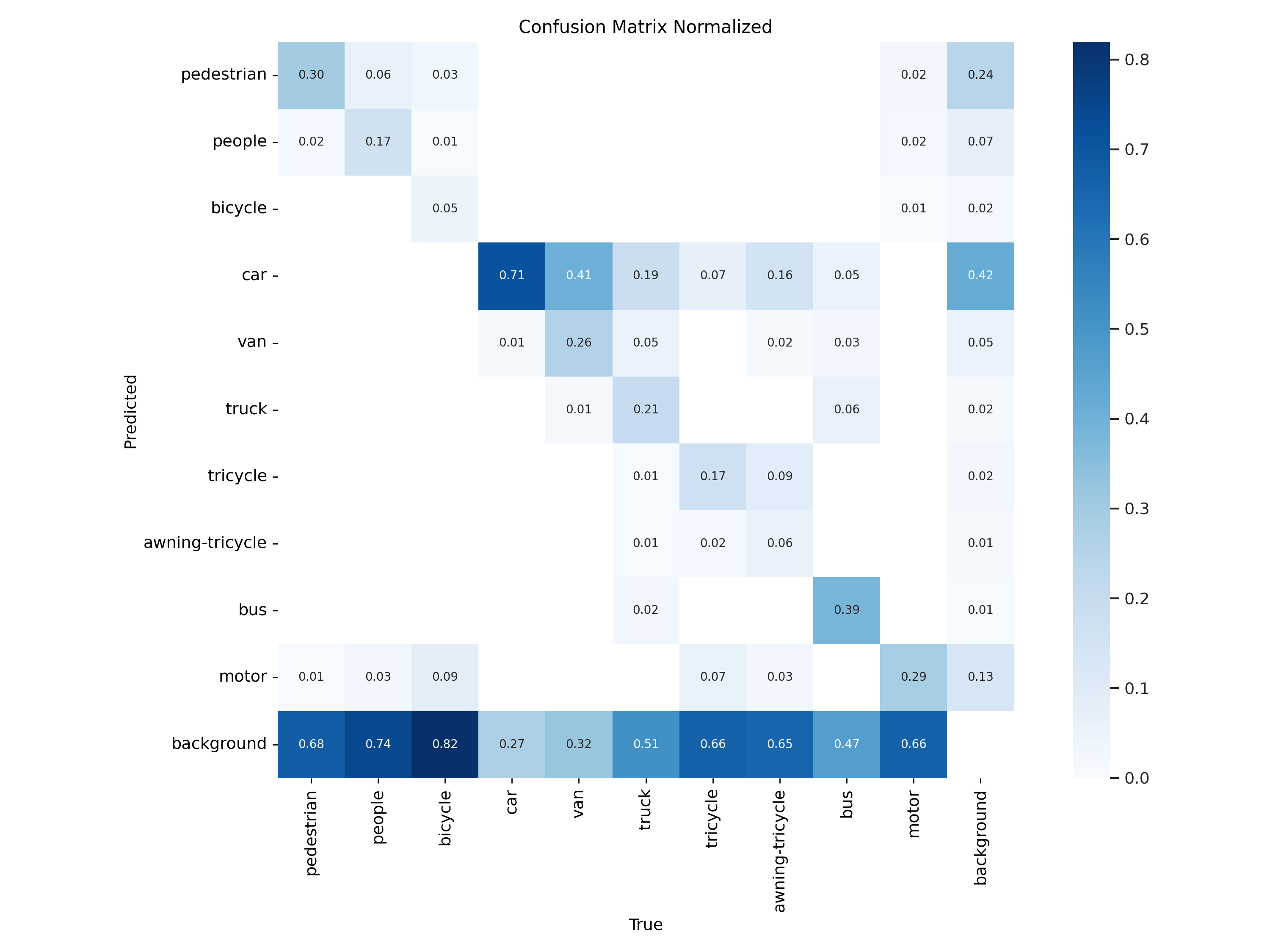

Supplement: S1 File — (ZIP) [file pone.0328248.s001.zip › S1 Model training result data/VisDrone/Train/yolo11n/confusion_matrix_normalized.png]

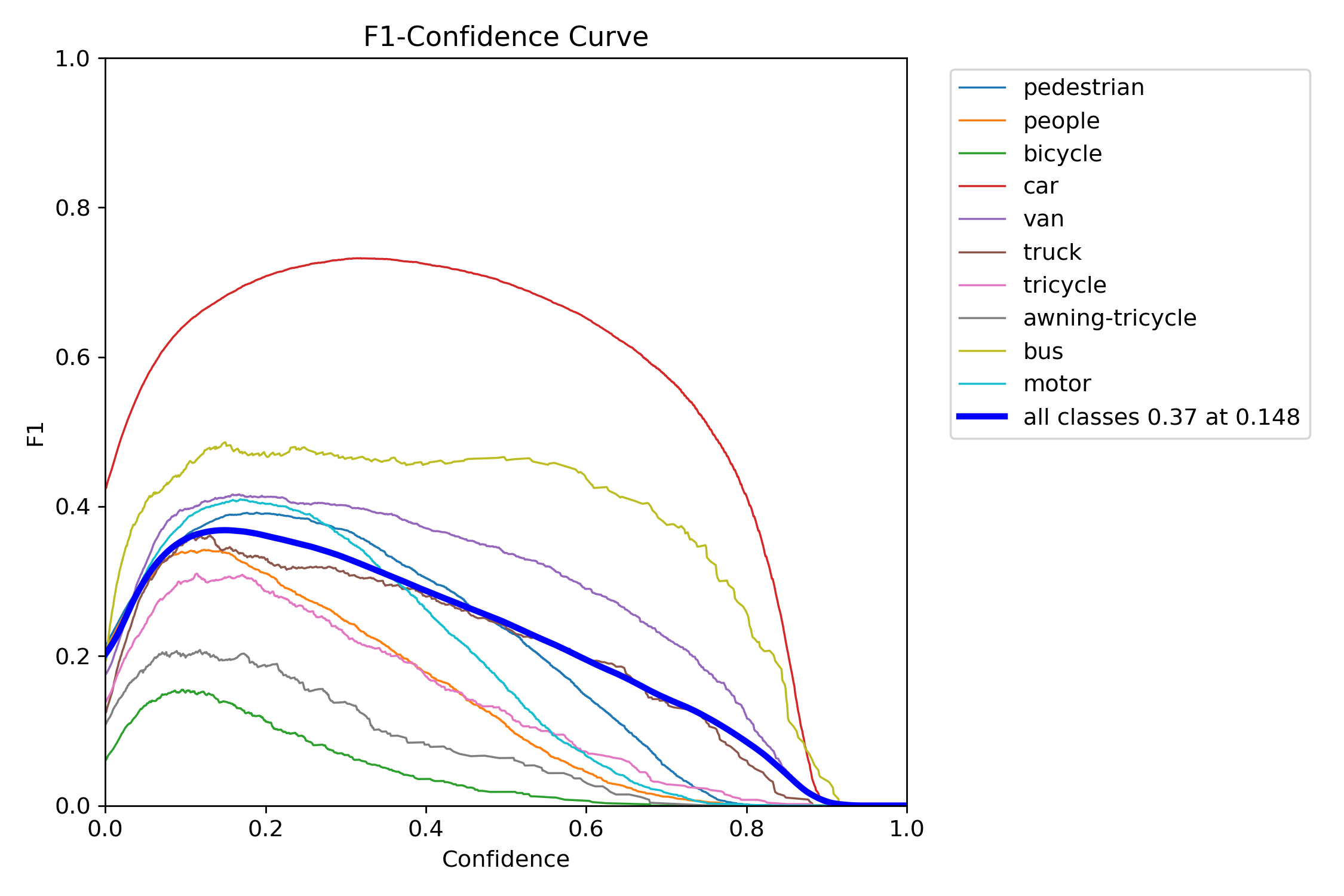

Supplement: S1 File — (ZIP) [file pone.0328248.s001.zip › S1 Model training result data/VisDrone/Train/yolo11n/F1_curve.png]

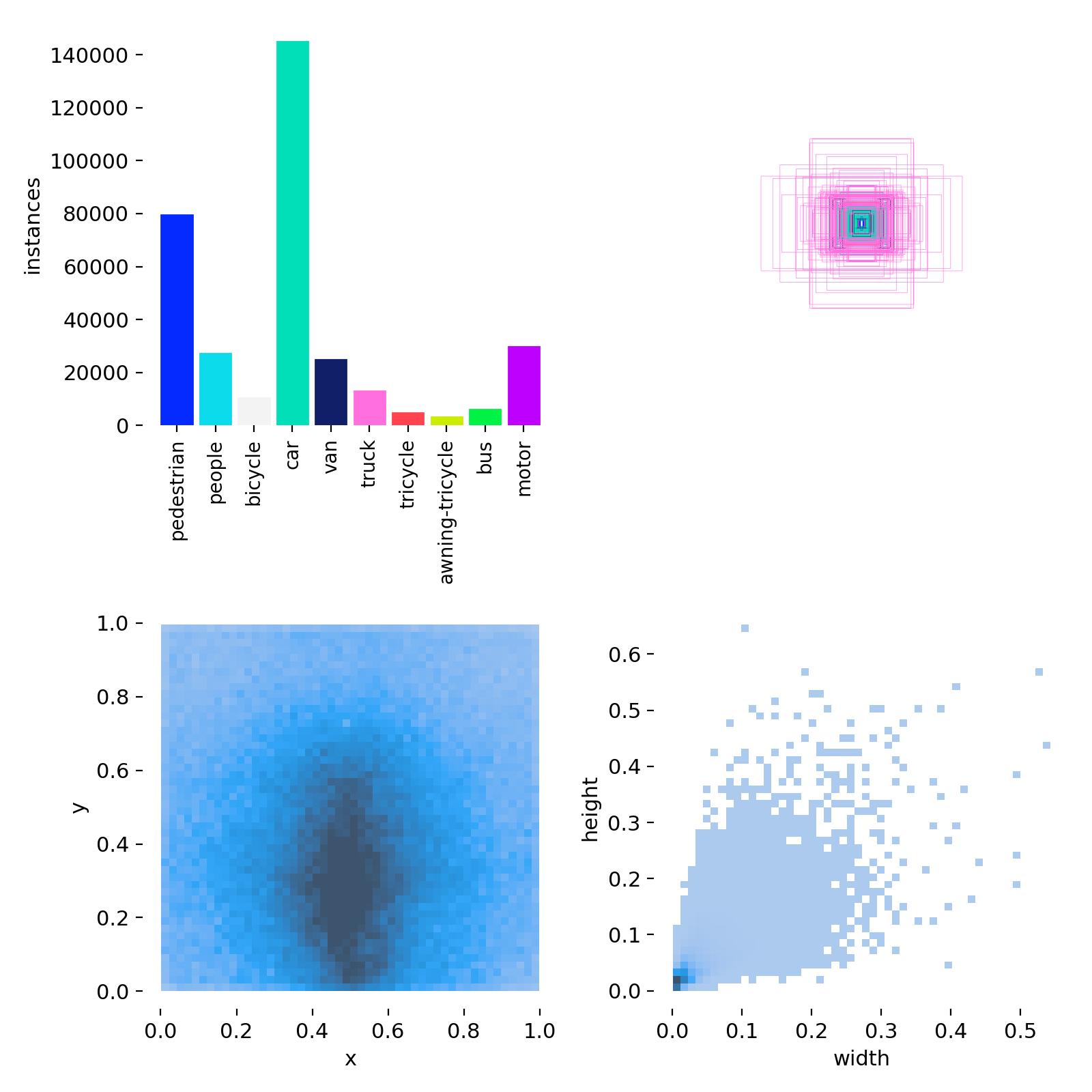

Supplement: S1 File — (ZIP) [file pone.0328248.s001.zip › S1 Model training result data/VisDrone/Train/yolo11n/labels.jpg]

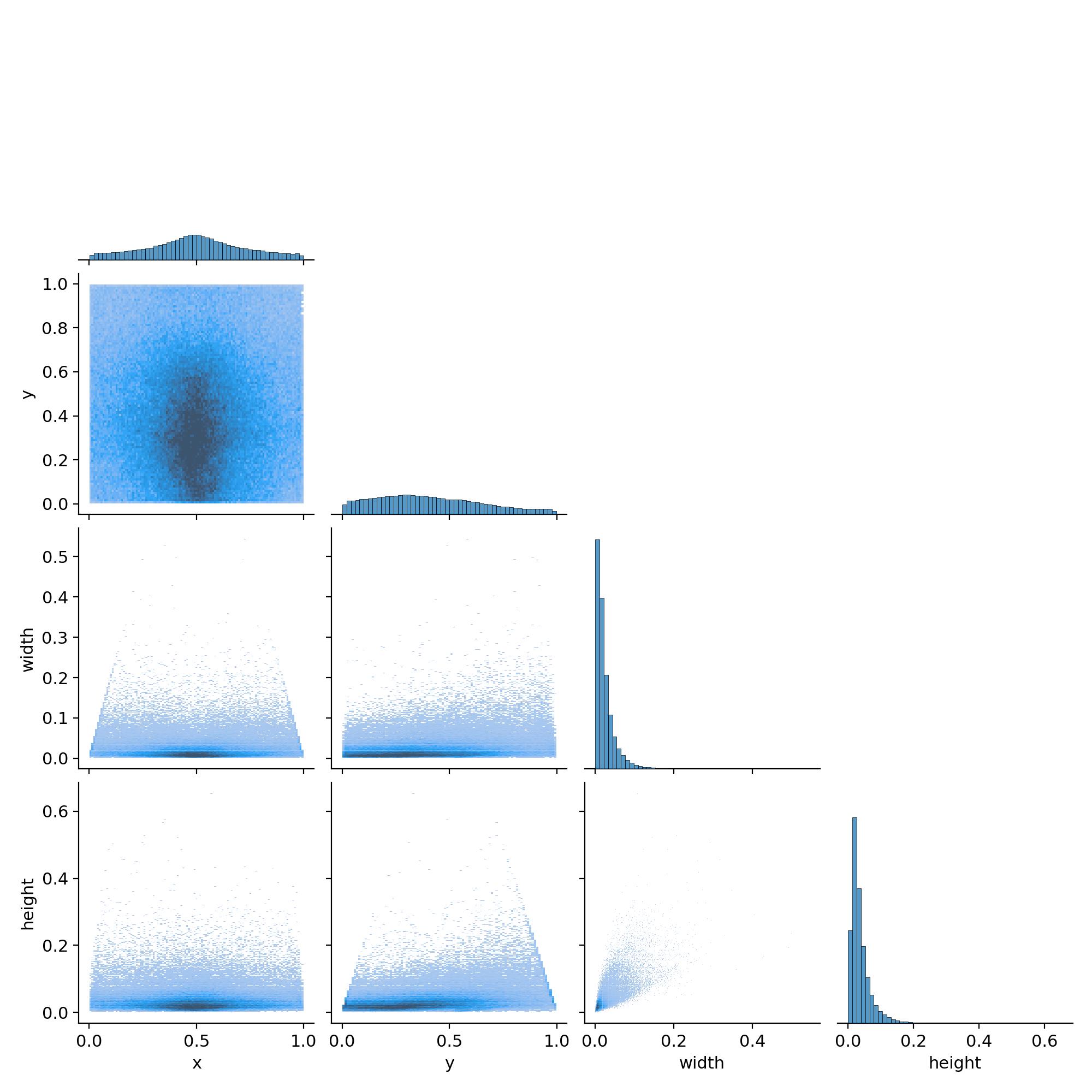

Supplement: S1 File — (ZIP) [file pone.0328248.s001.zip › S1 Model training result data/VisDrone/Train/yolo11n/labels_correlogram.jpg]

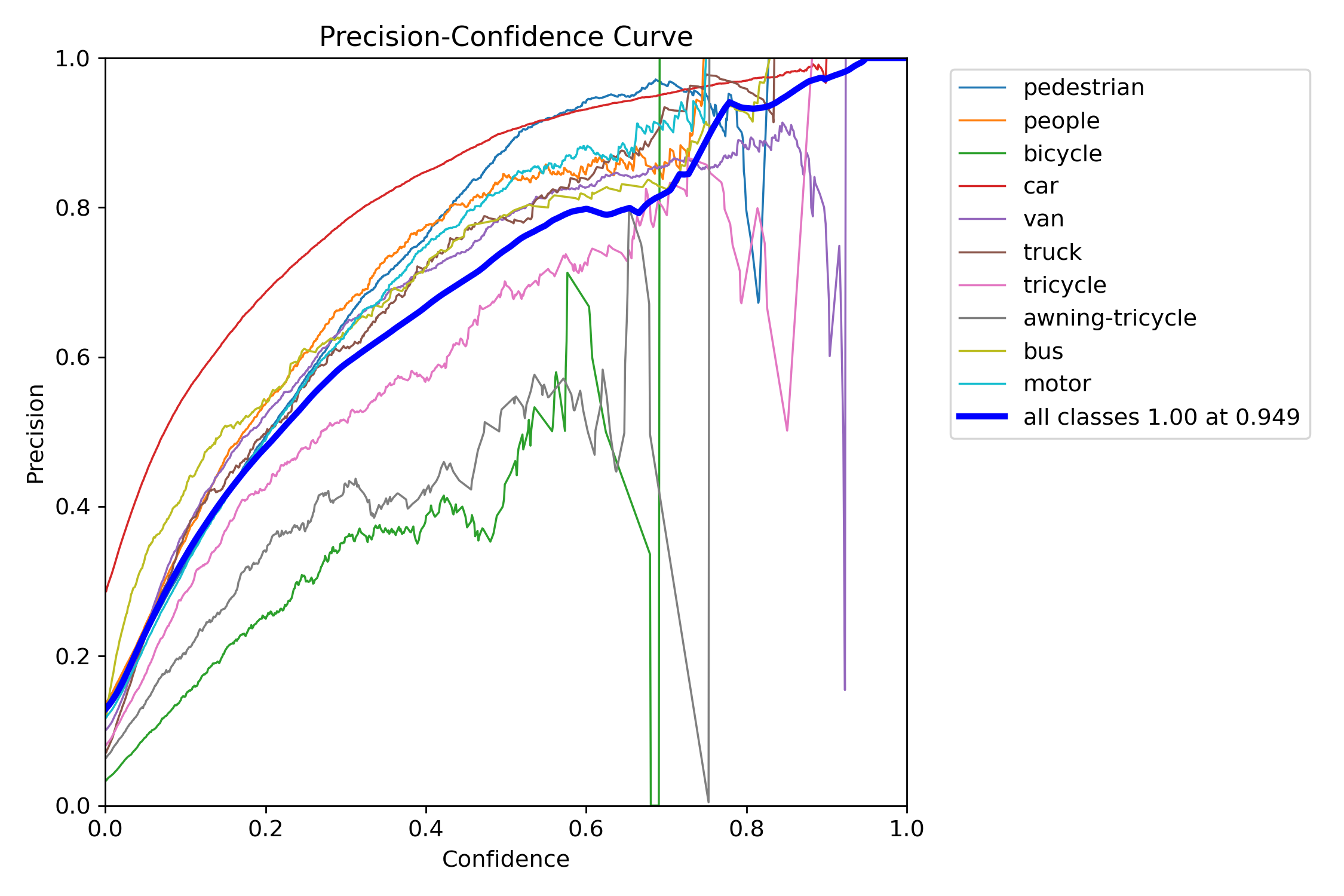

Supplement: S1 File — (ZIP) [file pone.0328248.s001.zip › S1 Model training result data/VisDrone/Train/yolo11n/P_curve.png]

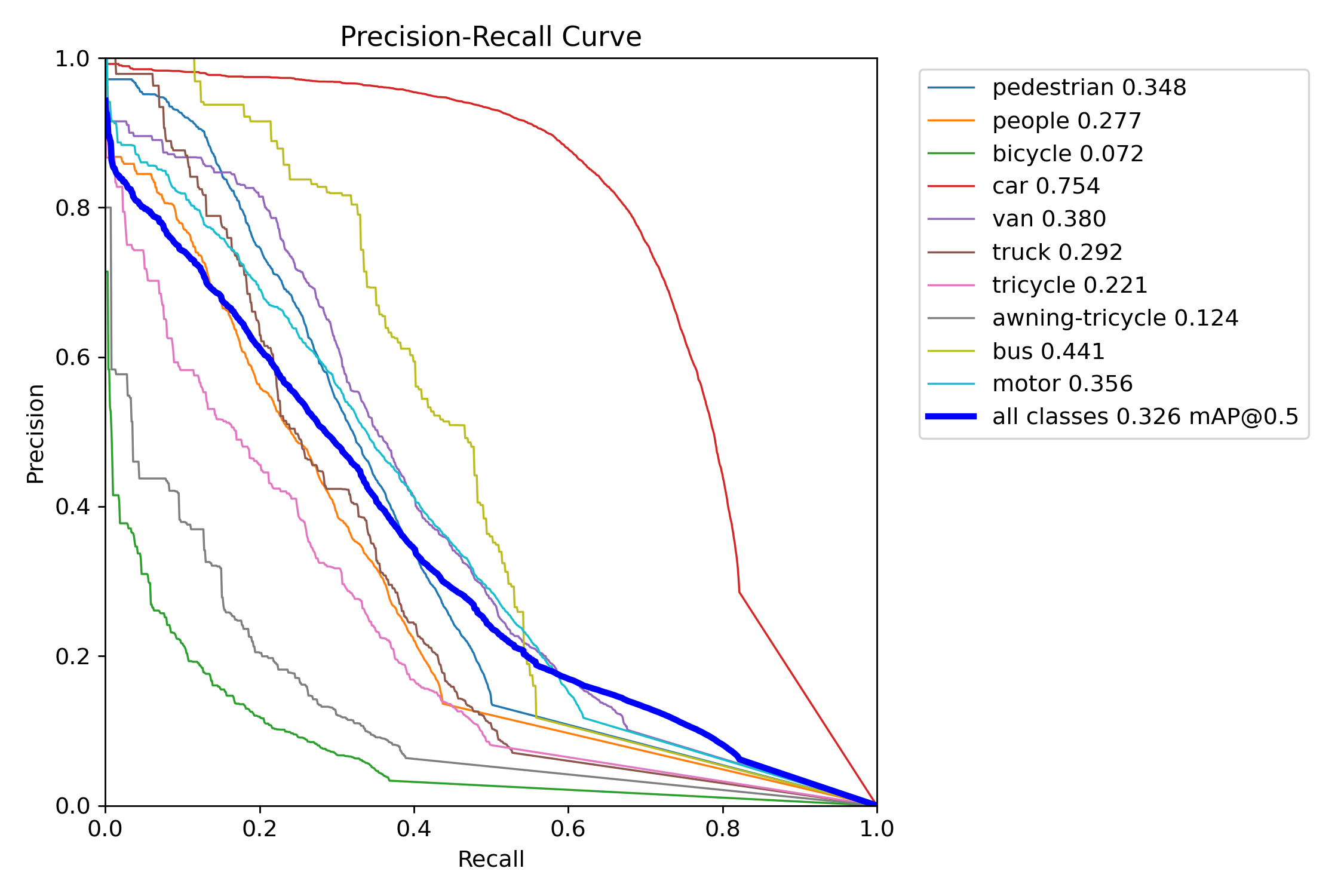

Supplement: S1 File — (ZIP) [file pone.0328248.s001.zip › S1 Model training result data/VisDrone/Train/yolo11n/PR_curve.png]

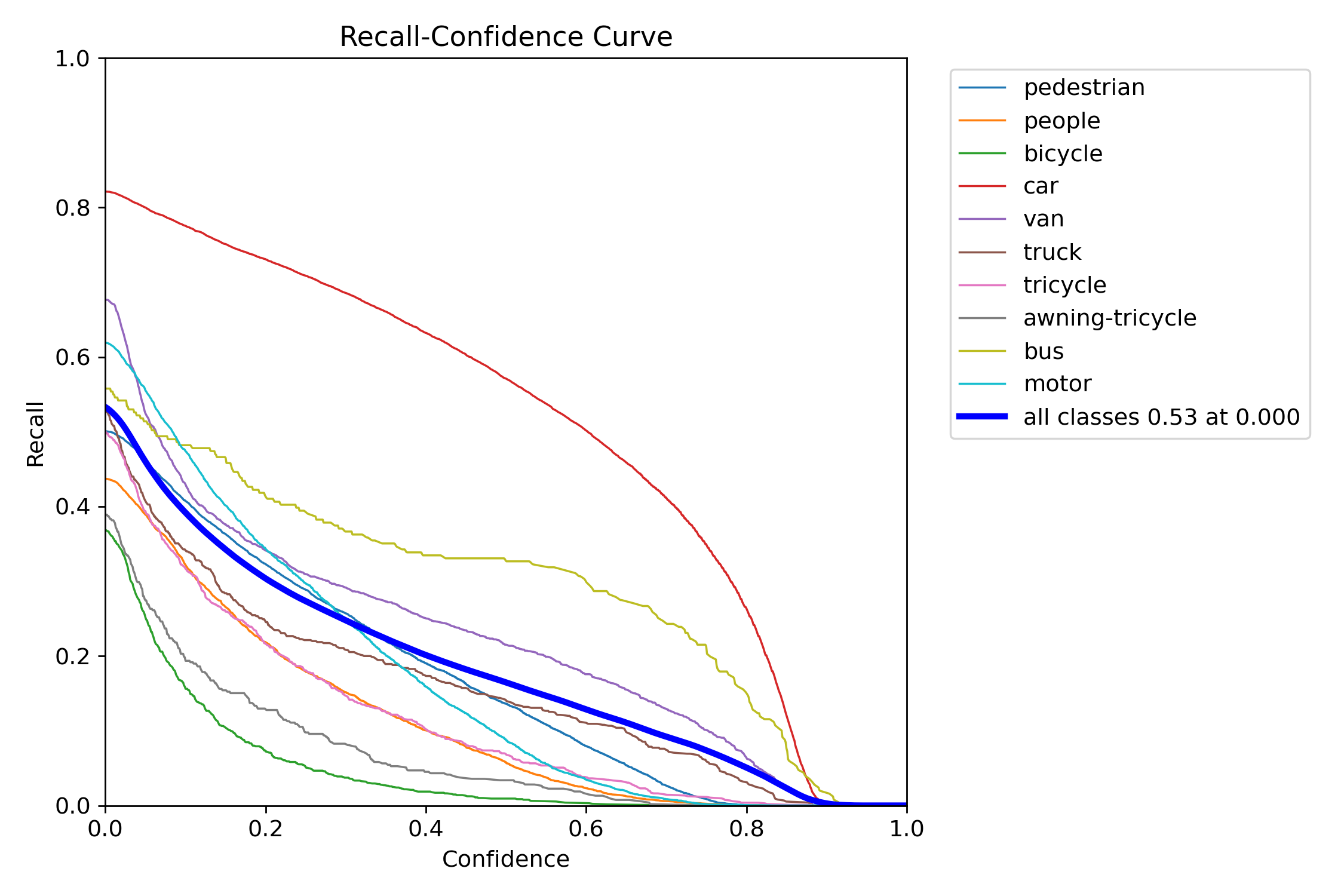

Supplement: S1 File — (ZIP) [file pone.0328248.s001.zip › S1 Model training result data/VisDrone/Train/yolo11n/R_curve.png]

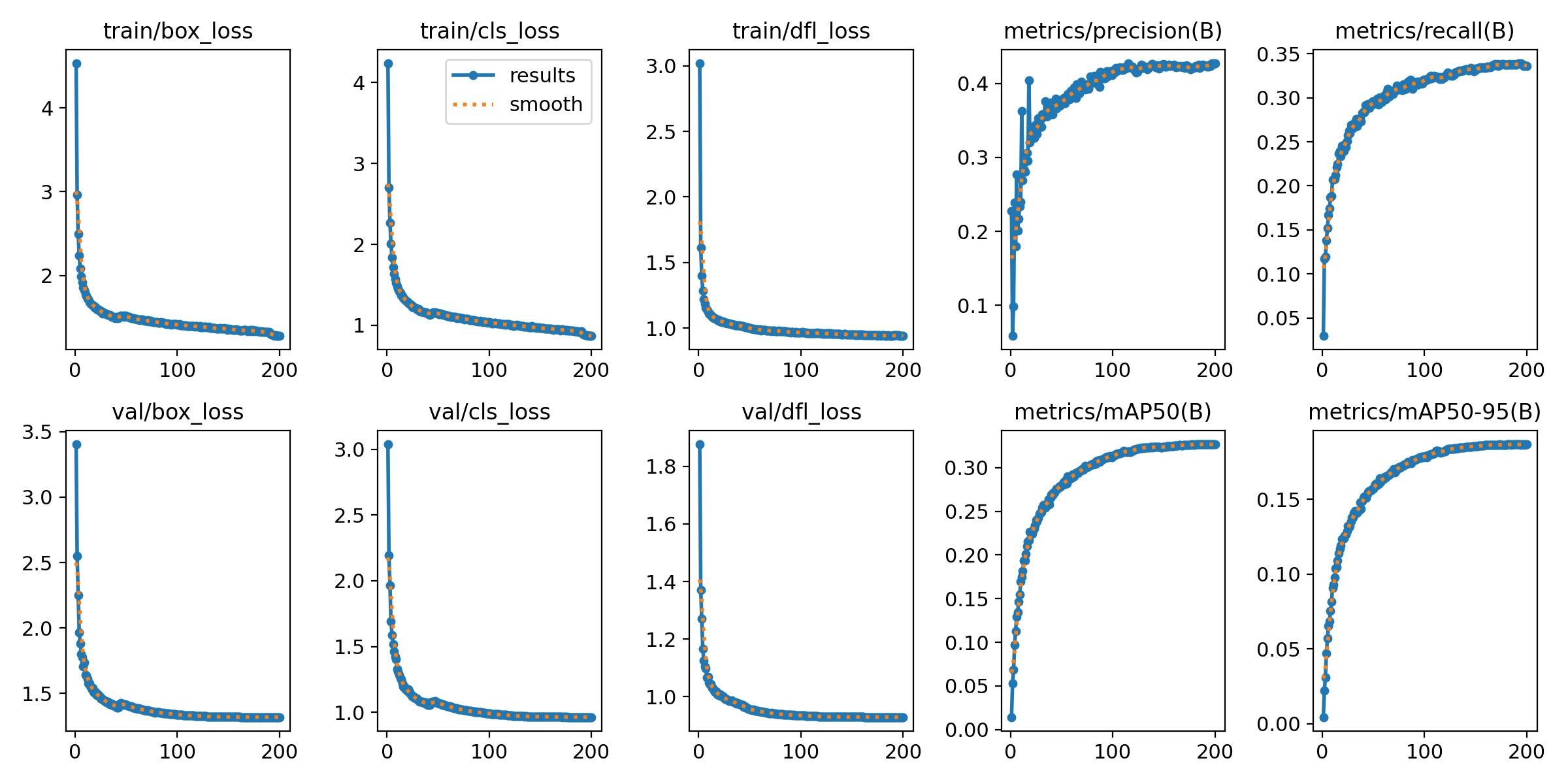

Supplement: S1 File — (ZIP) [file pone.0328248.s001.zip › S1 Model training result data/VisDrone/Train/yolo11n/results.png]

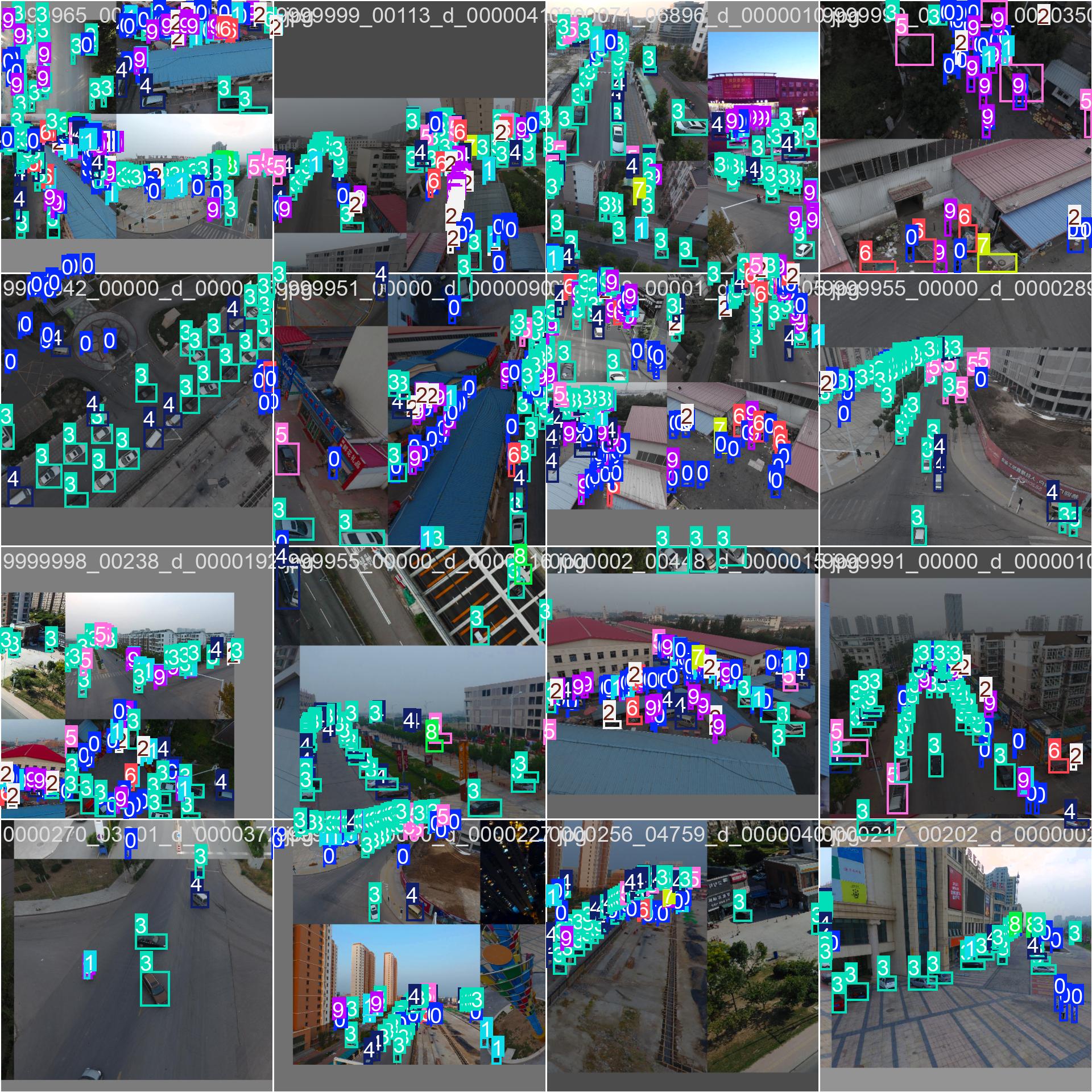

Supplement: S1 File — (ZIP) [file pone.0328248.s001.zip › S1 Model training result data/VisDrone/Train/yolo11n/train_batch0.jpg]

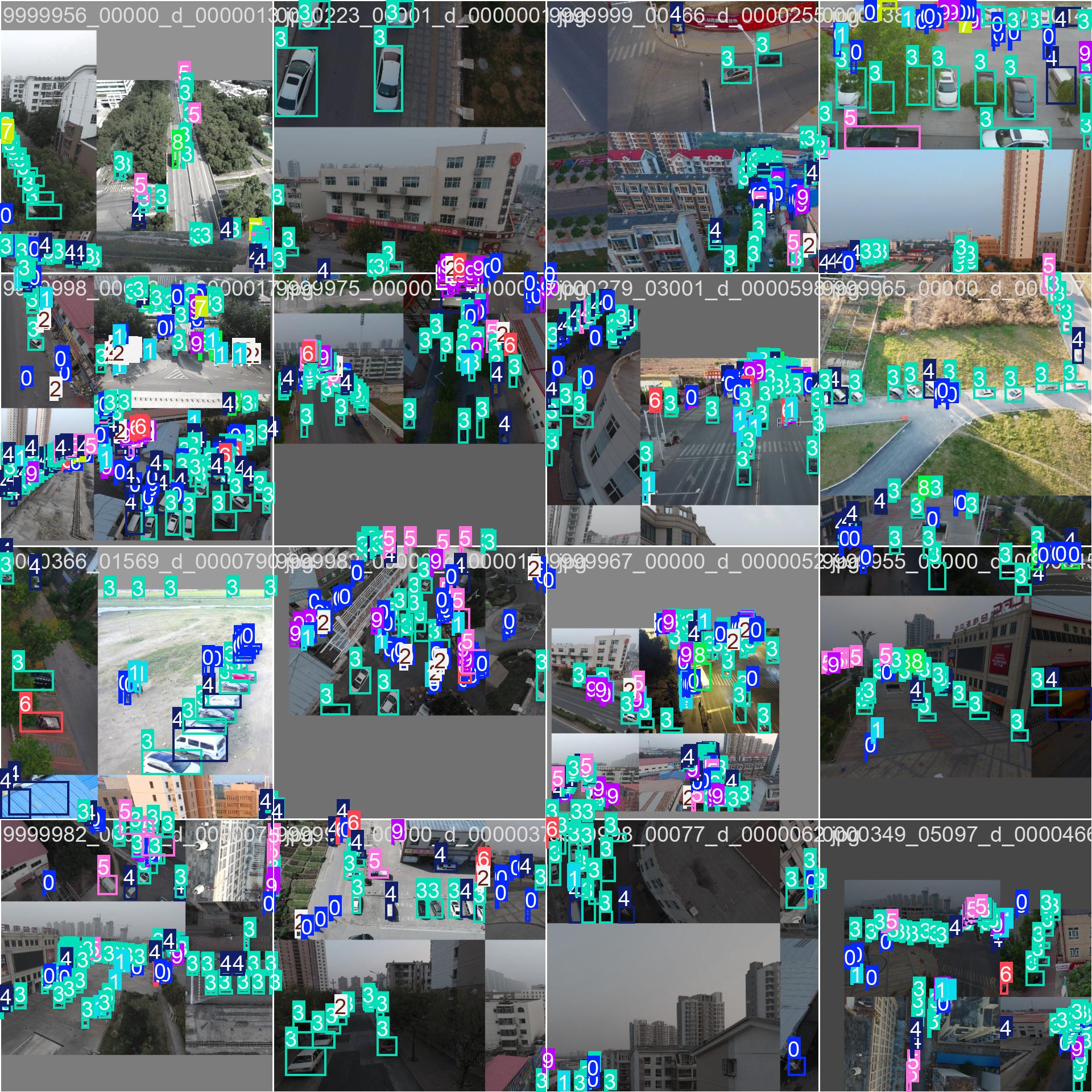

Supplement: S1 File — (ZIP) [file pone.0328248.s001.zip › S1 Model training result data/VisDrone/Train/yolo11n/train_batch1.jpg]

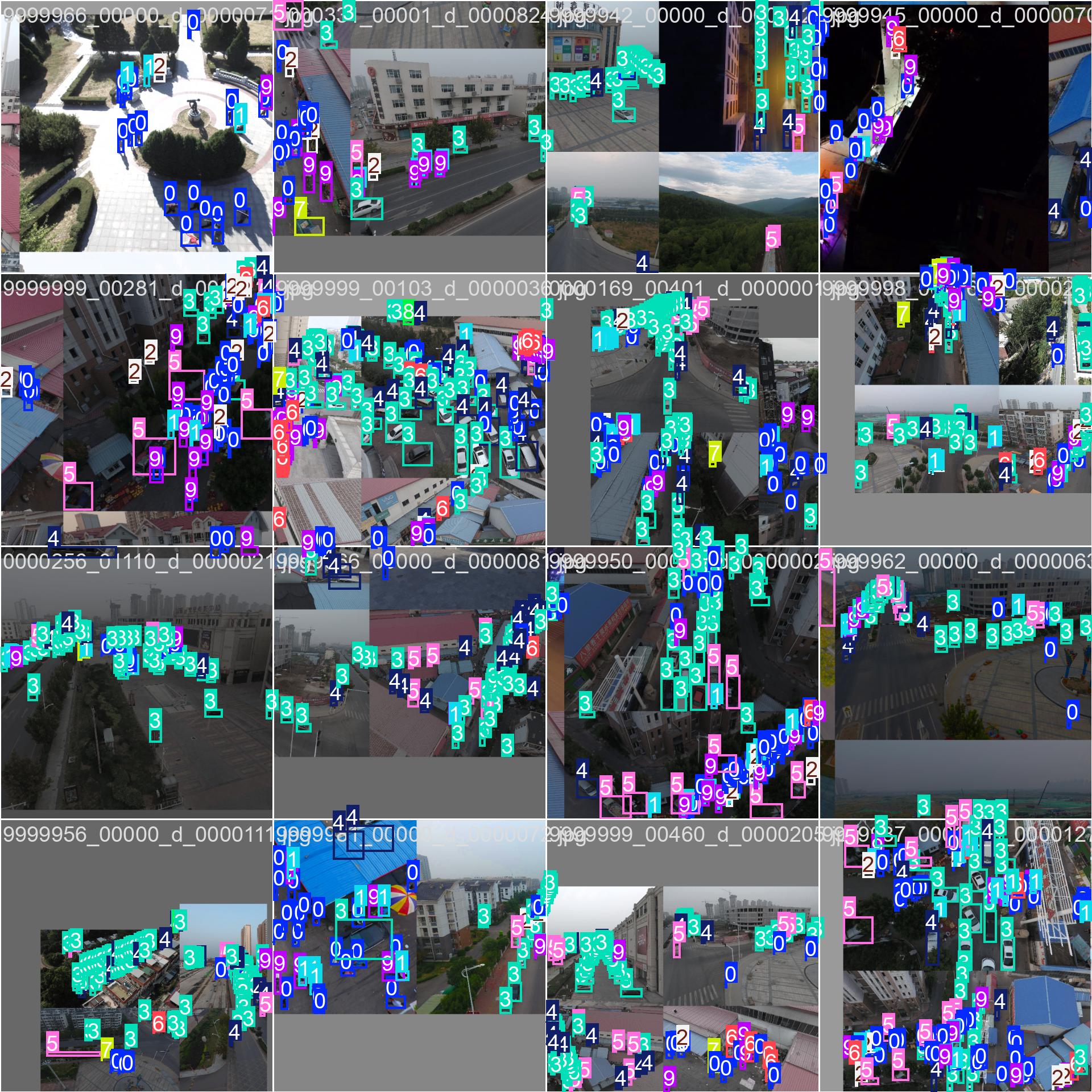

Supplement: S1 File — (ZIP) [file pone.0328248.s001.zip › S1 Model training result data/VisDrone/Train/yolo11n/train_batch2.jpg]

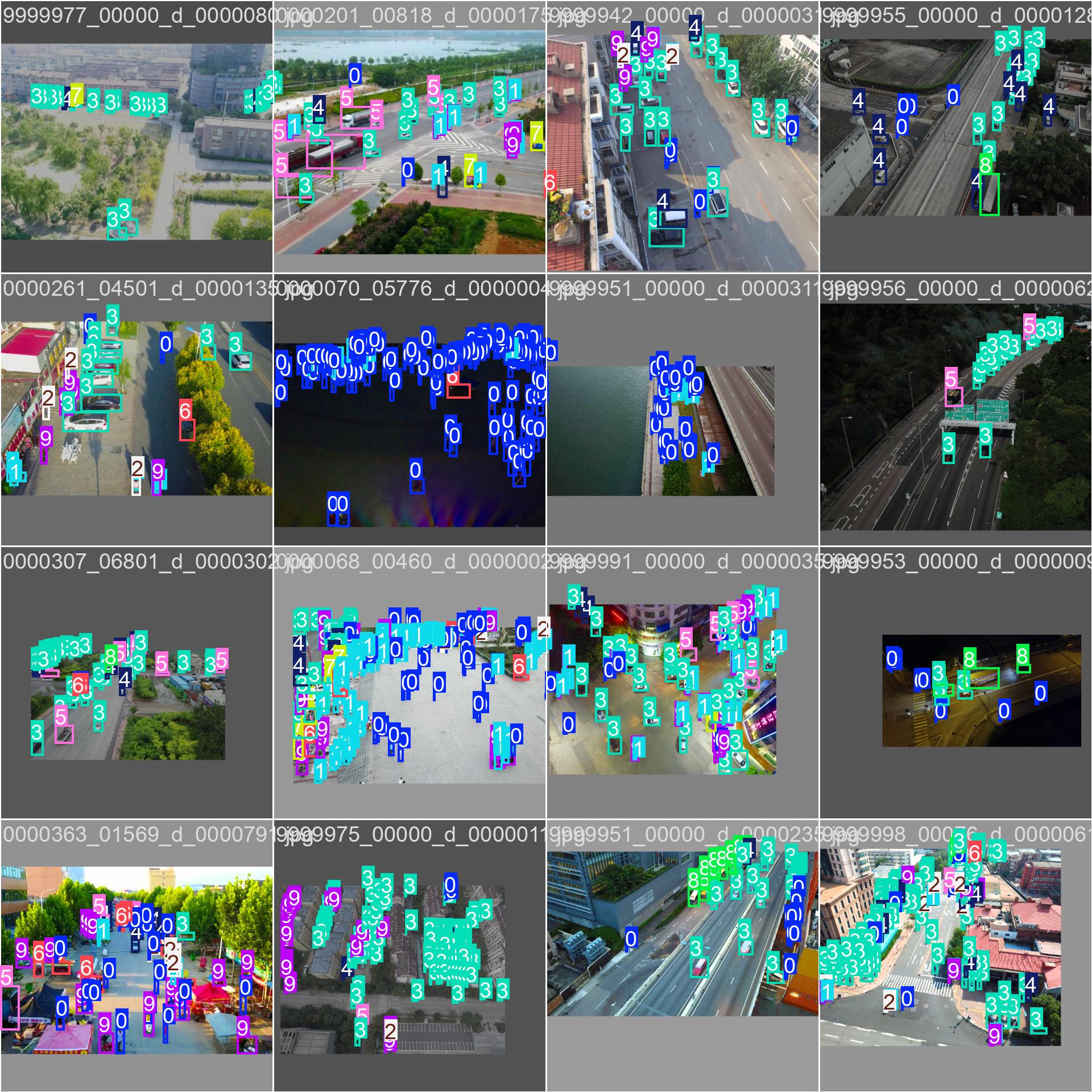

Supplement: S1 File — (ZIP) [file pone.0328248.s001.zip › S1 Model training result data/VisDrone/Train/yolo11n/train_batch38570.jpg]

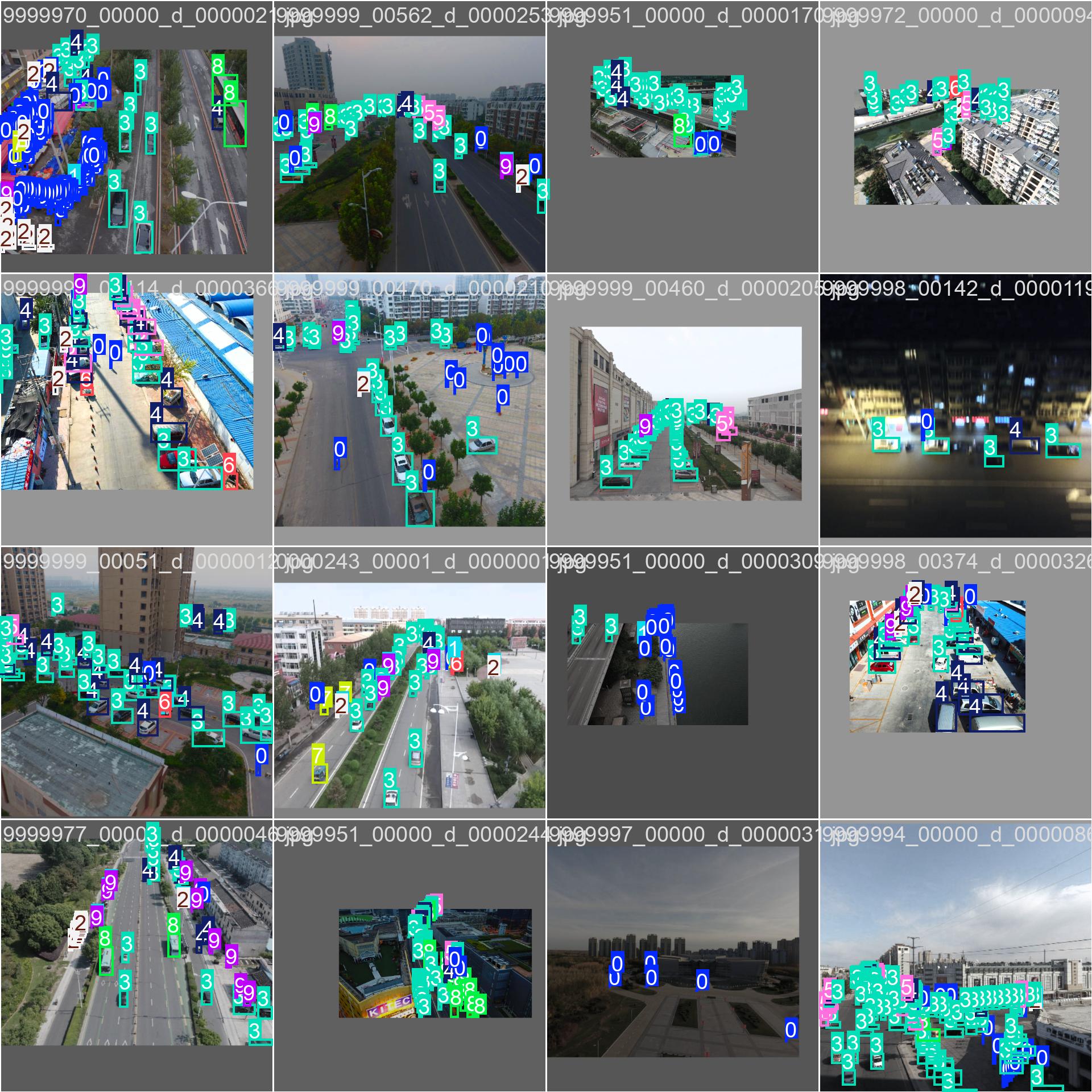

Supplement: S1 File — (ZIP) [file pone.0328248.s001.zip › S1 Model training result data/VisDrone/Train/yolo11n/train_batch38571.jpg]

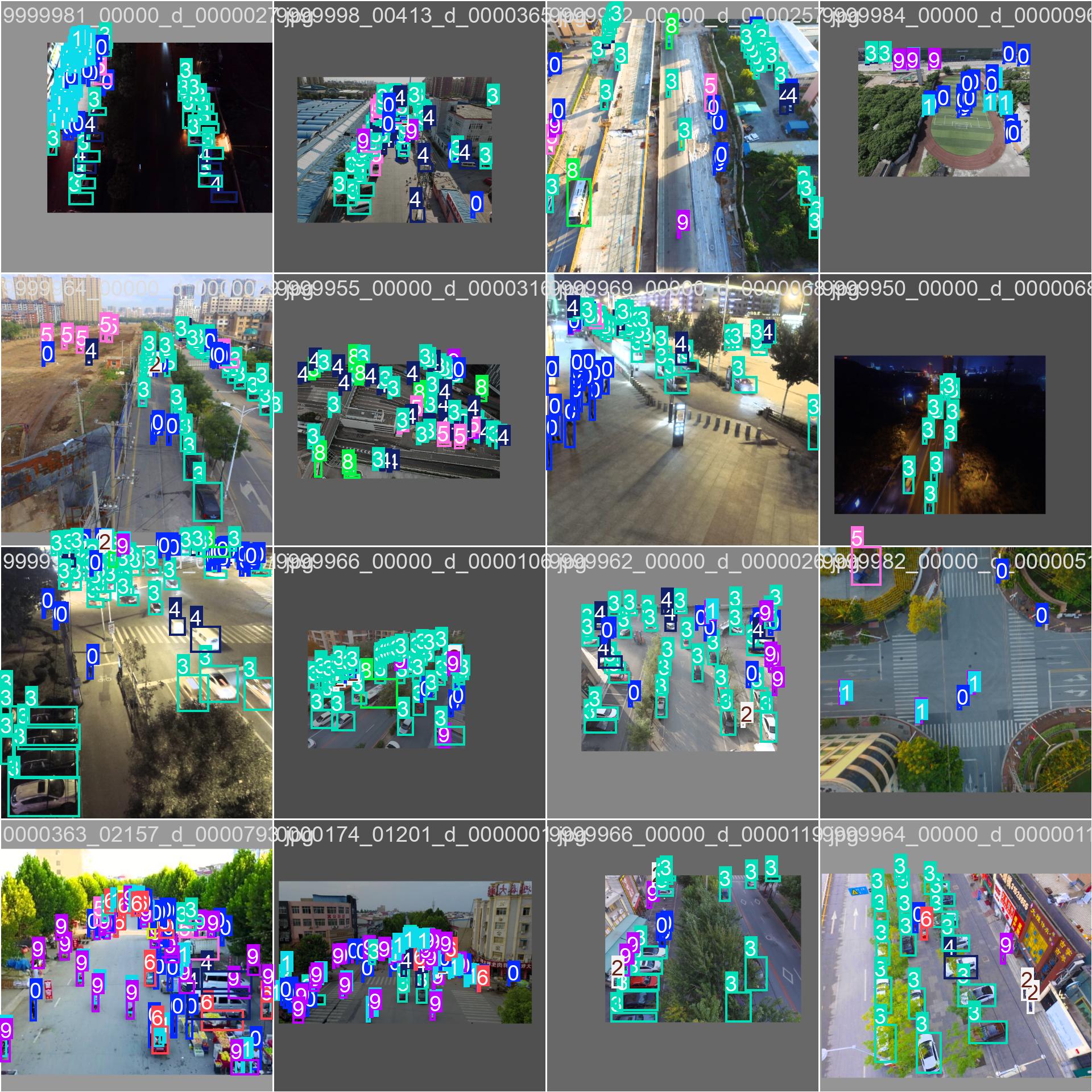

Supplement: S1 File — (ZIP) [file pone.0328248.s001.zip › S1 Model training result data/VisDrone/Train/yolo11n/train_batch38572.jpg]

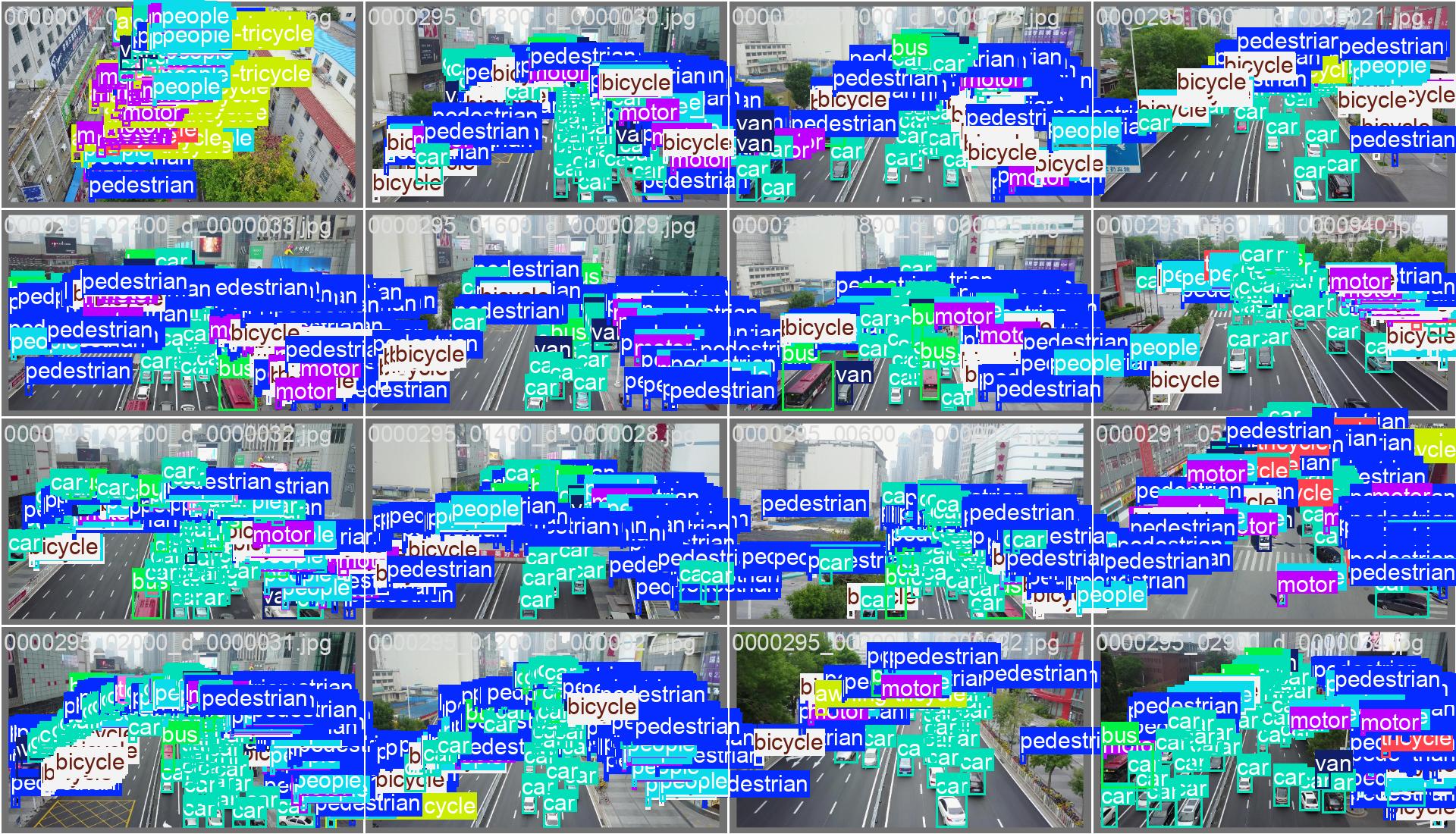

Supplement: S1 File — (ZIP) [file pone.0328248.s001.zip › S1 Model training result data/VisDrone/Train/yolo11n/val_batch0_labels.jpg]

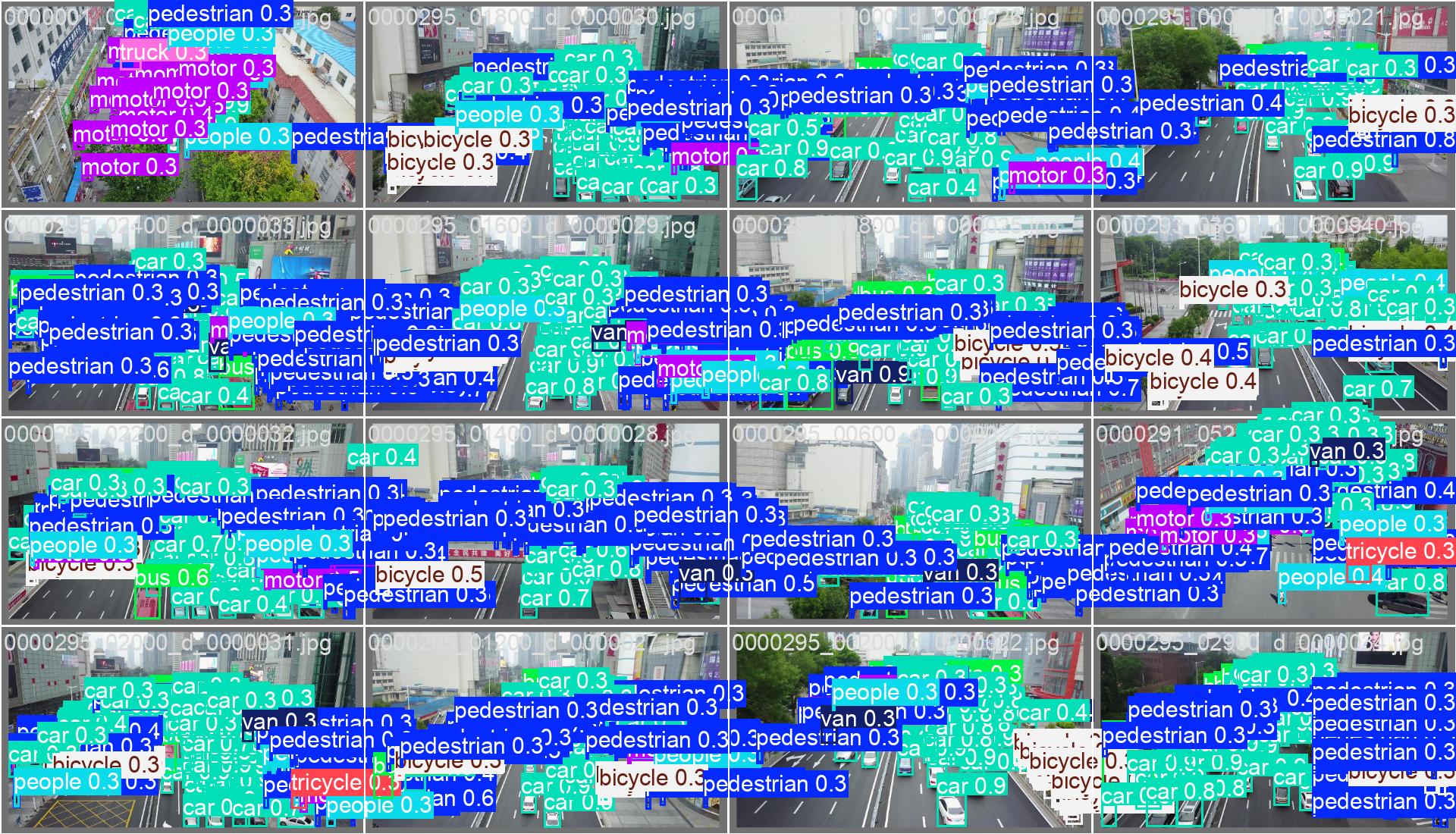

Supplement: S1 File — (ZIP) [file pone.0328248.s001.zip › S1 Model training result data/VisDrone/Train/yolo11n/val_batch0_pred.jpg]

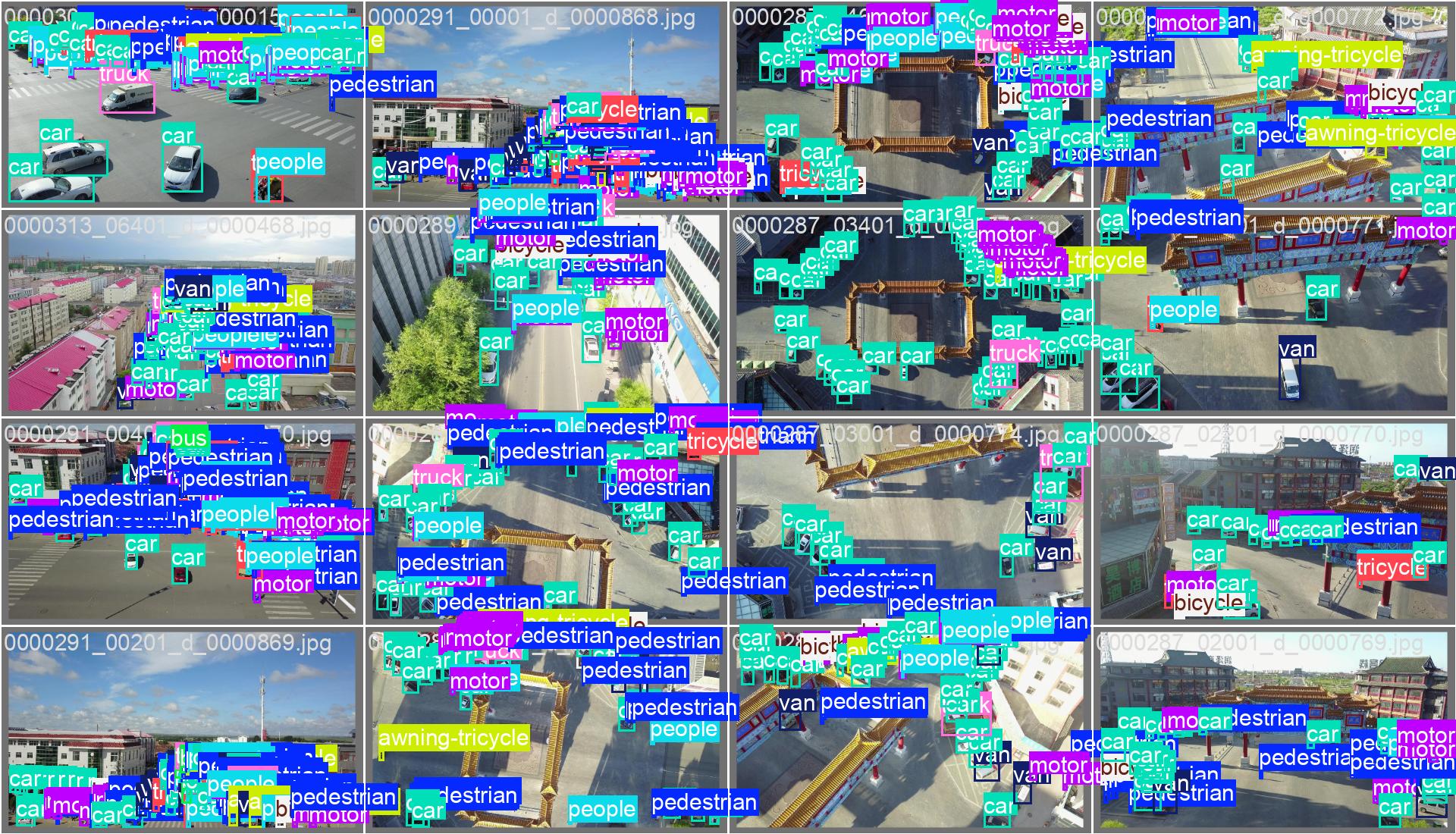

Supplement: S1 File — (ZIP) [file pone.0328248.s001.zip › S1 Model training result data/VisDrone/Train/yolo11n/val_batch1_labels.jpg]

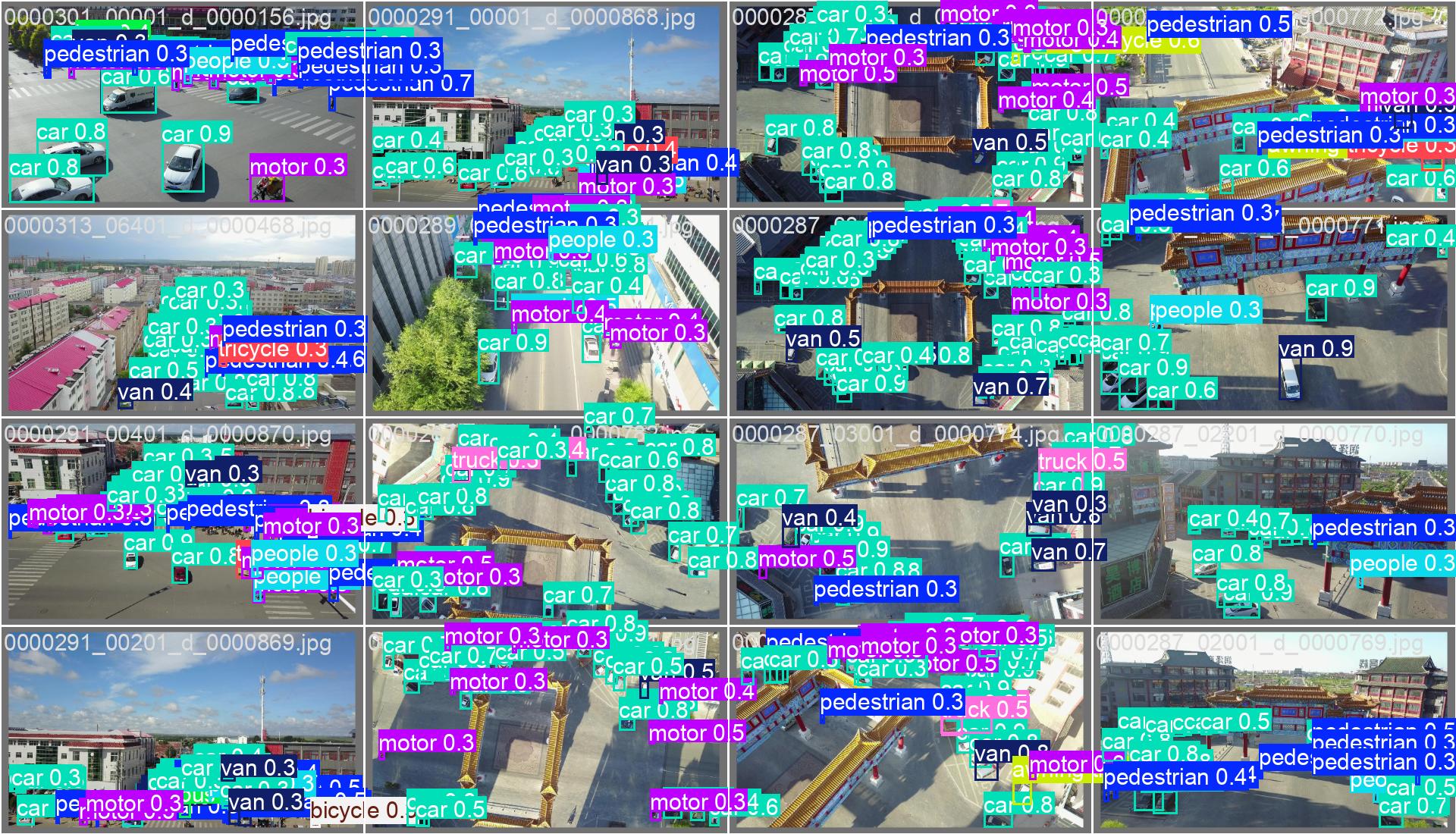

Supplement: S1 File — (ZIP) [file pone.0328248.s001.zip › S1 Model training result data/VisDrone/Train/yolo11n/val_batch1_pred.jpg]

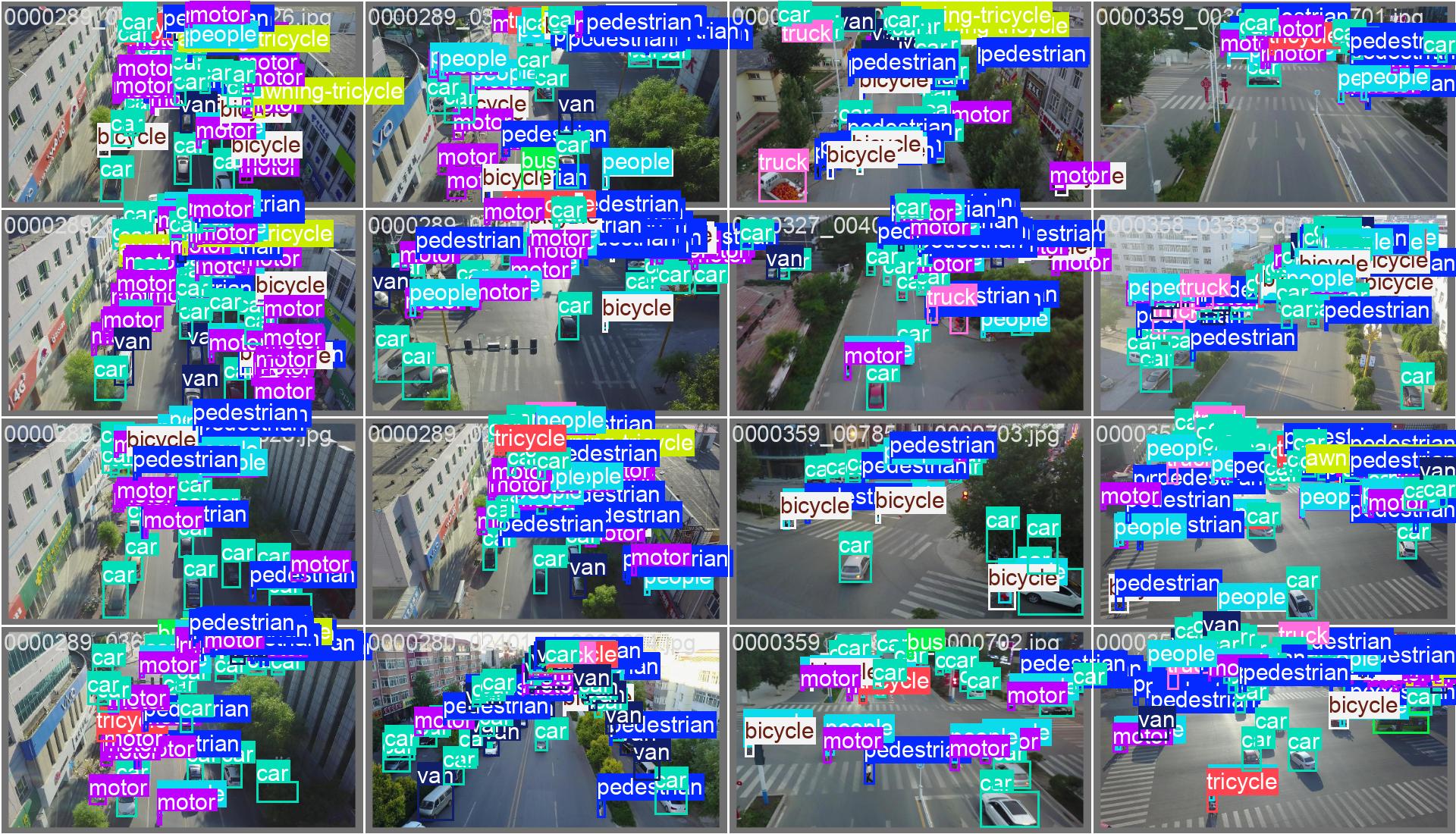

Supplement: S1 File — (ZIP) [file pone.0328248.s001.zip › S1 Model training result data/VisDrone/Train/yolo11n/val_batch2_labels.jpg]

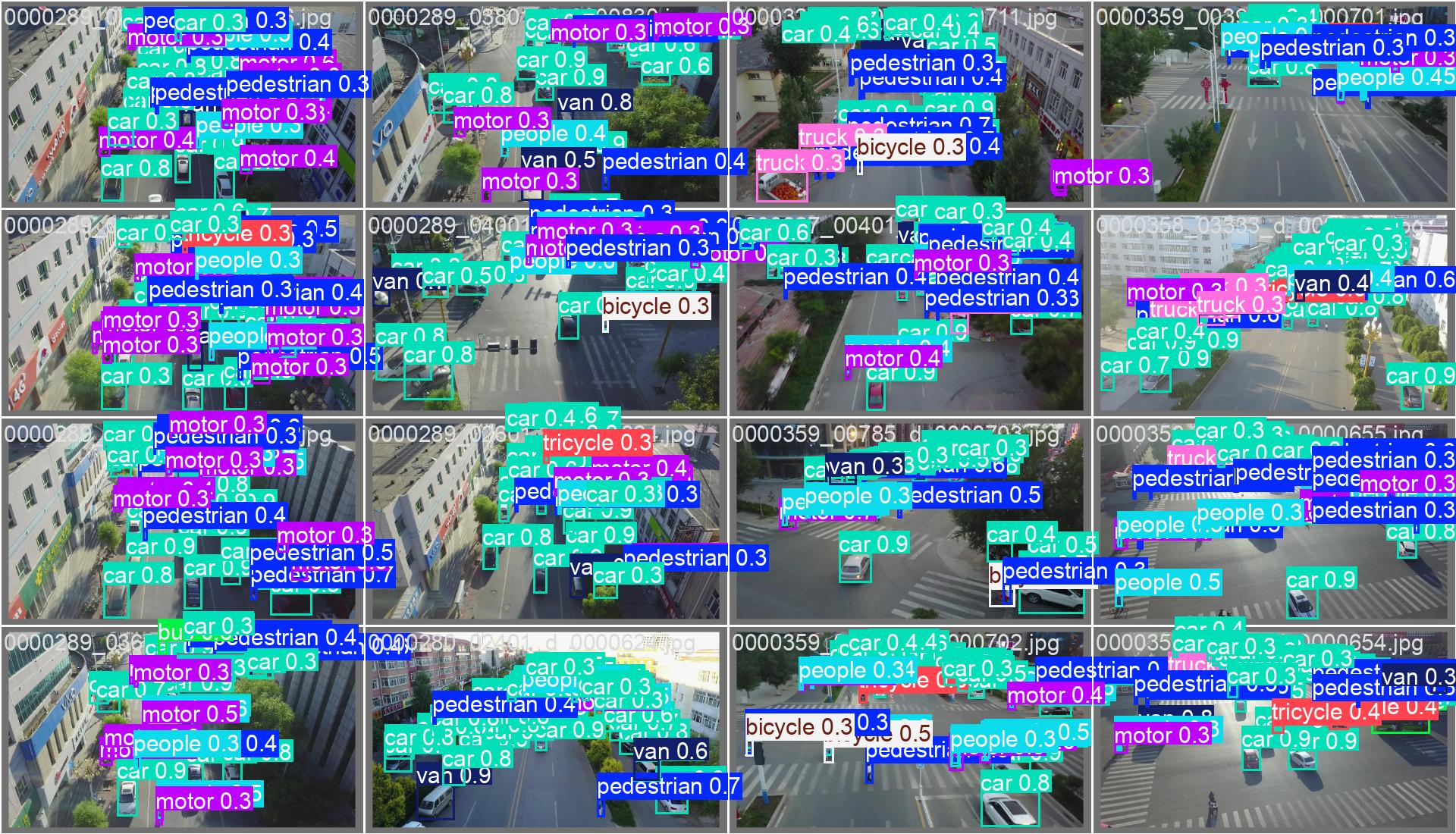

Supplement: S1 File — (ZIP) [file pone.0328248.s001.zip › S1 Model training result data/VisDrone/Train/yolo11n/val_batch2_pred.jpg]

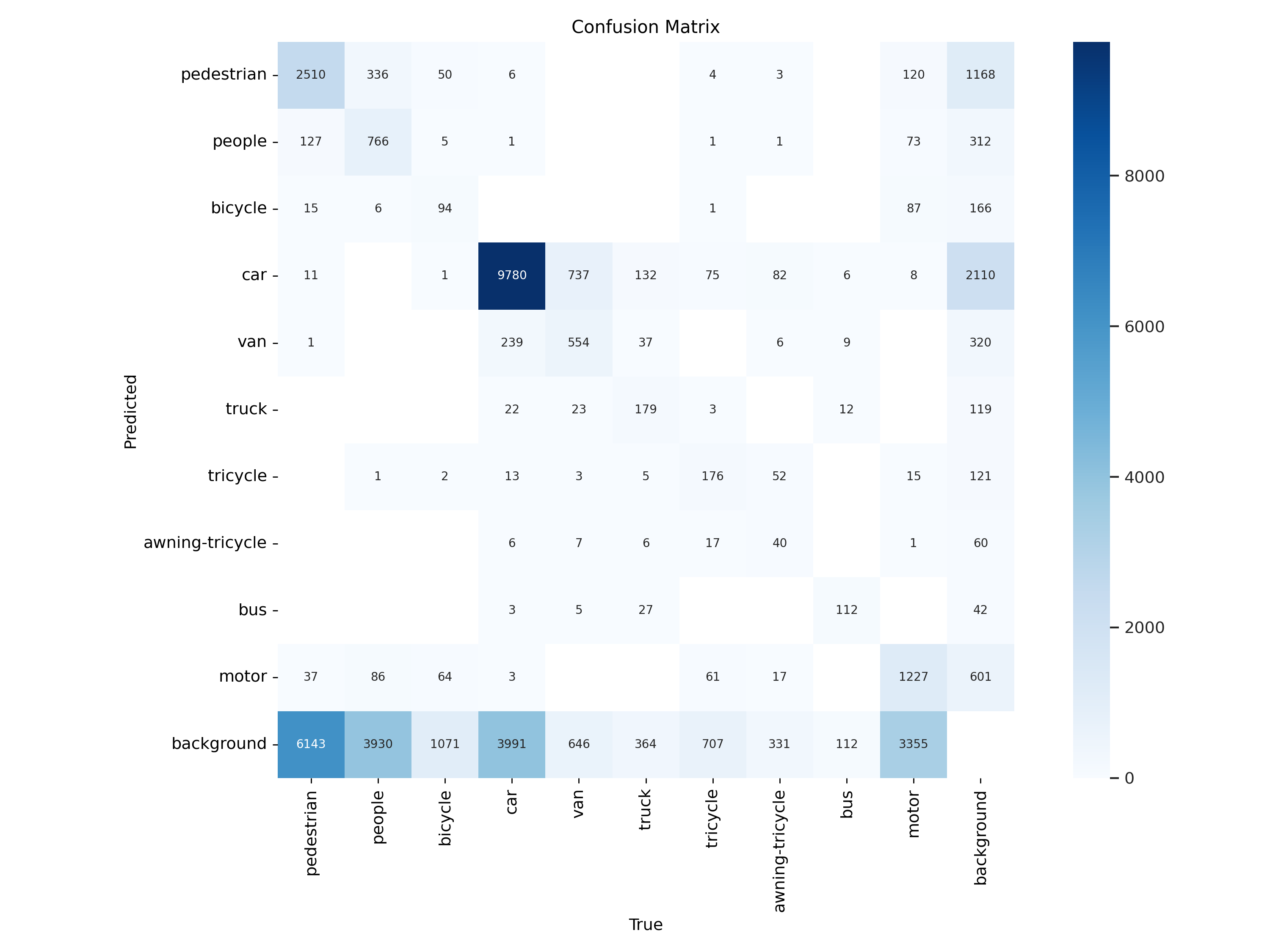

Supplement: S1 File — (ZIP) [file pone.0328248.s001.zip › S1 Model training result data/VisDrone/Train/yolov11+ours/confusion_matrix.png]

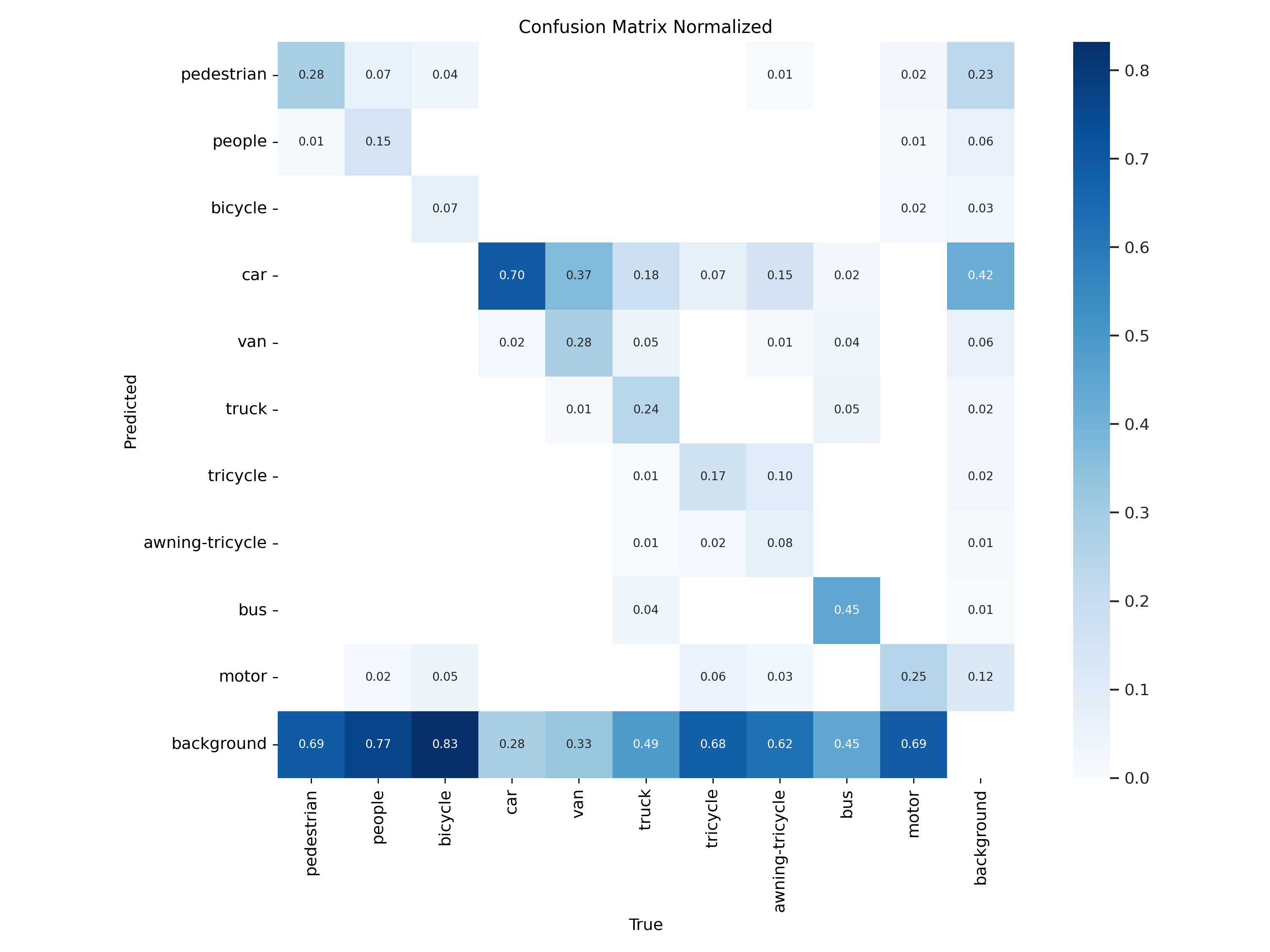

Supplement: S1 File — (ZIP) [file pone.0328248.s001.zip › S1 Model training result data/VisDrone/Train/yolov11+ours/confusion_matrix_normalized.png]

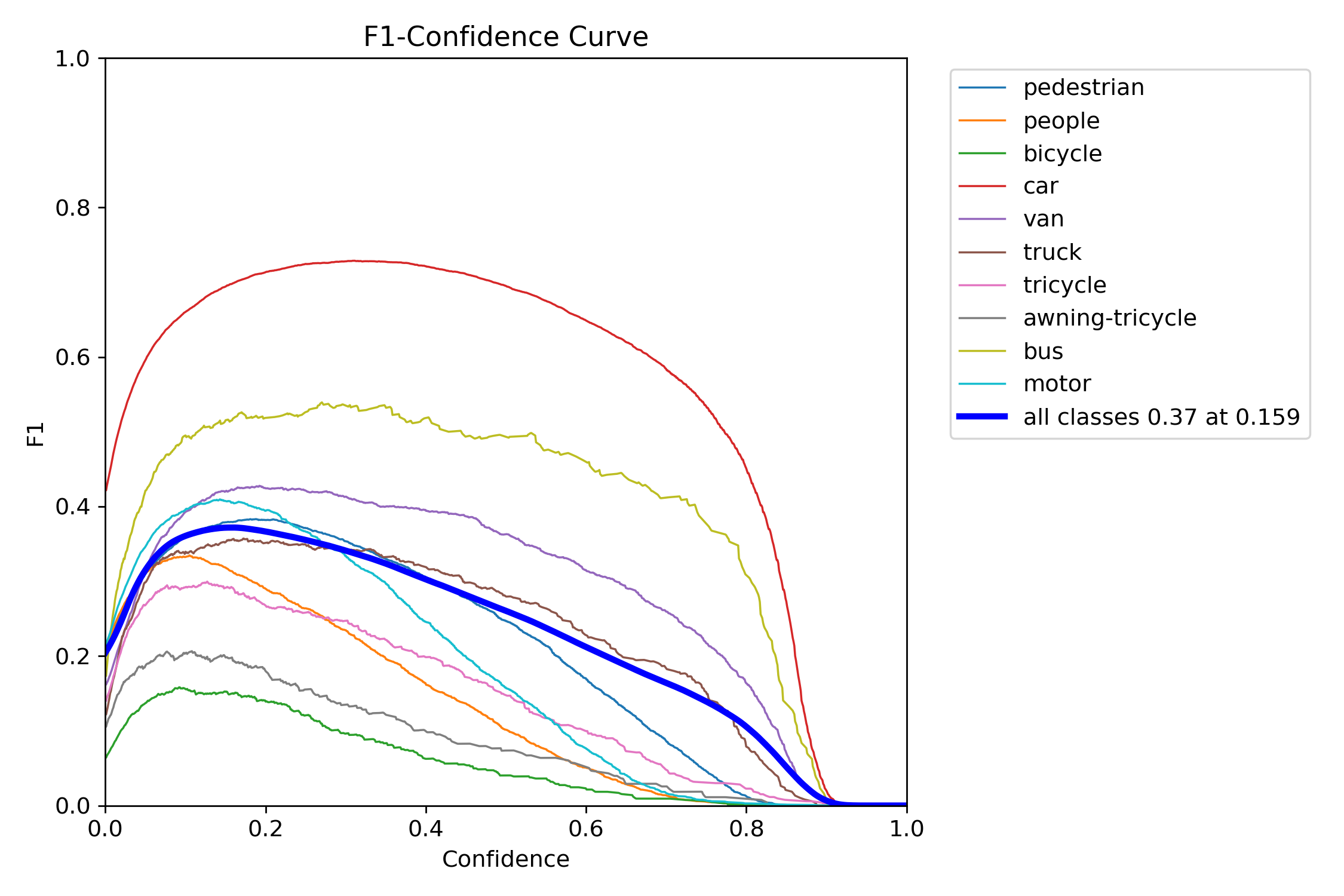

Supplement: S1 File — (ZIP) [file pone.0328248.s001.zip › S1 Model training result data/VisDrone/Train/yolov11+ours/F1_curve.png]

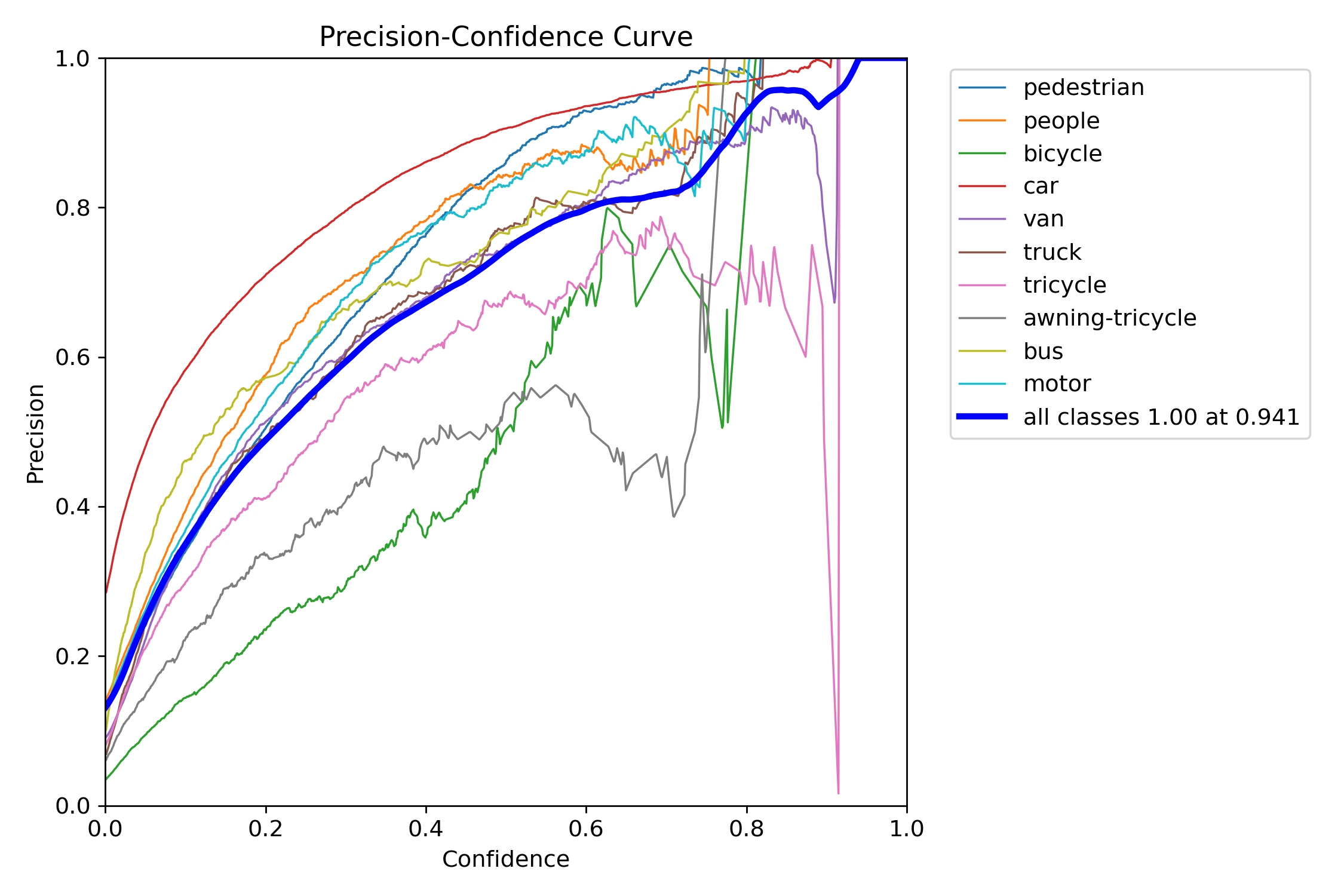

Supplement: S1 File — (ZIP) [file pone.0328248.s001.zip › S1 Model training result data/VisDrone/Train/yolov11+ours/P_curve.png]

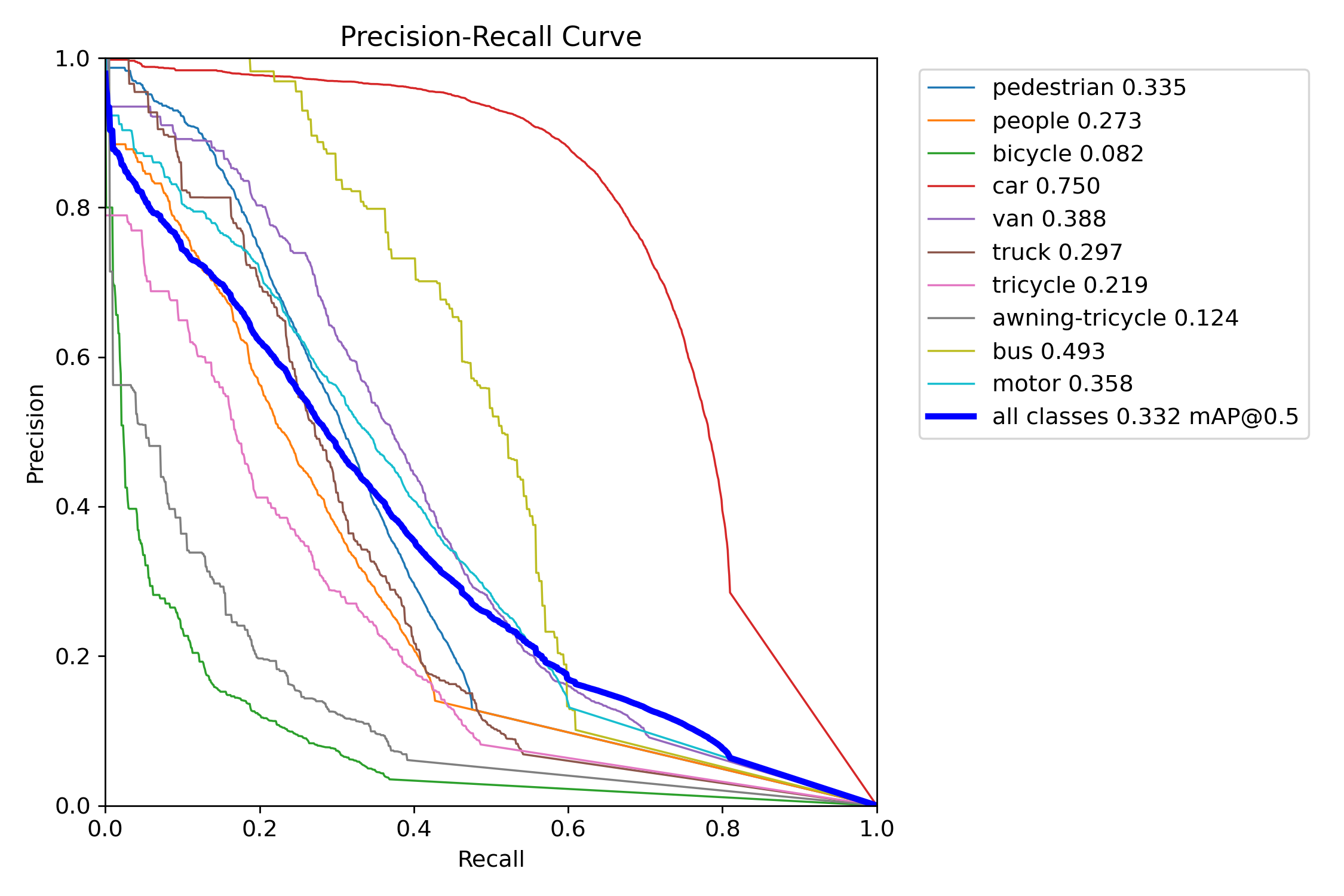

Supplement: S1 File — (ZIP) [file pone.0328248.s001.zip › S1 Model training result data/VisDrone/Train/yolov11+ours/PR_curve.png]

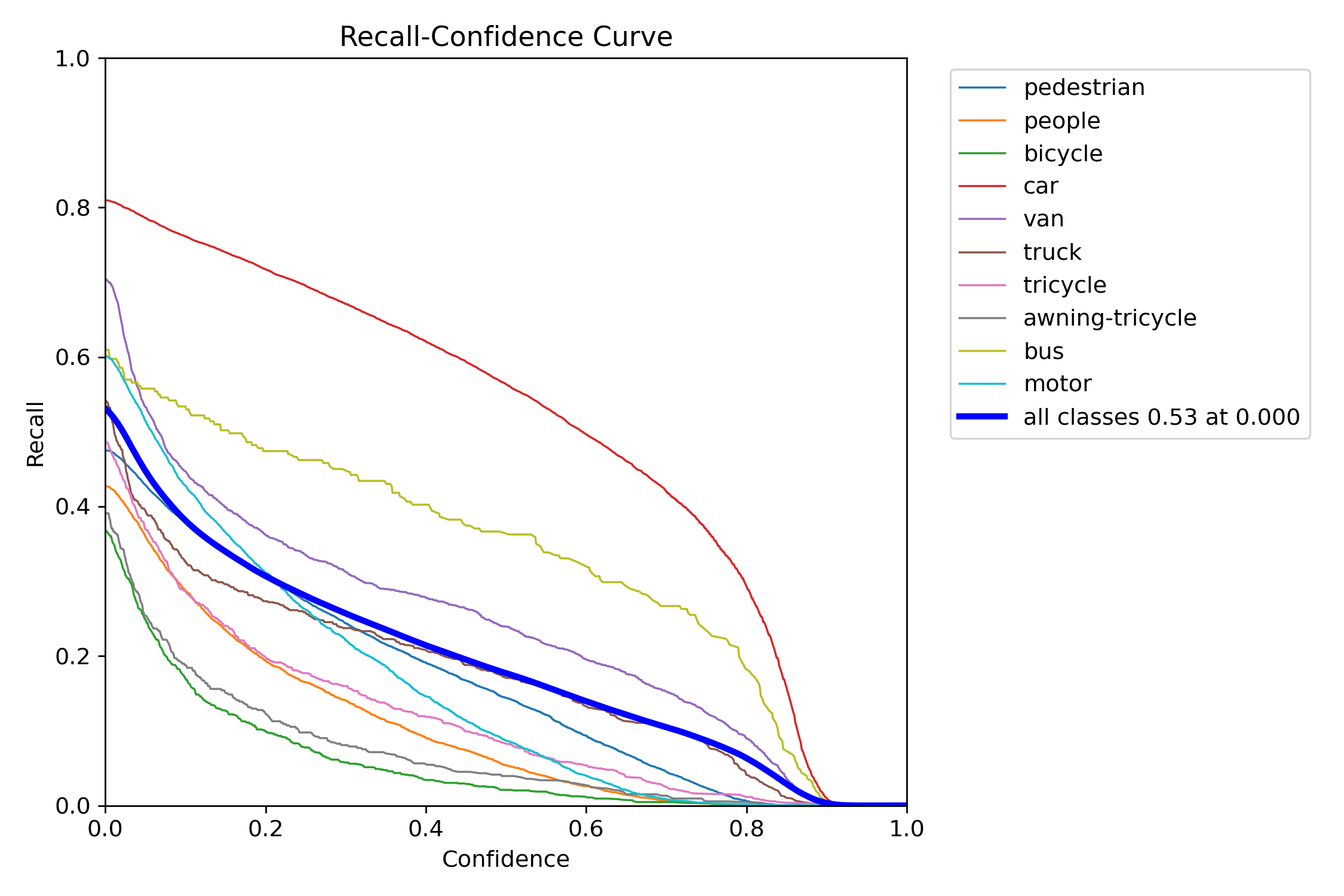

Supplement: S1 File — (ZIP) [file pone.0328248.s001.zip › S1 Model training result data/VisDrone/Train/yolov11+ours/R_curve.png]

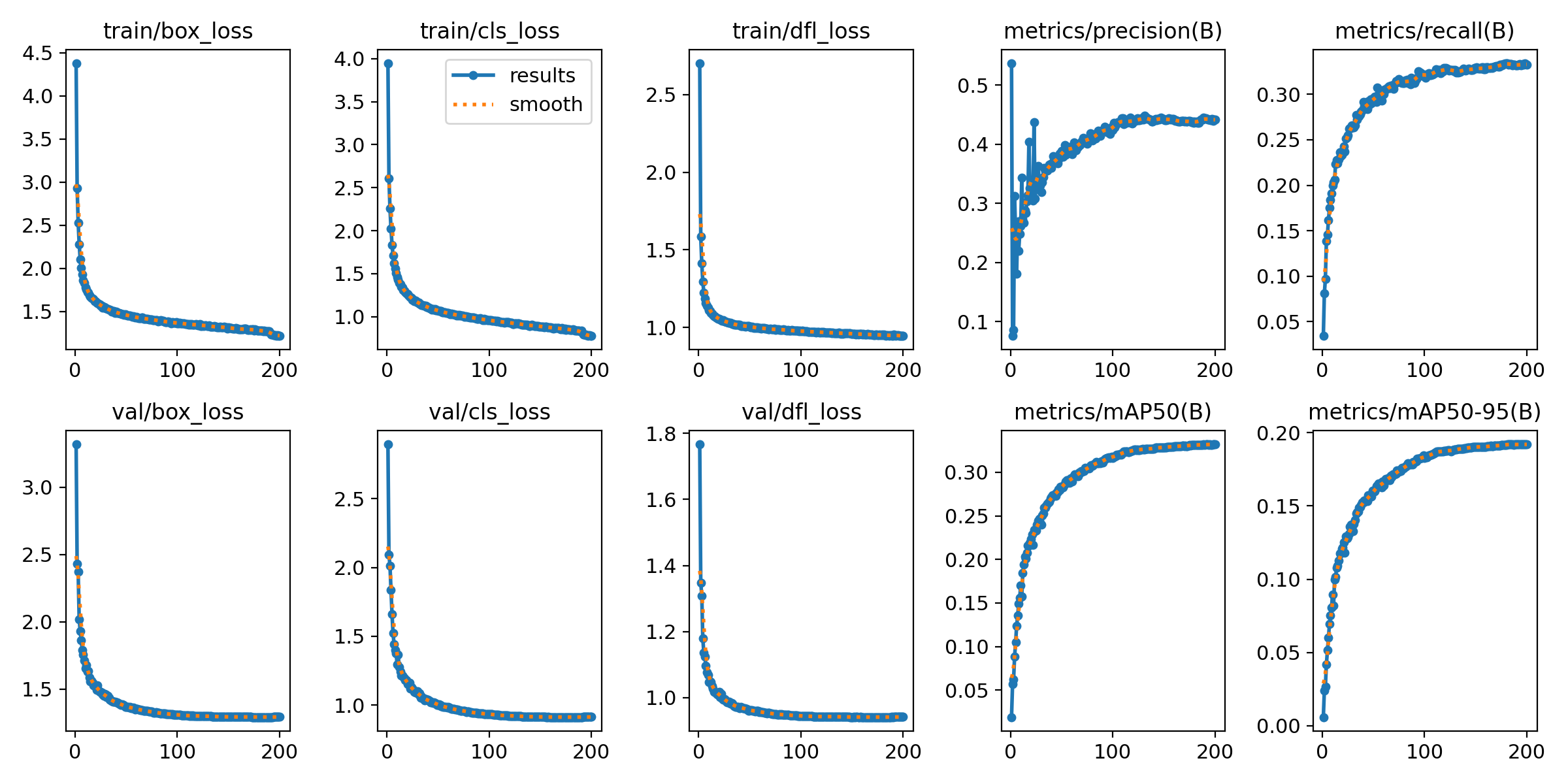

Supplement: S1 File — (ZIP) [file pone.0328248.s001.zip › S1 Model training result data/VisDrone/Train/yolov11+ours/results.png]

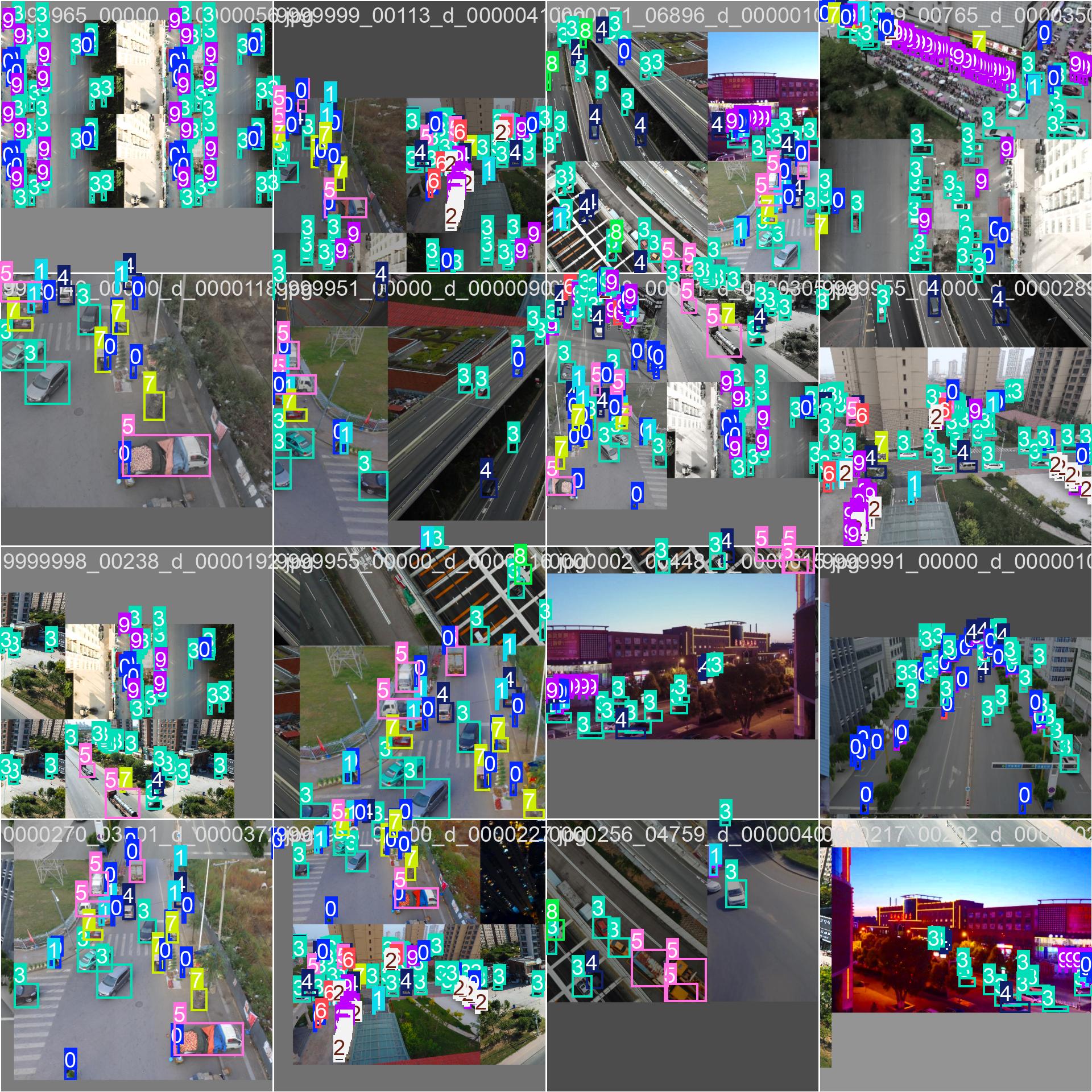

Supplement: S1 File — (ZIP) [file pone.0328248.s001.zip › S1 Model training result data/VisDrone/Train/yolov11+ours/train_batch0.jpg]

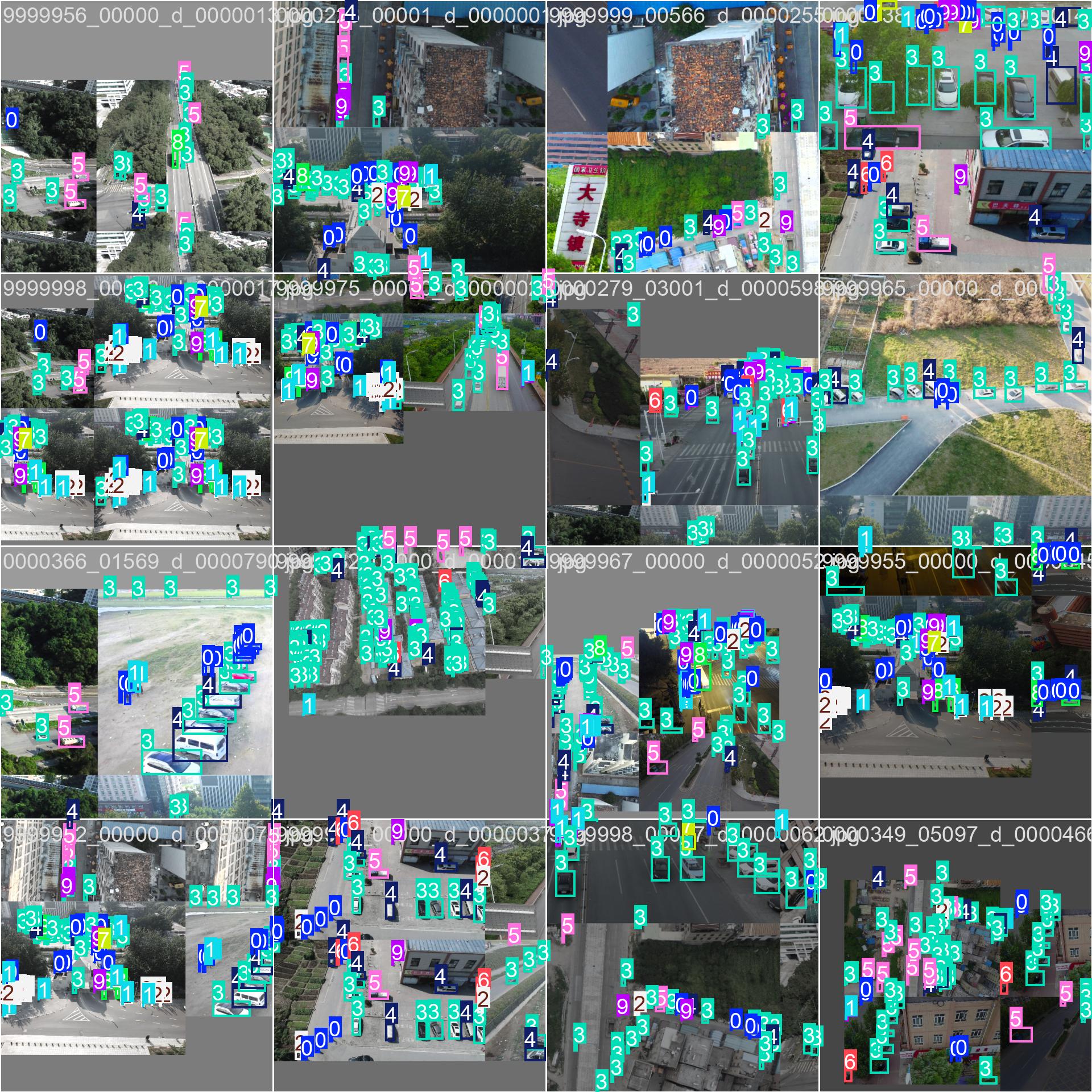

Supplement: S1 File — (ZIP) [file pone.0328248.s001.zip › S1 Model training result data/VisDrone/Train/yolov11+ours/train_batch1.jpg]

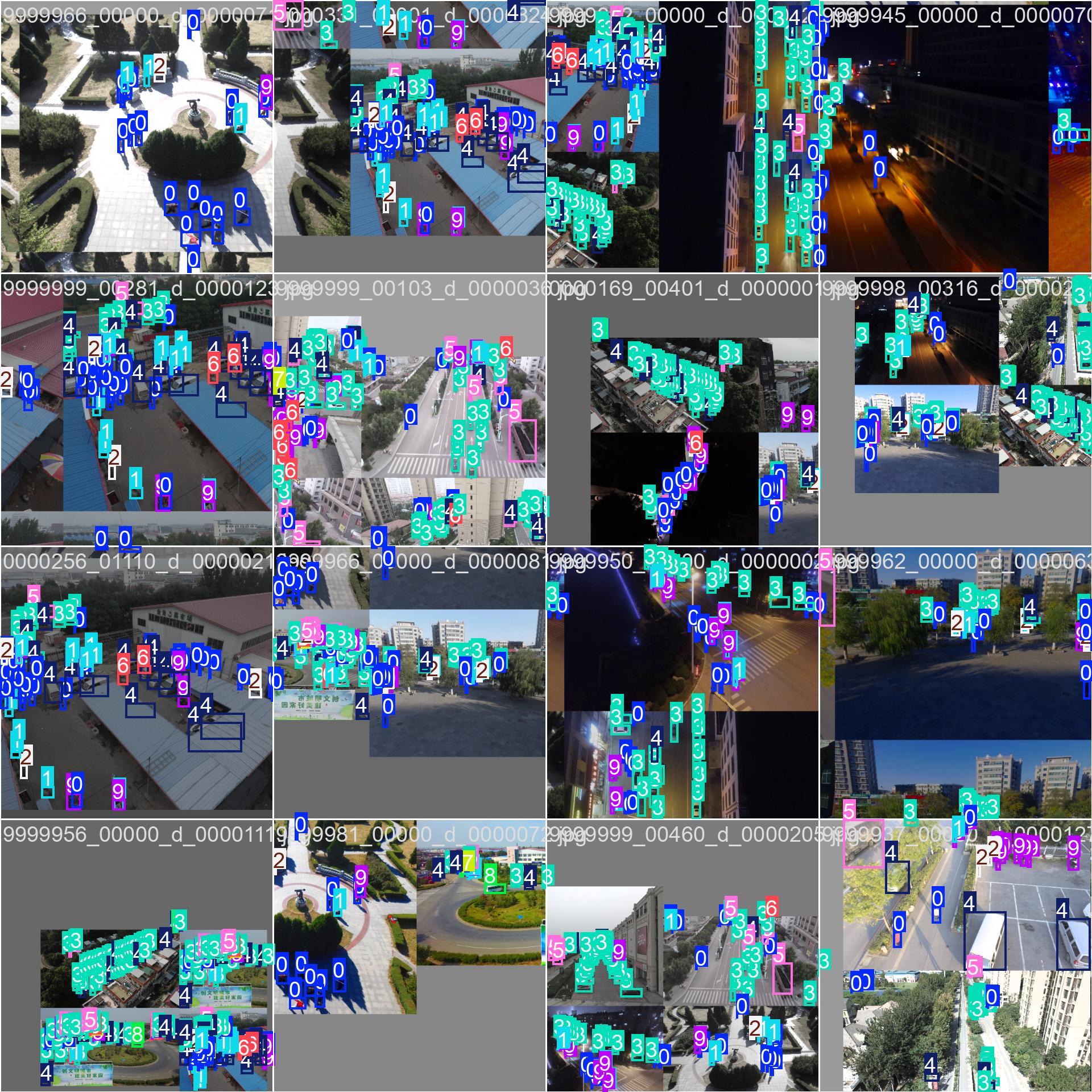

Supplement: S1 File — (ZIP) [file pone.0328248.s001.zip › S1 Model training result data/VisDrone/Train/yolov11+ours/train_batch2.jpg]

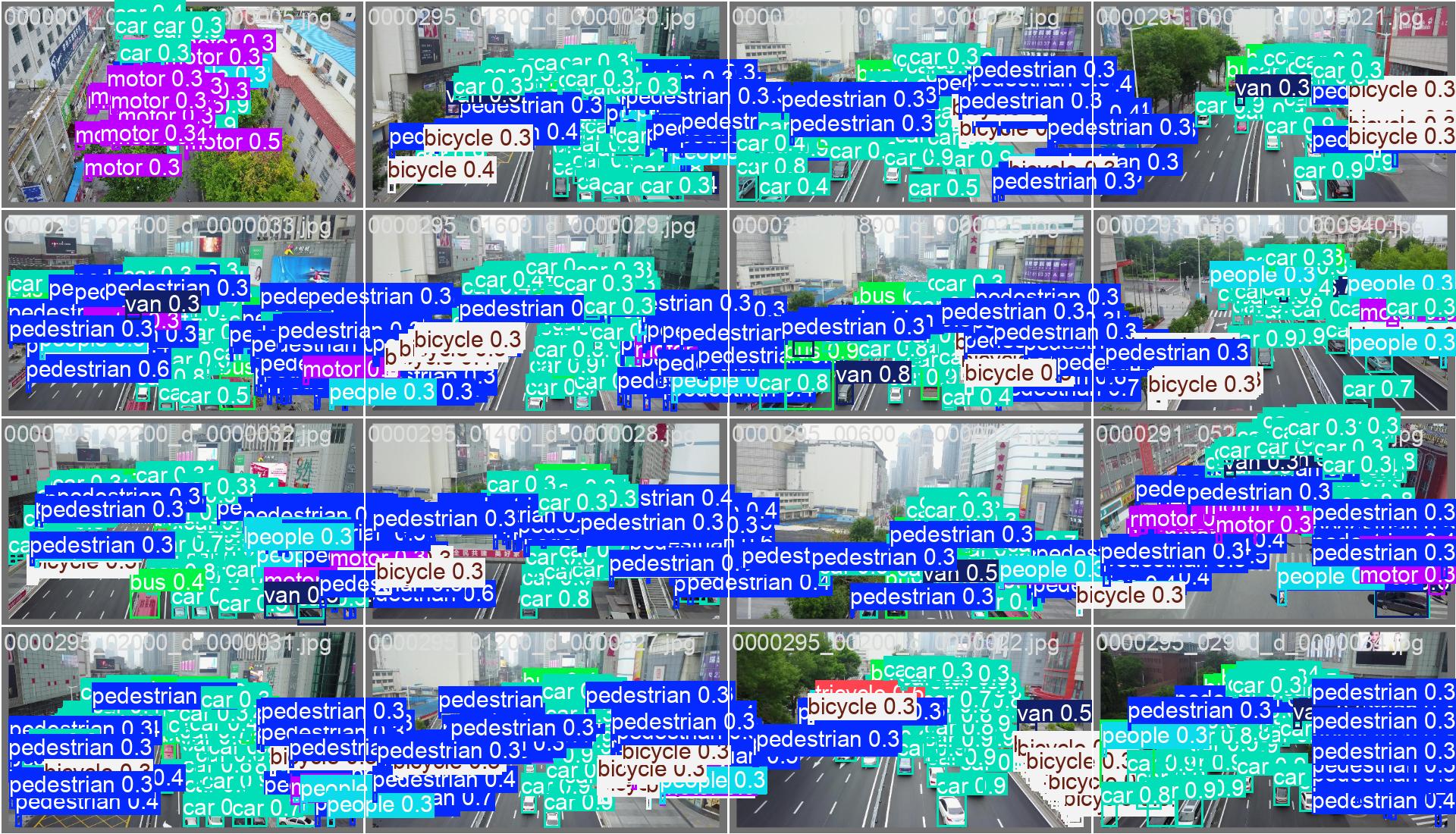

Supplement: S1 File — (ZIP) [file pone.0328248.s001.zip › S1 Model training result data/VisDrone/Train/yolov11+ours/val_batch0_pred.jpg]

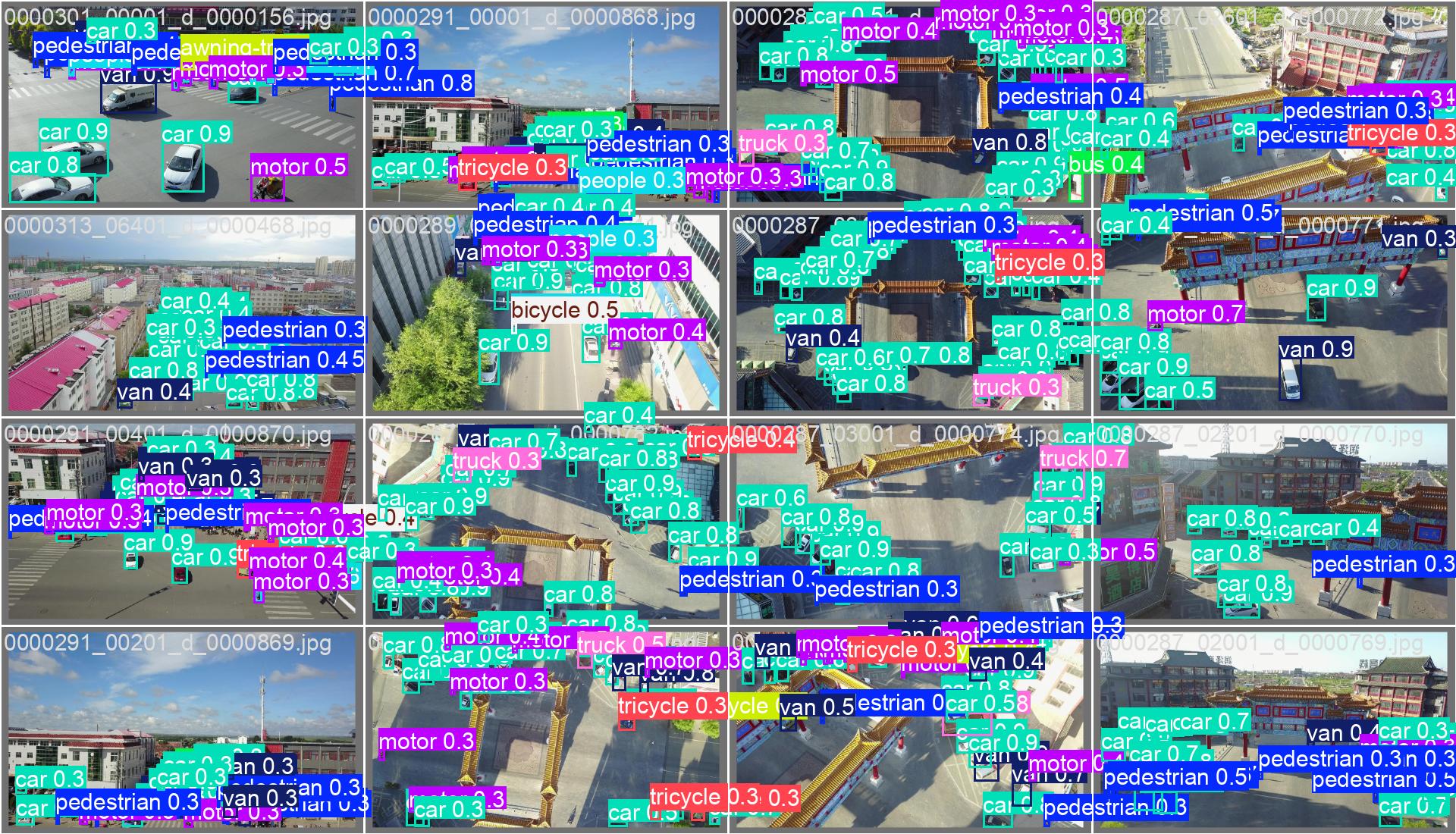

Supplement: S1 File — (ZIP) [file pone.0328248.s001.zip › S1 Model training result data/VisDrone/Train/yolov11+ours/val_batch1_pred.jpg]

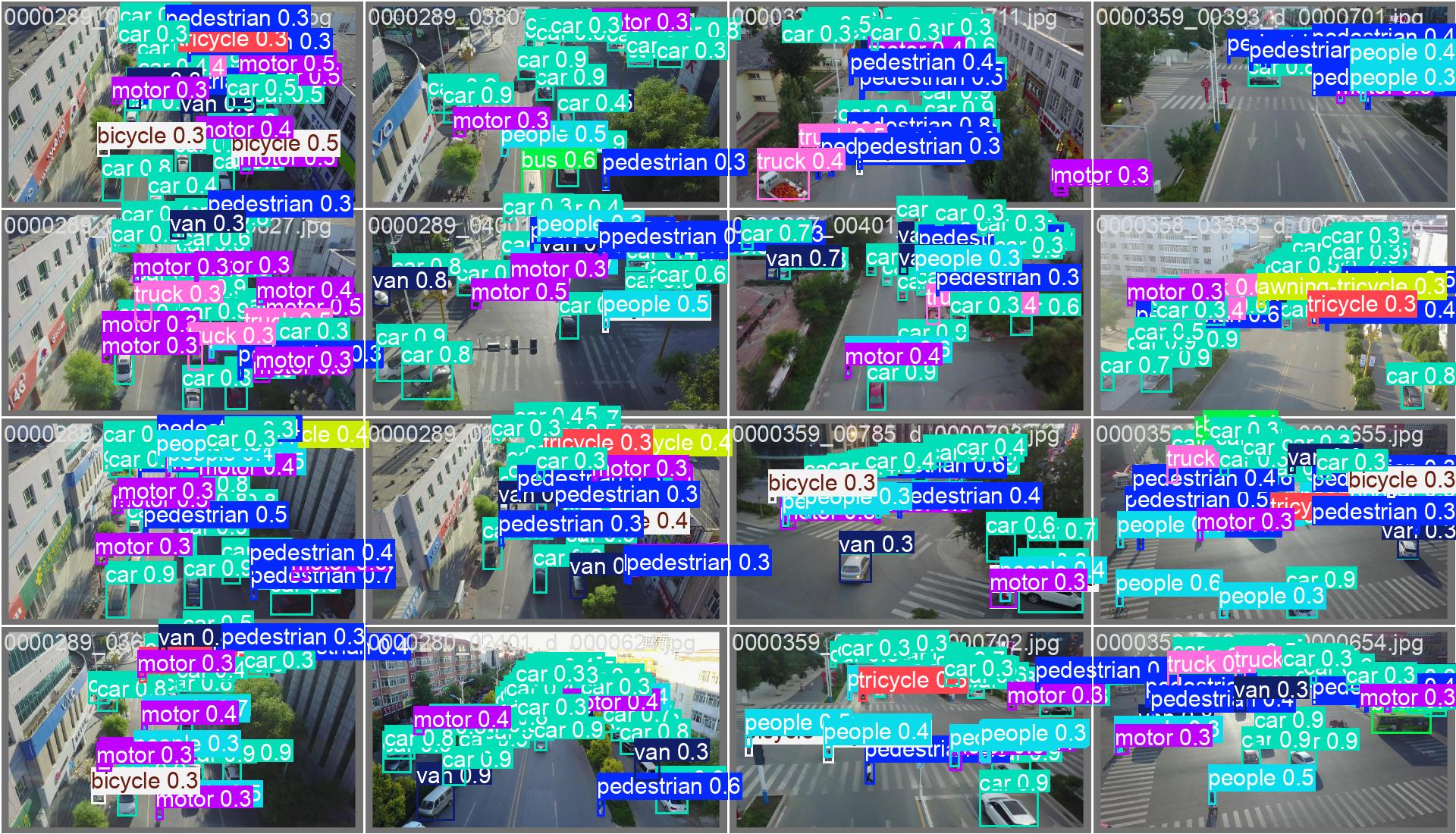

Supplement: S1 File — (ZIP) [file pone.0328248.s001.zip › S1 Model training result data/VisDrone/Train/yolov11+ours/val_batch2_pred.jpg]
