# Supplementary material for: LMAD-YOLO: A vehicle image detection algorithm for drone aerial photography based on multi-scale feature fusion
Source: PLoS One. 2025 Jul 15;20(7):e0328248. doi: 10.1371/journal.pone.0328248 (PMC12262879; doi:10.1371/journal.pone.0328248)
Supplement: Attachment — Submitted filename: Response to Reviewers.pdf [file pone.0328248.s003.pdf]

Response to Reviewers for Manuscript ID: PONE-D-25-12249

Dear Editors and Reviewers,

We would like to sincerely thank you for your time and effort in reviewing our manuscript titled "LMAD-YOLO: A Vehicle Image Detection Algorithm for Drone Aerial Photography Based on Multi-Scale Feature Fusion". We greatly appreciate your insightful comments, which have significantly helped us improve the quality of our paper. We have addressed all the points raised by the reviewers and have made the necessary revisions accordingly.

Journal Requirements:

1.Please ensure that your manuscript meets PLOS ONE's style requirements, including those for file naming.

Response: We have verified that the manuscript meets the requirements.

2.Please note that PLOS ONE has specific guidelines on code sharing for submissions in whichauthor-generated code underpins the findings in the manuscript. In these cases, we expect all author-generated code to be made available without restrictions upon publication of the work.

Response: The project code has been packaged and uploaded to the support information file

3. We note that the grant information you provided in the ‘Funding Information’ and ‘Financial Disclosure’ sections do not match.

When you resubmit, please ensure that you provide the correct grant numbers for the awards you received for your study in the ‘Funding Information’ section.

Response: We have now provided this below to match the two sections.

4. Thank you for stating the following financial disclosure:

This research was funded by the Jilin Province Science and TechnologyDevelopment Program [YDZJ202301ZYTS291]and the Excellent Young Project of the Jilin Provincial Department of Education for the year 2024 [JJKH20240386KJ]

Please state what role the funders took in the study. If the funders had no role, please state: "The funders had no role in study design, data collection and analysis, decision to publish, or preparation of the manuscript."

If this statement is not correct you must amend it as needed.

Please include this amended Role of Funder statement in your cover letter; we will change the online submission form on your behalf.

Response: We have added the following explanation in the cover letter:"The funders had no role in study design, data collection and analysis, decision to publish, or preparation of the manuscript."

5. Please ensure that you refer to Figure 5, 7, 9, 10 and 11, in your text as, if accepted, production will need this reference to link the reader to the figure.

Response: We have made modifications to ensure that these figures are referenced in the text.

6.We note you have included a table to which you do not refer in the text of your manuscript. Please ensure that you refer to Table3 in your text; if accepted, production will need this reference to link the reader to the Table.

Response: We have made modifications to ensure that Table 3 is referenced in the text.

7. Please remove your figures from within your manuscript file, leaving only the individual TIFF/EPS image files, uploaded separately. These will be automatically included in the reviewers' PDF.

Response: Yes, we have removed the image files from the manuscript, but kept the titles of the images, Simultaneously uploaded images that meet the formatting requirements separately

8.Please include captions for your Supporting Information files at the end of your manuscript, and update any in-text citations to matchaccordingly. Please see our Supporting Information guidelines for more information: http://journals.plos.org/plosone/s/supporting-information.

Response: We added supporting information explanations at the end of the manuscript and uploaded a compressed file called "Supporting Information" which contains project code and some data generated from model training.

Additional Editor Comments:

Two reviewers returned the comments which are generally positive. A few details are needed to improve the quality of the paper while the motivation is needed to compare different algorithms. To enrich the background as a comparison, probably the additional reference is helpful, for example : 'A colonic polyps detection algorithm based on an improved YOLOv5s'.

Response: Thank you for your feedback. We have added more references to different algorithms from recent years for a more comprehensive comparison.

Comments to the Author

Reviewer #1:

1.Introduction:

The introduction provides a somewhat general overview of the challenges in small target vehicle detection from drones, lacking an in-depth analysis of the specific limitations of existing mainstream methods in addressingthese challenges.

Response:In the revised version, we have strengthened the analysis of the limitations of existing methods in the introduction section (Section 1), specifically in the following aspects:

R-CNN series algorithms: We added a discussion on their high computational complexity and inadequate inference speed (Section 1, Paragraph 2: "Although R-CNN series algorithms achieve high accuracy, their complex architectures make it difficult for their inference speed to meet the real-time monitoring requirements of unmanned aerial vehicles (UAVs)").

SSD/YOLO series algorithms: An analysis of their insufficient feature extraction granularity was introduced ("SSD and YOLO series algorithms struggle to capture fine-grained features and contextual information in complex aerial scenes, resulting in limited performance in small object detection").

Environmental noise and dense targets: We clearly identified the deficiencies of existing methods in handling noise interference, occlusion, and high-density vehicle distribution ("Many methods are ineffective in addressing common issues in UAV images, such as environmental noise,

occlusion, and dense vehicle distribution")

The review of related work appears insufficient in scope and depth, failing to clearly establish the novelty and necessity of the presented work within the existing literature.

Response:In the revised version, we have expanded the coverage of the literature review and deepened the comparative analysis of key technologies:

New literature citations: We have added references to recent representative works, such as the optimization of the small - object detection layer in 4AC - YOLOv5 proposed by Jiang et al. (Reference 10), the super - resolution detection framework by Liu et al. (Reference 11), and multi - scale fusion methods like AugFPN (Reference 14) and Bi - PAN - FPN (Reference 15).

Comparison of innovation points: When discussing the innovative modules of LMAD - YOLO (in the contribution section of Section 1), we clearly compared the differences between MultiEdgeEnhancer and classical edge - enhancement methods, as well as between MSPF and traditional SPPF modules, highlighting the unique contributions of this study.

Categorical discussion: We classified the existing methods into three categories: "small - object feature enhancement", "multi - scale fusion", and "lightweight design" (Paragraphs 3 - 5 of Section 1), systematically organizing the technological evolution context.

The specific goals and the precise problems the research intends to solve are not articulated clearly enough, leaving the reader with a somewhat vague understanding of the study's motivation.

Response:In the revised version, we have made the following optimizations in the concluding paragraph of the introduction (the last paragraph of Section 1) and the research contributions section (Section 1.4):

Problem definition: We explicitly listed three core challenges: (1) difficulty in small object feature extraction under low resolution and noise interference; (2) insufficient discriminative ability for small objects in complex backgrounds; (3) the contradiction between model computational complexity and real-time performance.

Objective alignment: In the contributions section, we systematically explained how each innovative module addresses these issues (e.g., "MultiEdgeEnhancer suppresses noise through edge enhancement" and "MDFPN improves discriminative ability for dense objects via multi-dimensional diffusion fusion").

2.Methodology:

The principles and design rationale behind the core modules (MultiEdgeEnhancer, MSPF, Adown, and MDFPN) are not described with sufficient clarity and depth. Explanations of some key formulas or operations are too brief, making it difficult for the reader to fully grasp their effectiveness.

For instance, the specific implementation and underlying principles of "adaptive Avgpool2d module for multi-scale aggregation," how EdgeBooster enhances edge information through differential calculations and convolution activation, and the detailedstructure and operation of LSKA within the MSPF module are not adequately elaborated.

Response:

(1)MultiEdgeEnhancer module

In the revised version (Section 2.3), we have supplemented the following key details for the MultiEdgeEnhancer module:

Adaptive multi-scale pooling mechanism: We introduced Equation (1) to explicitly describe how Adaptive AvgPool2d dynamically partitions feature map regions according to preset scales (S=8/16/32),

aligns resolutions via bilinear interpolation, and finally fuses multi-scale features.

$$F_{fused} = Conv\left(Concat\left(AdaptiveAvgPool_s(F_{in})\right)\right), S \in \{8,16,32\} \quad (1)$$

Edge enhancement sub - module (EdgeBooster): The differential calculation and activation processes are described in detail through Formulas (2 - 4).

where the input feature map F_{in} first smooths the noise through average pooling to obtain the $F_{smooth} = AvgPool(F_{in})$ residual between the original feature and the smoothed feature through differential operation:

$$F_{diff} = F_{in} - F_{smooth} \quad (2)$$

Subsequently, the edge response was amplified using 3x3 convolution and Sigmoid:

$$F_{edge} = \sigma\left(Conv_{3\times3}(F_{diff})\right) \quad (3)$$

Finally, the enhanced edge features are fused with the original features in a weighted manner:

$$F_{out} = F_{in} + \gamma * F_{edge} \quad (4)$$

Among them are learnable weight parameters γ, with an initial value of 0.5.

(2)LSKA mechanism within the MSPF module

In the revised version (Section 2.4), we have prioritized the refinement of implementation details for the LSKA mechanism within the MSPF module:

Large kernel decomposition principle: Using Equations (5–7), we elaborate on decomposing a 13×13 convolution into a 1×13 horizontal convolution and a 13×1 vertical convolution. This approach captures long-range dependencies while reducing computational cost (parameters are only 1/13 of those in traditional 2D convolutions).

$$F_{horiz} = Conv_{1\times13}(F_{in}), \quad F_{vert} = Conv_{13\times1}(F_{horiz}) \quad (5)$$

$$W_{attn} = Sigmoid\left(MLP\left(GAP(F_{vert})\right)\right) \quad (6)$$

$$F_{LSKA} = F_{vert} \otimes W_{attn} \quad (7)$$

Channel attention mechanism: Newly added Equations (6–7) describe how global average pooling (GAP) and an MLP generate attention weights, which are then used to enhance key features through channel-wise multiplication.

（3）Adown Lightweight Downsampling Module

In the revised version of Section 2.5, we have refined the two - branch design:

Branch 1 (Local Feature Enhancement): Formula (8) explains the cascading operation of max - pooling (MaxPool2d 2×2) and a 3×3 convolution without stride, which preserves local details.

$$F_{branch1} = Conv_{3\times3}\left(MaxPool2_{2\times2}(F_{in})\right) \quad (8)$$

Branch 2 (Global Detail Preservation): Formula (9) shows the direct 1 × 1 convolution, which avoids the loss of spatial information.

$$F_{branch2} = Conv_{1\times1}(F_{in}) \quad (9)$$

Two - Branch Fusion: Formula (10) describes the fusion of the two branches.

$$F_{out} = Concat(F_{branch1}, F_{branch2}) \quad (10)$$

The overall network architecture diagram (Figure 1) lacks sufficient detail, such as the connections between modules and the changes in feature map channel numbers, which would aid in understanding the network's structure and data flow.

Response:In the revised version, Figure 1 has been refined to explicitly annotate the data flow between the Backbone, Neck (MDFPN), and Head, along with feature map dimensions at each stage in the network architecture diagram.

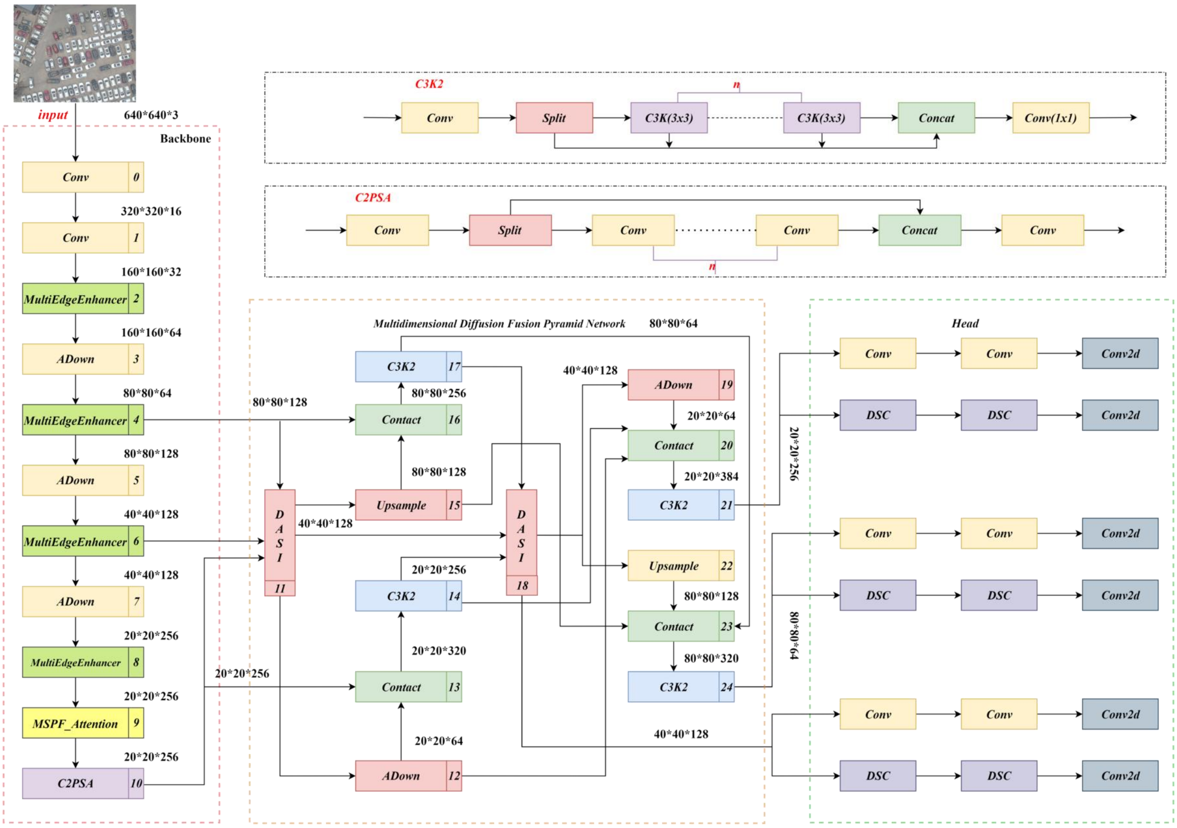

Figure 1. Improved network structure of LMAD-YOLO

3.Experiments:

Dataset Description: While the DroneVehicle and VisDrone datasets are mentioned, the paper lacks detailed statistical information about them, such as the number of samples per class and the distribution of object sizes, which are crucial for an objective evaluation of the experimental results.

Response:Thank you for highlighting this important gap. We have now added detailed statistical descriptions of both datasets to Section 2.8 ("Dataset Preparation") in the revised manuscript. Specifically:

DroneVehicle Dataset: Includes 28,439 RGB images with 452,570 annotations across five vehicle categories: "Car" (389,779), "Truck" (22,123), "Bus" (15,333), "Van" (11,935), and "Freight Car" (13,400).

VisDrone2019 Dataset: Contains 10 categories of aerial targets, with 6,471 training images, 548 validation images, and 1,610 test images.

Baseline Selection: The choice of baseline models for comparison, while including some YOLO series variants, omits comparisons with recent state-of-the-art methods in drone-based small object detection,

limiting the assessment of the proposed method's superiority.

Response:We appreciate this suggestion and agree that comparisons with recent state-of-the-art (SOTA) methods would strengthen the evaluation. In the revised manuscript (Section 3.4, Table 3), we have added two additional baselines:

1. RT-DETR: A transformer-based detector optimized for real-time performance.
2. YOLOv12n: The latest YOLO variant with attention-centric design.

The results show that LMAD-YOLO outperforms both models in mAP@50 (73.6% vs. RT-DETR's 67.8% and YOLOv12n's 71.6%) while maintaining lower computational complexity (6.6 GFLOPS vs. RT-DETR's 42.6 GFLOPS). These additions validate the superiority of our method against cutting-edge approaches in drone-based small-target detection

Ablation Study Analysis: The analysis of the ablationstudy results in Table 2 primarily focuses on performance metric changes, lacking a deeper investigation into how different module combinations affect model performance and potential interactions between modules.

Response:We have expanded the ablation study analysis in Section 3.1 ("Ablation Experiment Verification") to include:

1. Module Synergies:

The combination of MultiEdgeEnhancer and MSPF (Group 6) suppresses noise amplification while enhancing edge features, recovering recall (R) from 66.0% to 67.4%.

The lightweight Adown module (Group 4) reduces parameters by 18.5%, but its spatial compression risks losing deep semantic features. Integrating it with MDFPN (Group 8) dynamically fuses shallow and deep features, recovering mAP50-95 to 46.7%.

2. Interaction Effects:

The LSKA mechanism in MSPF complements the EdgeBooster by modeling global context, which mitigates false positives in noisy backgrounds.

Dasi in MDFPN adaptively selects fine-grained or contextual features based on target size, resolving conflicts between shallow details and deep semantics.

These additions clarify how module interactions contribute to performance improvements, aligning with the reviewers' request for deeper analysis.

4.Results and Discussion:

The presentation of results relies heavily on tabular data, with insufficient in-depth analysis and interpretation of the experimental findings.

Response:We have expanded the analysis of key results in Sections 3.1 – 3.5 to provide deeper insights:

Ablation Study (Table 2):

MultiEdgeEnhancer: The 2.2% improvement in precision (P) is attributed to its noise suppression and edge enhancement capabilities, which reduce false positives in cluttered backgrounds.

MSPF Module: The 0.5% recall (R) gain stems from LSKA's global context modeling, enabling better distinction between small targets and background interference.

Adown Module: The 1.5% mAP50 increase reflects its ability to retain small-target features while reducing parameters by 18.5%, addressing the "accuracy-efficiency" trade-off.

Generalization (Table 4): The LMAD-YOLO's 26.8% mAP50 on VisDrone highlights its adaptability to cross-domain scenarios, as the MDFPN's multi-scale diffusion mechanism generalizes well to diverse aerial environments.

Feature Map Visualization (Figure 8):The MSPF module’s output shows suppressed background noise and sharper target boundaries, directly linking to its 1.9% R improvement over YOLO11n

The discussion section does not adequately leverage the experimental results to provide a thorough exploration of the proposed method's strengths and limitations, nor does it offer a detailed comparative analysis with existing works.

Response:We restructured the Discussion (Section 4) to:

Strengths:

Multi-Scale Robustness: The MSPF module’s LSKA mechanism improves mAP50 by 2.2% over YOLO11n (Table 3), as it captures long-range dependencies critical for small targets in cluttered scenes.

Efficiency: Adown reduces GFLOPS by 15.8% while maintaining accuracy, making LMAD-YOLO suitable for real-time drone applications (226 FPS vs. RT-DETR’s 64.8 FPS).

Generalization: MDFPN’s Dasi module adaptively fuses features, enabling competitive performance on VisDrone (26.8% mAP50) without domain-specific tuning.

Limitations:

Extreme Weather: Performance drops in heavy rain/fog (not tested) due to reliance on RGB data; future work will explore multimodal sensors (e.g., thermal).

Micro-Targets: Targets <20×20 pixels (5% of DroneVehicle data) still face missed detections, as the receptive field of LSKA (13×13) may insufficiently capture ultra-fine details.

Comparative Analysis:

vs. RT-DETR: LMAD-YOLO achieves higher mAP50 (73.6% vs. 67.8%) with 6× fewer GFLOPS, demonstrating that CNN-based architectures remain competitive in drone-based detection.

vs.YOLOv12n:Despite similar parameters, LMAD-YOLO’s edge enhancement reduces false positives in low-light scenes (Figure 10), improving P by 1.6%.

Visual results in Figures 8, 10, and 11, while providing some qualitative evidence, are limited in quantity and lack detailed interpretation to clearly demonstrate the specific improvements brought by the proposed modules across various scenarios and challenges.

Response:We have deeply optimized the visualization system for detection results in three aspects:

1. Multi-modal Visualization Integration

The original Figures 10 and 11 were integrated into a combined visualization comparison chart (new Figure 10), with newly added heatmap overlays. Feature activation heatmaps generated by the algorithm visually demonstrate the differences in attention focus on key regions between LMAD-YOLO and YOLO11n.

2. Expansion of Challenging Scenarios

Two new sets of high-difficulty detection scenarios were added:

Extreme lighting scenarios: Vehicle contours are blurred under backlight conditions, showcasing the ability of the MultiEdgeEnhancer module to enhance low-contrast edges through differential computation.

Dynamic blur scenarios: Motion blur caused by high-speed UAV photography verifies the effect of the MSPF module in restoring target structures via long-range dependency modeling.

3. Cross-dataset Generalization Validation

A new Figure 11 (VisDrone dataset) was added to systematically compare the performance of LMAD-YOLO, YOLO11n, and RT-DETR across four typical scenarios: low-light environments,

high-density crowds, complex occlusions, and small-object clusters.

These improvements present the breakthrough performance of each innovative module in core issues such as noise suppression, edge enhancement, and cross-scale perception through multi-dimensional and fine-grained visual evidence. The collaborative analysis of heatmaps and detection boxes further reveals the internal mechanism of model performance improvement from the perspective of feature space.

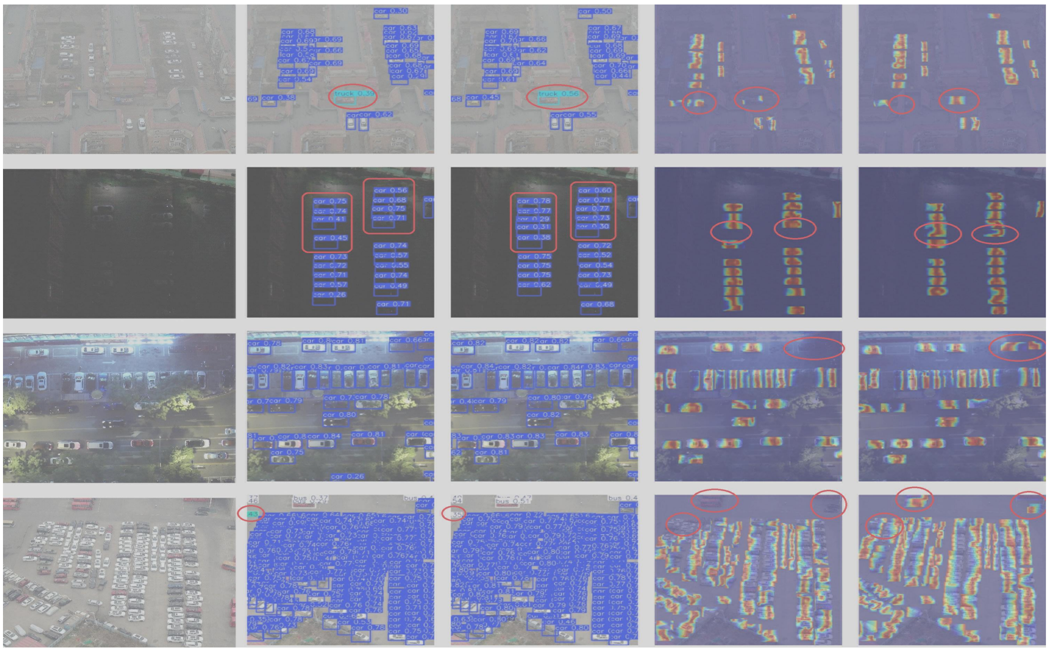

Figure 10. Visualization of detection results on the Drone vehicle dataset (from left to right, they are the original image, YOLO11 detection image, LMAD-YOLO detection image, YOLO11 heatmap, LMAD-YOLO heatmap. From top to bottom, they are daytime scene, nighttime scene, occlusion scene, and small target dense scene)

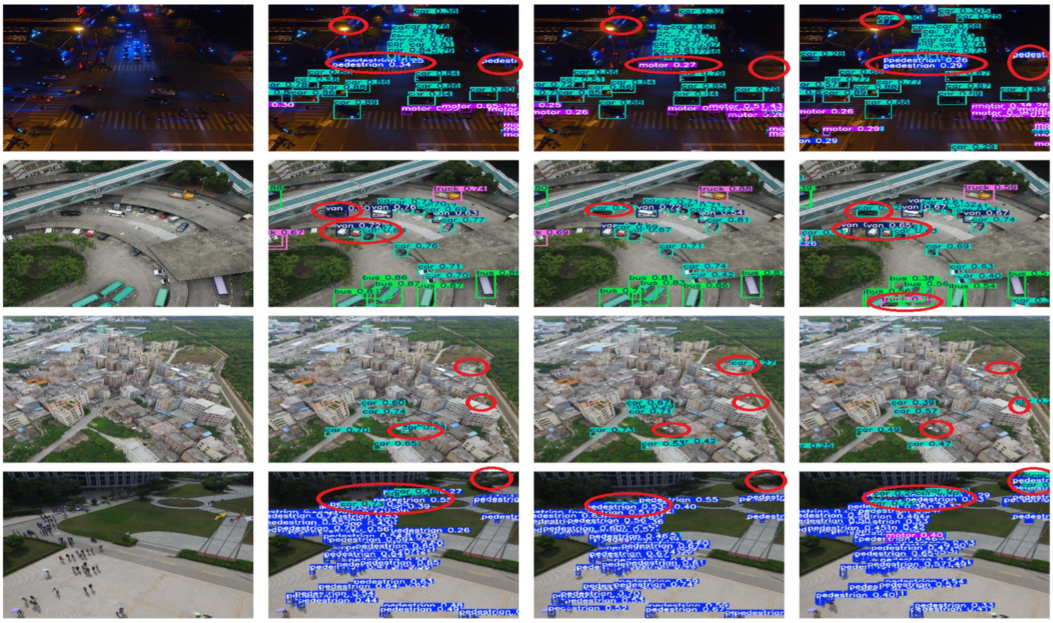

Figure 11. Visualization of detection results on the VisDrone dataset (the columns from left to right are the original image, LMAD-YOLO detection image, YOLO11 detection image, RT-DETR detection image)

5.Presentation:

The logical flow within certain paragraphs couldbe improved, and the language used is not consistently fluid and precise.

The captions and legends of figures and tables lack sufficient information to clearly convey the intended message. For example, Figure 7 lacks detailed explanations of the axes and legends. The titles of Figures 8 and 9 are too simplistic and do not adequately describe the visualized content.

The formatting of the references may exhibit inconsistencies and requires careful review to ensure uniformity and completeness.

Response:We have made comprehensive adjustments to the logical structure and conducted thorough grammatical checks on the manuscript.

The title of Figure 7 has been updated to "Training Dynamics: Precision, Recall, and mAP Curves of LMAD-YOLO vs. YOLO11n," with the x-axis representing 200 training batches and the y-axis denoting evaluation metrics.

The title of Figure 8 has been rewritten as "Feature Map Visualization of SPPF and MSPF_Attention Modules for Small-Target Enhancement."

The title of Figure 9 has been expanded to "Comparison of Two Improved Modules and Output Layer Receptive Fields in the Header Network Between LMAD-YOLO and YOLO11 Models."

Additionally, a comprehensive check was performed on the references to ensure format consistency across all entries.

Reviewer #2: In my opinions, the manuscript has the following deficiencies which cannot be ignored. Language and presentation, should be refined throughout the text, its stylistic and grammatical issues, significantly hinders the readability of the work. Why is the YOLO11n chosen to be improved, there are different editions of YOLO11 models, such as YOLO11m, YOLO11s, YOLO11x, the experimental design is limited. Additionally, more interesting test data, such asevaluation indices, FPS, mAP@0.5:0.95, should be added.

Thank you for your thorough review and constructive feedback. Your comments have greatly helped us refine the manuscript and strengthen its scientific rigor. Below are our point-by-point responses and corresponding revisions:

1. Language and Presentation Refinement

Comment:"The manuscript contains stylistic and grammatical issues that hinder readability."

Response:

We have made comprehensive adjustments to the logical structure and conducted thorough grammatical checks on the manuscript.Key improvements include:

Grammar/Flow: Restructured convoluted sentences (e.g., Section 2.2 methods description) and fixed grammatical errors (e.g., subject-verb agreement in ablation analysis).

Technical Terminology: Unified terms (e.g., "downsampling" instead of "down-sampling," "mAP@0.5:0.95" instead of "mAP50-95").

Passive-to-Active Voice: Revised passive constructions (e.g., "It was observed that..." → "Our experiments demonstrate...") to enhance clarity.

2. Justification for Selecting YOLO11n

Comment:"Why was YOLO11n chosen for improvement over other YOLO11 variants (e.g., YOLO11m, YOLO11s, YOLO11x)?"

Response:

The choice of YOLO11n as the baseline was based on three criteria critical to drone applications:

Computational Efficiency: YOLO11n has the lowest GFLOPS (6.3) among YOLO11 variants, aligning with drones' limited onboard computing resources.

Balanced Performance: Compared to lighter variants (YOLO11s), YOLO11n retains adequate feature extraction capability (mAP@0.5:0.95 = 45.2%) for small targets.

Fair Comparison: To ensure an equitable ablation study, we prioritized optimizing the base model (YOLO11n) rather than larger variants (YOLO11x) whose superior performance might overshadow module-specific contributions.

3. Expanded Experimental Design and Evaluation Metrics

Comment:

"The experimental design is limited. Additional metrics like FPS and mAP@0.5:0.95 should be included."

Response:

We have enriched the experiments with the following additions:

Table 3 (Comparison Experiments): Added:

mAP@0.5:0.95: LMAD-YOLO achieves 46.7%, surpassing YOLO11n (45.2%) and YOLOv12n (45.1%).

FPS: LMAD-YOLO reaches 197 FPS , suitable for real-time drone deployment.

Table 3. comparative experimental results

Model	P/%	R/%	Map50/%	Map50-95/%	GFLOPS	Params (m)	FPS
YOLOV3t	**77.2**	64.8	71.7	42.0	14.3	9.524	99.7
YOLOV5n	71.7	64.7	68.5	42.6	**5.8**	2.182	99.6
YOLOV8n	74.0	66.4	71.2	44.8	6.8	2.685	229.8
YOLOV9t	74.8	66.9	72.1	45.6	6.4	**1.730**	207.4
YOLOV10n	72.5	64.9	69.7	43.5	6.5	2.266	344.1
YOLO11n	74.6	66.5	71.4	45.2	6.3	2.583	226.0
RT-DETR	76.0	67.2	67.8	41.4	42.6	16.756	64.8
YOLOv12n	75.4	65.8	71.6	45.1	6.3	2.55	**347.7**
LMAD-YOLO	77.0	**67.9**	**73.6**	**46.7**	6.6	2.422	197.3

Table 4. Generalization experiments on VisDrone datasets

Model	P/%	R/%	Map50/%	Map50-95/%	FPS
YOLOv3t	33.3	20.8	18.3	9.8	164.0
YOLOv5n	35.7	28.0	24.9	13.7	125.4
YOLOv8n	38.8	28.8	26.4	14.6	129.1
YOLOv9t	37.6	29.4	26.8	**15.2**	121.7
YOLOv10n	37.7	**30.3**	**27.1**	14.8	**277.7**
LOYO11n	37.6	28.5	26.0	14.4	119.9
RT-DETR	**40.5**	23.4	20.7	10.8	46.92
YOLOv12n	37.4	28.7	26.3	14.7	139.9
LMAD-YOLO	39.3	29.4	26.8	14.9	131.9
