# Supplementary material for: LMAD-YOLO: A vehicle image detection algorithm for drone aerial photography based on multi-scale feature fusion
Source: PLoS One. 2025 Jul 15;20(7):e0328248. doi: 10.1371/journal.pone.0328248 (PMC12262879; doi:10.1371/journal.pone.0328248)
Supplement: Attachment — Submitted filename: comment for plos one12249.doc [file pone.0328248.s002.doc]

Dear editors,

Thank you for your patience. My comments are listed as followings.

In my opinions, the manuscript has the following deficiencies which cannot be ignored. Language and presentation, its stylistic and grammatical issues, significantly hinders the readability of the work. There are different editions of YOLO11 models, such as YOLO11m, YOLO11s, YOLO11x, why is the YOLO11n chosen, and the experimental design is limited. More interesting test data, such as evaluation indices, FPS, [mAP@0.5:0.95](mailto:mAP@0.5:0.95), should be added.

Sincerely,

Jinhui Zhao
